# Parallel analysis of RNA ends enhances global investigation of microRNAs and target RNAs of *Brachypodium distachyon*

**DOI:** 10.1186/gb-2013-14-12-r145

**Published:** 2013-12-24

**Authors:** Dong-Hoon Jeong, Skye A Schmidt, Linda A Rymarquis, Sunhee Park, Matthias Ganssmann, Marcelo A German, Monica Accerbi, Jixian Zhai, Noah Fahlgren, Samuel E Fox, David F Garvin, Todd C Mockler, James C Carrington, Blake C Meyers, Pamela J Green

**Affiliations:** 1 Department of Plant and Soil Sciences and Delaware Biotechnology Institute, University of Delaware, Newark, DE 19711, USA; 2 Donald Danforth Plant Science Center, St Louis, MO 63132, USA; 3 Department of Botany and Plant Pathology, Oregon State University, Corvallis, OR 97322, USA; 4 USDA-ARS Plant Science Research Unit, University of Minnesota, St Paul, MN 55108, USA; 5 Current address: Monsanto Company, Chesterfield, MO 63017, USA; 6 Current address: IBACON GmbH, Rossdorf, Germany; 7 Current address: Dow AgroSciences LLC, Portland, OR 97224, USA; 8 Current address: Linfield College, McMinnville, OR 97128, USA

## Abstract

**Background:**

The wild grass *Brachypodium distachyon* has emerged as a model system for temperate grasses and biofuel plants. However, the global analysis of miRNAs, molecules known to be key for eukaryotic gene regulation, has been limited in *B. distachyon* to studies examining a few samples or that rely on computational predictions. Similarly an in-depth global analysis of miRNA-mediated target cleavage using parallel analysis of RNA ends (PARE) data is lacking in *B. distachyon*.

**Results:**

*B. distachyon* small RNAs were cloned and deeply sequenced from 17 libraries that represent different tissues and stresses. Using a computational pipeline, we identified 116 miRNAs including not only conserved miRNAs that have not been reported in *B. distachyon*, but also non-conserved miRNAs that were not found in other plants. To investigate miRNA-mediated cleavage function, four PARE libraries were constructed from key tissues and sequenced to a total depth of approximately 70 million sequences. The roughly 5 million distinct genome-matched sequences that resulted represent an extensive dataset for analyzing small RNA-guided cleavage events. Analysis of the PARE and miRNA data provided experimental evidence for miRNA-mediated cleavage of 264 sites in predicted miRNA targets. In addition, PARE analysis revealed that differentially expressed miRNAs in the same family guide specific target RNA cleavage in a correspondingly tissue-preferential manner.

**Conclusions:**

*B. distachyon* miRNAs and target RNAs were experimentally identified and analyzed. Knowledge gained from this study should provide insights into the roles of miRNAs and the regulation of their targets in *B. distachyon* and related plants.

## Background

The wild grass *Brachypodium distachyon* (*Brachypodium*) belongs to the Pooideae family of grasses and has emerged as a model system for temperate grasses including agronomically important crops such as wheat (*Triticum monococcum*, *T. durum* and *T. aestivum*), barley (*Hordeum vulgare*) and oat (*Avena sativa*), as well as for biofuel plants such as switchgrass (*Panicum virgatum*) and *Miscanthus* (*Miscanthus* x *giganteus*) [1,2]. It has many attractive features that resemble those of other models like *Arabidopsis* (*Arabidopsis thaliana*) and rice, including a relatively small and sequenced diploid genome, a well-developed transformation system and a growing bank of available Transfer-DNA insertional mutants [3-6]. Furthermore, as a wild grass, *Brachypodium* has never been subjected to human selection, which has drained the genetic diversity of domesticated cereal grasses. For these reasons, *Brachypodium* is a promising model system among temperate grasses for the identification of traits that may have been lost during domestication. In addition, phylogenetic surveys indicate that some important cereal crops, such as wheat and barley, are much more closely related to *Brachypodium* than they are to rice, sorghum or maize [7], and therefore studies involving *Brachypodium* are easily translatable and more applicable to these crops than studies of other model systems.

*Brachypodium* is also proposed as a model system for biofuel crops for understanding topics such as the characteristics of the grass cell wall involved in the process of saccharification [8]. However, despite its increasing importance as a model system, many areas of fundamental research in *Brachypodium* are underdeveloped, such as that related to microRNAs (miRNAs) and their targets.

miRNAs are critical regulators of the expression of many specific target genes involved in development, stress and other processes [9-11]. *MIRNA* genes are transcribed by RNA polymerase II, and processed by a Dicer-like (DCL) protein from stem-loop structures. Mature miRNAs are loaded onto Argonaute (AGO) proteins to form RNA-induced silencing complexes (RISCs) and perform target RNA regulation by base pairing [12,13]. Although it is reported that some plant miRNAs inhibit target mRNA translation [14-17] or direct DNA methylation of target genes [18], the majority of plant miRNAs, once loaded, guide RISCs to cleave their target RNAs, which are subsequently degraded. In some cases, miRNA-mediated cleavage of a target RNA can trigger the production of secondary small interfering RNAs (siRNAs), typically comprising trans-acting siRNAs (tasiRNAs) and/or phased siRNAs (phasiRNAs) [19-21]. The secondary siRNAs generated from a limited number of primary targets can silence large gene families such as *Pentatricopeptide Repeat* (*PPR*) genes in *Arabidopsis* or *Nucleotide Binding Site-Leucine-Rich Repeat* (*NBS-LRR*) genes in other dicot plants [21-24]. Other than the tasiRNAs generated by the 21-nucleotide miR390 [25], most of the other tasiRNAs (or phasiRNAs) are generated by 22-nucleotide-long miRNAs [26-29]. In rice and *Brachypodium*, 22-nucleotide miR2118 and miR2275 can trigger 21-nucleotide and 24-nucleotide phasiRNA generation, respectively, from hundreds of non-coding loci of unknown function [30-32].

In diverse plants and samples, numerous miRNAs have been identified and profiled using deep sequencing and bioinformatic tools [33]. miRNA prediction from deep-sequencing data relies on the unique features of plant miRNAs among the other types of small RNAs [21,34-36]. Currently, more than 5,100 miRNA precursors from 67 plant species have been annotated and deposited in miRBase [37]. In *Brachypodium*, 136 mature miRNAs from 135 miRNA precursors have been identified from different tissues and drought- or cold stress-treated tissues [38-42]. However, the number of annotated miRNAs for *Brachypodium* is relatively low compared to other model plants, such as the 338 miRNAs for *Arabidopsis* and 708 miRNAs for rice. Annotation of miRNAs from the other temperate grasses and biofuel plants is also very limited. Moreover, there have been rising concerns about incorrect plant miRNA annotation. These misannotations were mostly due to computational analysis without experimental validation. For example, by adopting the recently updated plant miRNA criteria [36], re-evaluation of about 400 miRBase-annotated rice miRNAs via a large-scale analysis revealed that about 150 had characteristics more typical of siRNAs [34]. Thus, for the continued development of *Brachypodium* as a model plant, it is a worthwhile challenge to identify with high confidence a more complete set of miRNAs.

Similarly, to understand the function of miRNAs, the identification of their mRNA targets, preferably with experimental support, is essential. Computational prediction of target RNAs relies on the feature of complementary base pairing between miRNA and target RNA pairs. Parallel analysis of RNA ends (PARE) is a technique for RNA degradome analysis that provides high-throughput experimental evidence of miRNA-mediated target cleavage [43-45]. Using PARE, target RNA cleavage products can be cloned and deeply sequenced. This has been a powerful approach for global target identification and validation in *Arabidopsis*, legumes, rice and a few other plants [21,43-52]. The miRNA-mediated target cleavage is an endonucleolytic site-specific cleavage that cuts within the target RNA strand of the miRNA-target RNA duplex between nucleotides 10 and 11 relative to the 5′ end of the miRNA sequence. The single-nucleotide resolution of PARE data can therefore provide detailed information about site-specific cleavages guided by the individual miRNAs in the samples examined. Of temperate grasses and biofuel plants, only barley has been studied with a focus on global target identification using PARE data [52]. Although the generation and analysis of PARE data from *Brachypodium* was recently reported for the study of general mRNA decay [53], miRNA-mediated cleavage was only briefly presented.

In this study, we investigated *Brachypodium* miRNAs by deeply sequencing 17 small RNA libraries from various tissues and environmentally stress-treated plants. Using a computational pipeline, we identified not only conserved miRNAs that have not been reported in *Brachypodium*, but also non-conserved miRNAs that were not found in other plants. In addition, we identified a new regulation of miR5200 under submergence stress. Finally, we identified many miRNA target cleavages using four PARE libraries from key tissues. The knowledge gained from this study should be beneficial for understanding the role of miRNAs and their target regulation in *Brachypodium* and related temperate grasses and biofuel plants.

## Results

### Massive sequencing of *Brachypodium* small RNAs

To enhance the knowledge and understanding of the small RNA population in *Brachypodium*, a very large set of small RNAs was cloned and deeply sequenced with Illumina technology. In total, 17 libraries were constructed and sequenced to achieve a broad coverage of small RNAs in various tissues and stresses (Table 1). The plant material is described in Materials and methods. Briefly, eight tissue libraries are from root, seedling, stem, leaf and stem tissues, and two biological replicates of leaf and panicle tissues. Nine stress libraries include drought, salt, cold, heat, submergence treatments and their control, phosphate-starvation and its control, and a pool of abiotic, biotic, and mechanical stresses. After trimming the adapter sequences, a total of 94,101,750 reads were obtained and aligned against the *Brachypodium* genome [31] using Bowtie. About 80%, or 75,048,757, matched perfectly to the *Brachypodium* genome. This resulted in an average abundance of approximately 4.4 million and approximately 1.2 million distinct genome-matched small RNA sequences per library. To our knowledge, the population sequenced in this paper is the largest such effort to date for *Brachypodium* and greatly enhances the small RNA resources for this plant*.*

**Table 1:** Summary statistics of small RNA libraries

			**Raw trimmed**		**Genome matched**
**Tissue or stress**	**Code**	**Abundance**^**a**^	**Distinct**^**b**^	**Abundance**^**c**^	**Distinct**^**d**^
Root	BDI08	5,103,699	1,055,138	3,942,845	597,486
Seedling	OBD03	2,624,702	1,263,576	1,566,530	548,313
Leaf 1	BDI04	5,207,568	1,425,013	4,305,921	959,519
Leaf 2	BDI09	6,991,777	736,393	5,315,520	270,687
Stem	BDI06	3,855,537	1,926,121	3,209,322	1,435,137
Leaf and stem	OBD02	3,603,581	1,633,352	2,316,244	739,198
Panicle 1	BDI05	4,944,182	2,166,081	4,102,150	1,567,160
Panicle 2	OBD01	3,992,373	2,135,020	2,783,725	1,191,806
Shoot control for stress	BDI02	2,494,849	1,077,185	1,975,853	800,615
Drought-stressed shoot	BDI03	1,351,288	515,704	1,038,168	351,245
Salt-stressed shoot	BDI17	8,498,042	2,149,559	7,275,111	1,542,354
Cold-stressed shoot	BDI01	722,947	379,189	538,881	260,131
Heat-stressed shoot	BDI18	8,861,608	2,936,249	7,336,506	2,042,837
Submergence-stressed shoot	BDI19	9,421,125	4,336,814	7,586,861	3,037,780
Shoot control for low phosphate	BDI15	11,491,988	2,924,520	9,510,554	2,086,843
Phosphate-starved shoot	BDI16	12,405,206	2,977,218	10,490,572	2,153,426
Pooled stresses	OBD04	2,531,278	1,133,267	1,753,994	590,660
Total^e^		94,101,750	21,441,607	75,048,757	12,108,240

^a^Raw-trimmed abundance is the number of sequences obtained after trimming. ^b^Raw-trimmed distinct is the number of different sequences. ^c^Genome-matched abundance is the number of sequences that match perfectly to the genome. ^d^Genome-matched distinct is the total number of different sequences that match to the genome. ^e^The totals of the abundance columns are sums and the totals of the distinct columns are unions (to give the number of non-redundant sequences).

### Identification of *Brachypodium* microRNAs and microRNA precursors

**Figure 1:**
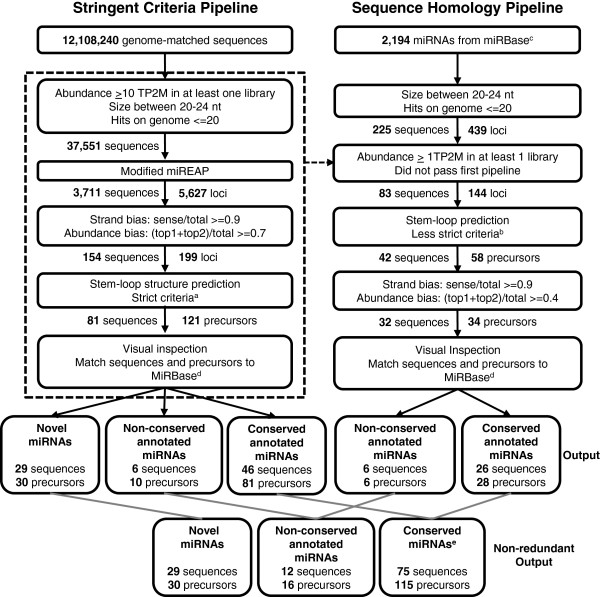
**Pipeline for the prediction of miRNAs.** miRNAs were identified by a series of filters. Black arrows are used to indicate the small RNAs that passed the criteria. The dashed arrow indicates small RNAs that failed to pass the criteria. Numbers of candidate miRNAs and precursors after each filter are indicated. Gray lines indicate where data from two pipelines has been combined. ^a^Strict criteria are ≤4 mismatches + bulges in the miRNA:miRNA* pairing and a bulge size of ≤1 nucleotide. ^b^Less strict criteria are defined as ≤8 mismatches + bulges in the miRNA:miRNA* pairing. ^c^Non-redundant miRNAs from miRBase Release 18. ^d^Up to two nucleotide mismatches from miRBase Release 19 were allowed. ^e^Conserved miRNAs include manually identified miR444 precursors and miR2218 precursors. miRNA, microRNA; nt, nucleotide; TP2M, transcripts per 2 million.

Although many miRNAs have been identified from *Brachypodium*, the large set of small RNAs in this study provides a tremendous opportunity for identification of new miRNAs. In a recent release of miRBase (Release 19), a total of 135 miRNA precursors have been annotated for *Brachypodium*[37]. Since miRNA identification for *Brachypodium* is not saturated yet compared to other well-studied plants, such as *Arabidopsis* and rice, we applied a computational pipeline adapted from our recent reports [21,34] to identify more miRNAs and their precursors. Out of approximately 12 million genome-matched distinct small RNA sequences, approximately 37,500 sequences passed the first filter, which consists of cut-offs for abundance, size and hits to the genome (Figure 1). These sequences were then evaluated for miRNA:miRNA-star (miRNA*) pairing, and 3,711 miRNA candidates were extracted from 5,627 putative miRNA precursor loci. In a third filter, strand bias and abundance bias cut-offs were used to distinguish the miRNA precursors from siRNA loci, resulting in 154 sequences from 199 loci. Finally, stem-loop structures of miRNA precursor candidates were visually inspected to ensure accuracy and 81 miRNAs generated from 121 precursors were identified (Additional file 1: Table S1). Excluding 52 miRNAs that were previously annotated for *Brachypodium* or similar to known plant miRNAs, 29 miRNAs from 30 precursors were newly identified in *Brachypodium* (Table 2).

**Table 2:** Newly identified microRNAs

**Family**	**Mature microRNA**	**Sequence**	**Length**	**Abundance**^**a**^
5163	Bdi-miR5163b-3p	UAGAUAUUUCAGGUUGUGUGGA	22	36,348
5181	Bdi-miR5181e	CGACACUUACUGUGGCUCGGA	21	277
5185	Bdi-miR5185l-3p.2	UGGAGAUUGACUUAGAAGCGG	21	360
7731	Bdi-miR7731	AACAAGGGAUGCACAUACUUUGAG	24	836
7738	Bdi-miR7738-3p	GUGCUUGACAGACGACUCUGG	21	446
7754	Bdi-miR7754-3p	UUCUCUCGGCUAAGGAACUGC	21	392
9480	Bdi-miR9480ab	UAUGUGAGGGUGGUAACUGAA	21	1,645
9481	Bdi-miR9481a	UCAGUCGGAUUUCUCACCUUCGAA	24	384
	Bdi-miR9481b	UCAGUCGGAUUUCUCACCUUC	21	73
9482	Bdi-miR9482	CCUUUGGGGAAGAAGGGAAAC	21	339
9483	Bdi-miR9483ab	UUGAACUGUUUCCUCUGAAGUUCC	24	317
9484	Bdi-miR9484	UAGUGCAGGGAGAAGUCGGUC	21	259
9485	Bdi-miR9485	UUAUGACGUGUAGGAGUUGCA	21	260
9486	Bdi-miR9486a	AUGCUUUCAAGGGAUUAGAGGUUC	24	254
	Bdi-miR9486a.2	UCUAAUGGCUGAAAUGGGAAG	21	183
	Bdi-miR9486b	UAAGUGAUUAGAGGUUCCAGU	21	121
9487	Bdi-miR9487	CCUUGUUCGAUUGCAAGAUGA	21	174
9488	Bdi-miR9488	UGAGGGCUAGGCUUUUAUGUAA	22	161
9489	Bdi-miR9489	UCAGCUCCACGGACUUGGUGA	21	144
9490	Bdi-miR9490	AGGCCACACCCUAAUGGUCGUGCG	24	111
9491	Bdi-miR9491	UGGUAUGUUACCUCUGAUCAG	21	69
9492	Bdi-miR9492	UAUCUACUCUGUCAUGGUAUC	21	60
9493	Bdi-miR9493	AAGAAUUAUGAAACGAAGGGAGUA	24	60
9494	Bdi-miR9494	UUCAUCACCUUCGUCUCCGUC	21	56
9495	Bdi-miR9495	UGAAAAAUGCCUCUGGACGUG	21	55
9496	Bdi-miR9496	CUGGUUGGGCUUAGAUGGGUCC	22	44
9497	Bdi-miR9497	UUUCUGAAUACAUGGUGUAUC	21	35
9498	Bdi-miR9498	GACCGUCAAGUGGUUGUUGAG	21	23
9499	Bdi-miR9499	CCCUCGUCGACGCGGCAGCUC	21	86

^a^Abundance is from the sum of TP2M values from all libraries.

TP2M, transcripts per 2 million.

Because a pipeline approach designed to identify new miRNAs and precursors must be stringent enough to minimize the misannotation of false miRNAs, by design it will invariably miss some of the conserved miRNAs. Therefore, a homology pipeline with less strict criteria and manual analysis was developed to look for known miRNAs that did not follow all of the stringent criteria detailed in the first pipeline. Specifically, small RNA sequences that failed the first pipeline but matched previously annotated plant miRNAs were re-evaluated (Figure 1). This pipeline allowed less abundance and less strict stem-loop structures, and it had a lower cut-off value for abundance bias. Additionally, members of the miR444 family (Additional file 2: Figure S1) and miR2118 were added manually. This miRNA family has an intron in the precursor, which interferes with the folding filters in the pipelines. Together with conserved miRNAs identified from the previous, stringent pipeline, in total 75 conserved miRNAs generated from 115 precursors were identified. In total, we identified 116 mature miRNAs generated from 161 precursors in *Brachypodium*.

### miR162 may not be functional in *Brachypodium*

**Figure 2:**
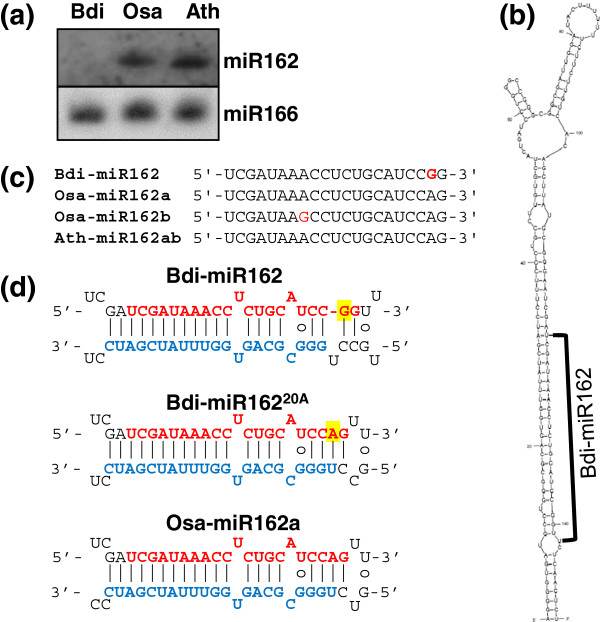
**Characteristics of Bdi-miR162. (a)** RNA blot of miR162 for *Brachypodium* (Bdi), rice (Osa) and *Arabidopsis* (Ath). Mixed probes of P^32^-end labeled Bdi-miR162 and Osa-miR162a were used to detect mature miR162 from *Brachypodium* panicle, rice panicle and *Arabidopsis* flower tissues. miR166 was used as a control. **(b)** Predicted secondary structure of the Bdi-MIR162 precursor. The location of Bdi-miR162 is indicated with a black line. **(c)** Sequence alignment of miR162 for *Brachypodium*, rice and *Arabidopsis*. Nucleotides that differ are shown in red. **(d)** Comparison of base pairings in the folded MIR162 precursor of *Brachypodium* and rice. Base pairing in a mutated Bdi-MIR162 precursor is shown between them. Position 20 of Bdi-miR162 is highlighted in yellow. miRNA is shown in red and miRNA* in blue. miRNA, microRNA.

We observed that miR162, one of the well-conserved miRNAs in plants, had quite low abundance in *Brachypodium*. In our data, miR162 was sequenced just once in a small RNA library from submergence-treated seedlings out of 17 libraries. We validated the lack of miR162 expression using an RNA blot (Figure 2a). While miR162 was highly expressed in rice and *Arabidopsis*, *Brachypodium* miR162 was not detected. One miR162 precursor was found in the *Brachypodium* genome (Figure 2b). Compared to the miR162 found in rice and *Arabidopsis*, the adenine (A) typically found at the 20th nucleotide, was replaced by a guanine (G) in Bdi-miR162 (Figure 2c). To see the impact of this substitution, we compared the miRNA:miRNA* pairing structure of authentic Bdi-miR162 with a hypothetical Bdi-miR162 with an A at the 20th nucleotide position (Bdi-miR162^20A^ in Figure 2d). Substitution of the G by an A at the 20th nucleotide position of the hypothetical Bdi-miR162 (Bdi-miR162^20A^) increased the stability of the miRNA:miRNA* pairing. There was no bulge and the structure was similar to the stable structure of the Osa-miR162a:miR162* pairing in rice. Furthermore, there was no evidence of cleavage in its predicted target, the *Brachypodium DCL1* homolog, in our PARE data. These data imply that the altered sequence of *Bdi-MIR162* may affect miRNA processing and cause the reduced level of Bdi-miR162, resulting in little or no *DCL1* cleavage.

### Alternative folding generates microRNAs by stem-sharing

**Figure 3:**
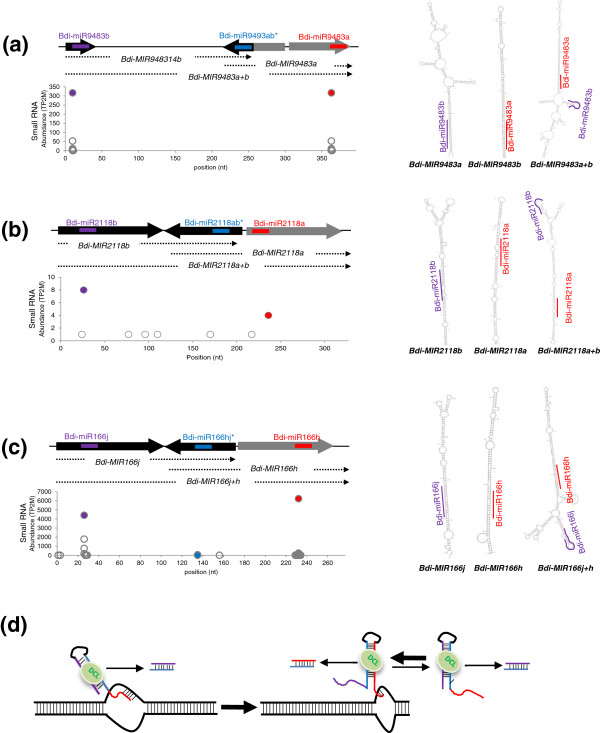
**Alternative RNA folding generates miRNAs by sharing one arm of a stem.** Examples of alternative RNA folding in precursors of Bdi-miR9483 **(a)**, Bdi-miR2118 **(b)** and Bdi-miR166 **(c)**. Shown are schematic diagrams of adjacent miRNA precursors that share one arm of a stem. miRNA placement along the precursor is indicated with arrows, the middle arrow representing the shared arm of two miRNA precursors and the abundance of small RNAs matching each position is plotted below. Purple and red lines and filled circles indicate two mature miRNAs corresponding to each precursor and the blue line and filled circle indicates the miRNA*. Predicted secondary structures from each precursor, as well as that from the full sequence, are shown in the right panel. **(d)** Model for alternative RNA folding generating miRNAs by sharing one arm of a stem. The nascent transcript preferentially processed by DCL activity generates the first miRNAs (purple). After transcription, two possible RNA folding structures are responsible for generating the two mature miRNAs (purple and red). DCL, Dicer-like protein; miRNA, microRNA; TP2M, transcripts per 2 million.

Clustering of miRNA genes is less common in plants than in animals [54]. About 10% to 15% of annotated miRNAs in rice, *Arabidopsis* and poplar are found in tandem clusters at a distance of less than 3 kb [55]. Most are polycistronic miRNA precursors that encode identical mature miRNAs. We also identified several miRNA precursors that are located in tandem in the *Brachypodium* genome. Interestingly, we noticed that two independent miRNA precursors overlapped each other in some miRNA clusters including *Bdi-MIR9483ab*, *Bdi-MIR2118ab* and *Bdi-MIR166hj* (Figure 3a,b,c). In these cases, one arm of a stem is shared by two adjacent miRNA precursors and serves as a common miRNA* strand. In these tandem-overlapping hairpins, the second hairpin structures are more stable and favored than the first. Thus, if the RNA secondary structure of the entire locus is predicted, the second miRNA precursor is preferred and the first one loses the hairpin structure. Our small RNA data indicate that both overlapping miRNA precursors may generate mature miRNAs. It is not clear whether *Bdi-MIR9483a* and *Bdi-MIR9483b* are equally functional since they encode identical mature miRNAs (Figure 3a). However, in the cases of the *Bdi-MIR2118ab* and *Bdi-MIR166hj* clusters, it is apparent that both of the precursors form stem-loop structures and are processed to generate their own mature miRNAs, due to distinct sequences of mature miRNAs and their expression. Two sets of mature miRNA were expressed at different levels in *Bdi-MIR2118ab* and *Bdi-MIR166hj* loci (Figure 3b,c). We hypothesized a model that alternative RNA folding of overlapped miRNA precursors generates miRNAs by sharing one arm of a stem (Figure 3d). In this model, the nascent transcript preferentially folds into the first stem-loop structure and is processed to generate the first mature miRNA. After transcription, both of the two possible RNA folding structures may fold to generate both of the respective mature miRNAs.

### Validation of stress-responsive microRNAs

**Figure 4:**
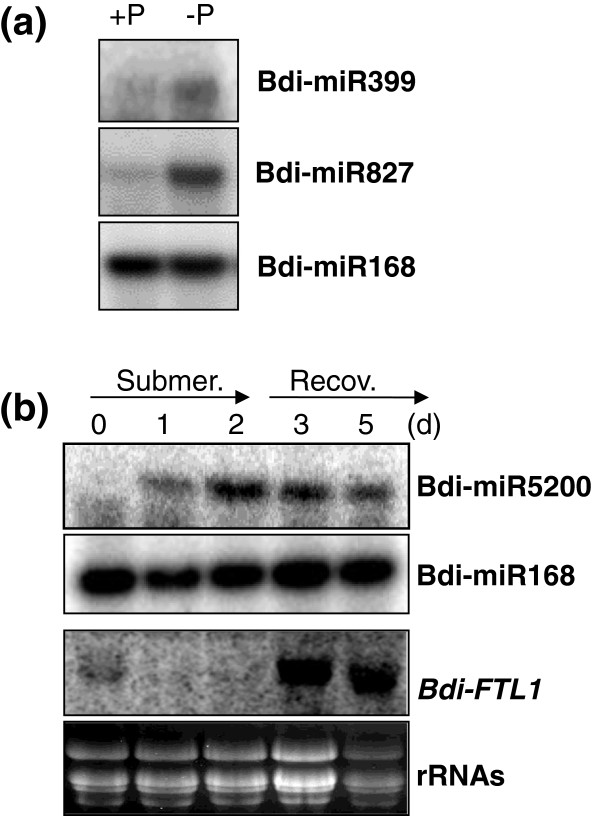
**Validation of stress-responsive miRNAs. (a)** Validation of phosphate-starvation inducible Bdi-miR399 and Bdi-miR827 by splinted ligation-mediated miRNA detection. Bdi-miR168 was used as a control. **(b)** Expression pattern of Bdi-miR5200 during submergence treatment and subsequent recovery. Three-week-old seedlings were subjected to submergence stress by completely immersing the plants in water, and subsequent recovery upon removal from the water. Shoots were collected before submergence treatment (0 d), after one-day (1 d) or two-day (2 d) treatments, and one (3 d) or three (5 d) days after recovery. Then 20 μg of total RNA were used to detect *Bdi-FTL1* using an RNA blot. Bdi-miR168 and rRNAs were used as controls. miRNA, microRNA; Recov., recovery; rRNA, ribosomal RNA; Submer., submergence.

To identify stress-regulated miRNAs, the sequencing frequency of miRNAs was compared between each stress-treated sample and its control library. We first examined how well-known and well-conserved examples of stress-regulated miRNAs are represented in our *Brachypodium* small RNA libraries. For example, miR399 and miR827, which are known to be induced under phosphate starvation conditions in *Arabidopsis*[56-60] and rice [34,58,61,62], were highly represented in the phosphate-starved shoot library compared to its control library by about 9- and 4-fold, respectively. The upregulation in both cases was further validated by using the splinted ligation-mediated miRNA detection method (Figure 4a). Plants grown in a phosphate-depleted Murashige and Skoog (MS) agar media for 3 weeks accumulated both more miR399 and more miR827 than plants grown in a MS agar media.

Next, we investigated miRNA expression using stress treatment and found that miR5200 was submergence-inducible; it was 80 times more represented in the submergence library compared to the control. To validate this regulation experimentally, the expression of miR5200 in response to submergence treatment and subsequent recovery was examined. Accumulation of miR5200 increased under submergence conditions and slowly decreased as the plants recovered from the submergence (Figure 4b). An *FT* (*Flowering time locus T*)-*like* gene, which is a predicted target of miR5200, was downregulated during the submergence treatment while miR5200 was upregulated. In addition, *Bdi-FTL1* mRNA abundance increased during the recovery from the submergence. This result implies that miR5200 may play a role in regulating the flowering time under submergence conditions.

### Identification of cleaved microRNA targets on a global scale

To investigate the targets of *Brachypodium* miRNAs, our strategy was to use the PARE approach to sequence deeply RNA decay intermediates that have a 5′ monophosphate and a 3′ poly(A) tail. This is the structure of the downstream fragments resulting from miRNA-guided cleavage. In *Arabidopsis*, PARE captured the vast majority of these cleavage products, which had been previously validated in independent experiments [44]. For *Brachypodium*, PARE libraries were constructed from RNA from four key organs. As shown in Table 3, these libraries were sequenced to a total depth of nearly 70 million reads. Of these, nearly 61 million matched the genome resulting in a non-redundant set of more than 5 million PARE sequences. These libraries represent a rich resource of PARE (or RNA degradome) data, which previously had been lacking for *Brachypodium*.

**Table 3:** Summary statistics of PARE libraries

		**Trimmed**		**Genome matched**		**cDNA matched**		**cDNA loci**^**g**^
**Tissue**	**Code**	**Total**^**a**^	**Distinct**^**b**^	**Total**^**c**^	**Distinct**^**d**^	**Total**^**e**^	**Distinct**^**f**^	
Root	BDI20	10,748,481	2,342,429	9,279,374	1,605,048	4,298,418	1,021,275	22,762
Leaf	BDI21	13,772,836	1,238,807	12,266,285	684,817	4,930,319	391,613	20,333
Stem	BDI23	16,367,640	3,847,003	12,740,059	1,422,289	5,534,630	656,849	24,168
Panicle	BDI25	29,050,139	4,820,607	26,566,704	3,419,993	15,078,495	2,203,450	20,538
**Total**		**69,939,096**		**60,852,422**	**5,202,600**	**29,841,862**	**3,165,429**	**25,129**

^a^The total number of sequences obtained from a single run after trimming. ^b^The number of different sequences found in a set after trimming. ^c^The total number of sequences that match the genome at least once. ^d^The number of different sequences that match the genome at least once. ^e^The total number of sequences that match the sense strand of the coding sequence BdiCDSv1.2, the coding sequence minus UTR, at least once. ^f^The number of different sequences that match the sense strand of the coding sequence at least once. ^g^The total number of cDNA loci with at least one matching PARE sequence.

PARE, parallel analysis of RNA ends; UTR, untranslated region.

To identify miRNA target cleavage events, we first generated a set of predicted target cleavage sites for 116 *Brachypodium* miRNAs and then examined all the distinct PARE sequences for those that precisely matched these predictions. Two prediction programs using different alignment algorithms were used to predict a large set of potential targets. Specifically, psRNATarget and CleaveLand (with the native and a modified scoring system) were used, as described in Materials and methods. These programs identified a total of 2,141 predicted miRNA targets in transcripts corresponding to 1,968 genes (loci) as indicated in Table 4. As shown in Additional file 2: Figure S2, psRNATarget and the two versions of CleaveLand each generated unique targets and thus a larger set of combined potential target cleavage sites then any one alone. When the total set of predicted targets cleavage sites were matched to the PARE data, 262 of the sites corresponding to 246 distinct coding regions (CDSs) had PARE sequences starting precisely at the predicted site, that is, between nucleotides 10 and 11 (from the 5′ end of the miRNA) of the miRNA-target RNA duplex, in at least one of the four tissue libraries (Table 4). The individual targets are listed in Additional file 1: Table S3, which also includes which programs predicted each target and the corresponding target scores.

**Table 4:** microRNA targets with PARE sequences at the expected site

**Prediction programs**	**Predicted targets**		**Targets with precise PARE**^**a**^	
	**Sites**^**b**^	**Loci**^**c**^	**Sites**^**b**^	**Loci**^**c**^
CleaveLand	1,033	967	206	154
CleaveLandM^d^	794	746	170	127
psRNATarget	1,225	1,153	211	149
Total^e^	2,141	1,968	262	246

^a^PARE sequences that start at the precise cleavage site predicted. ^b^Distinct cleavage sites. ^c^The total number of genes the cleavage sites correspond to. ^d^CleaveLand with the modified scoring systems described in Materials and methods. ^e^The number of (non-redundant) sites or loci.

PARE, parallel analysis of RNA ends.

### A computational approach for characterizing microRNA target cleavage sites supported by PARE data

Since this study is the first in-depth analysis of cleaved *Brachypodium* miRNA targets, we sought to examine each target that had a PARE sequence precisely at the expected cleavage site for characteristics associated with the cleavage of well-studied miRNA targets in *Arabidopsis*. Hence, we developed a pipeline to evaluate cleaved miRNA targets for three parameters related to the prominence of the cleavage products, which is discussed in more detail below: abundance, rank and peak percentage. Our approach was first to establish cut-off criteria for each of these parameters using 137 *Arabidopsis* miRNA targets that had been experimentally validated for miRNA-guided cleavage, independent of PARE (Additional file 1: Table S4). The second step was to use these prominent criteria to characterize miRNA target cleavage events in *Brachypodium*.

**Figure 5:**
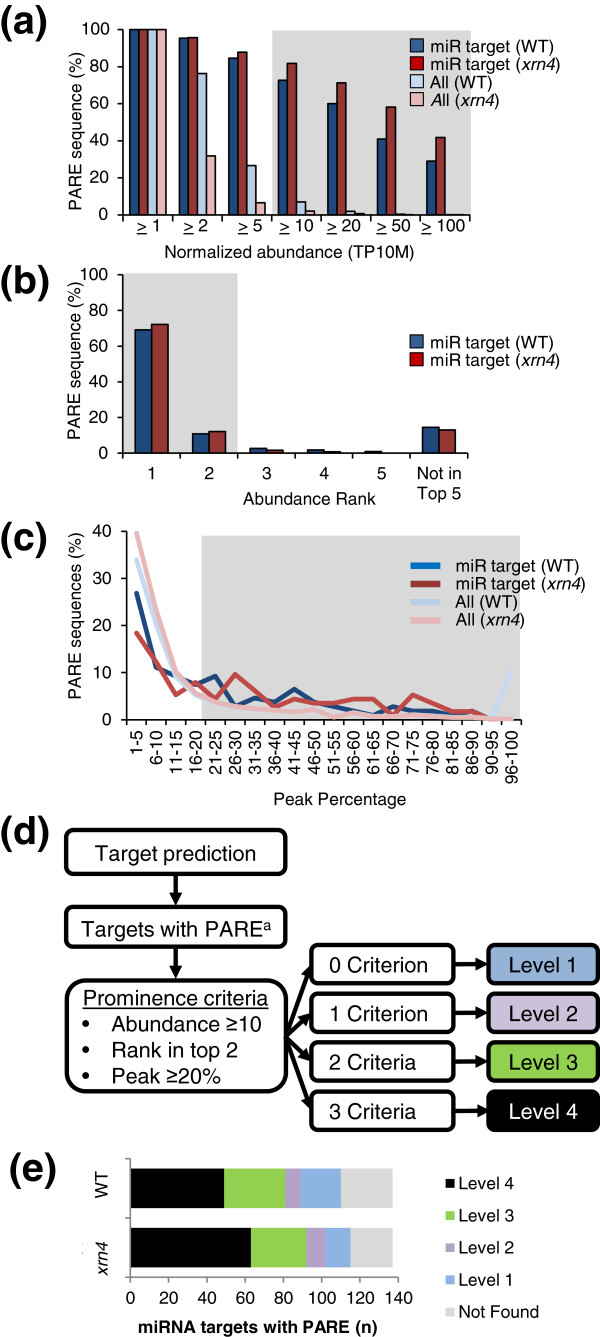
**Examination of *Arabidopsis *PARE libraries using independently validated miRNA targets to establish criteria cut-offs.** Comparisons of validated *Arabidopsis* miRNA targets and total genome-matched sequences in WT and *xrn4* are shown. Histograms in (a) and (b) and lines in (c) show the percentages of PARE sequences matching miRNA target cleavage sites or all the PARE sequences from WT and *xrn4* PARE libraries. Gray areas in (a), (b) and (c) indicate the selected cut-offs. **(a)** Distribution of PARE sequences by normalized abundance. **(b)** Distribution of PARE sequences by abundance rank in an annotated transcript for miRNA targets. **(c)** Distribution of PARE sequences by the peak percentage in annotated transcripts for miRNA targets or all genes. **(d)** A flowchart for evaluating miRNA target cleavage sites based on the three prominence criteria. ^a^miRNA targets with PARE sequences at the precise canonical location. **(e)** Distribution of validated miRNA targets in WT and *xrn4* according to level. miRNA, microRNA; PARE, parallel analysis of RNA ends; TP10M, transcripts per 10 million; WT wild type.

We analyzed two sets of previously published *Arabidopsis* PARE data that were generated from the inflorescence tissues of Col-0 wild type (WT) and an *xrn4* mutant [44]. XRN4 degrades the downstream fragments resulting from miRNA-mediated cleavages so the lack of this enzyme in the *xrn4* mutant improved the sensitivity of PARE in *Arabidopsis*[44,63]. The first parameter evaluated was the abundance of the PARE sequence at the target cleavage site. Often, miRNA-mediated cleavage at the precise cleavage site within a target mRNA occurs at a higher frequency than non-specific mRNA degradation events, and therefore the abundance of the PARE sequence at that site is higher than background. Figure 5a shows that most PARE sequences in the *Arabidopsis* samples had low abundance, while only approximately 2% to 7% of PARE sequences were more abundant than 10 TP10M. However, 73% to 82% of PARE sequences matching miRNA-mediated cleavage sites were at least 10 TP10M. Thus, a minimum abundance of 10 TP10M was chosen as the cut-off criterion for this parameter. The second parameter evaluated was the abundance rank within a given target mRNA (Figure 5b). Between 80% and 84% of the *Arabidopsis* miRNA-guided cleavages were either the most abundant or the second-most abundant (within the top two) PARE sequences in the annotated mRNA. An abundance rank of occurrence within the top two most abundant sequences within the mRNA was set as the cut-off for the second criterion of the prominence of the miRNA-guided target cleavage. The third and final parameter evaluated the pattern of cleavage, specifically, whether the abundance of the PARE sequence at the cleavage site was a high ‘peak’ above other degradation intermediates along the mRNA or was of similar abundance as the other PARE sequences from that RNA (Figure 5c). The peak percentage value was calculated as 100 times the sum of the top two PARE sequences from the target RNA, divided by the sum of all PARE abundances from that RNA. A value for ‘background’ degradation was first calculated from all annotated mRNAs in the WT library with PARE data. About 50% of all WT annotated transcripts had a peak percentage of less than 10%. For the validated miRNA targets with PARE cleavages, a minimum cut-off value of twice background, or a peak percentage of at least 20%, captured 61% to 74% of the known miRNA target cleavages in the WT and *xrn4* backgrounds, respectively, and was therefore chosen as the cut-off criterion for this parameter.

Using these three criteria, a pipeline was established to evaluate predicted miRNA targets that have PARE sequence data matching the precise predicted cleavage site, and to quantify the number of prominence criteria each fulfilled (Figure 5d). Targets meeting none of the criteria are designated Level 1, and targets meeting all the prominence criteria are designated Level 4. When the 137 previously validated *Arabidopsis* miRNA targets were evaluated with these criteria, 110 and 115 targets were identified (Levels 1 to 4) for WT and *xrn4*, respectively (Figure 5e). Although the number of known targets detected at any level for WT and *xrn4* was similar, the number of Level 4 targets was greater (63 versus 49) and the number of Level 1 targets was fewer (13 versus 21) for *xrn4*, consistent with the increased accumulation of miRNA cleavage products in the mutant [44,64]. All of the *Arabidopsis* miRNA targets examined were previously experimentally validated and most fulfill multiple criteria. However, it is apparent that all levels contain bona fide *Arabidopsis* miRNA targets (Figure 5e).

### PARE data support precise cleavage guided by most *Brachypodium* microRNAs, with higher prominence levels evident among annotated conserved microRNAs

**Figure 6:**
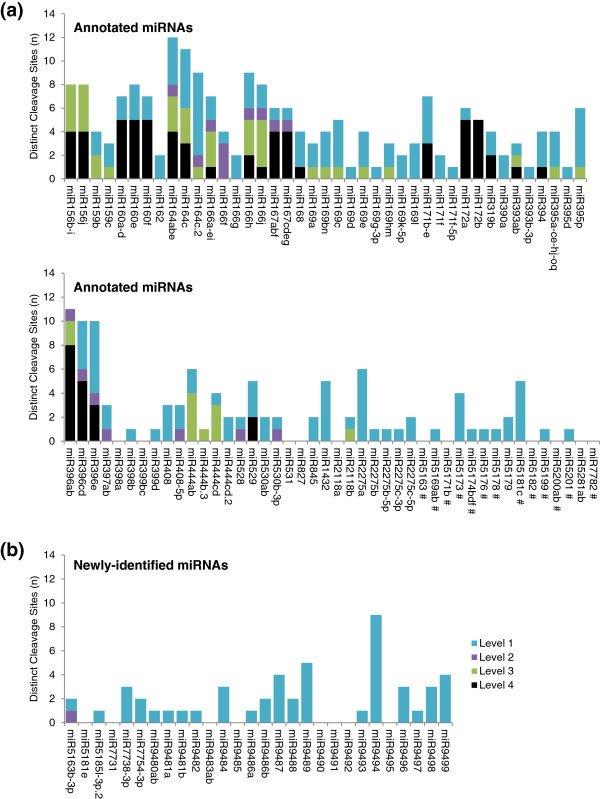
**Target prominence level distribution among precise cleavage sites with PARE data.** The number of distinct cleavage sites of each of the levels for **(a)** annotated miRNAs and **(b)** newly identified miRNAs are shown. miRNAs are listed by families in ascending order as in the supplemental tables. Non-conserved miRNAs are indicated with # in (a). miRNA, microRNA; PARE, parallel analysis of RNA ends.

Next, we sought to use the same pipeline outlined in Figure 5d to further characterize the precise target cleavage sites detected with PARE for *Brachypodium*. Specifically, 262 sites in 246 predicted miRNA targets from Table 4 that had PARE data indicating precise target cleavage were examined. The level designation for each and the individual criteria passed are listed in Additional file 1: Table S3. Data for the complete set of previously annotated and newly identified miRNAs from Figure 1 are compiled in Figure 6. Even without the benefit of an *xrn4* mutant, which is not available for *Brachypodium*, a high proportion (81%) of the 116 *Brachypodium* miRNAs had PARE sequences at the precise site(s) predicted for target cleavage. Among annotated miRNAs, 85% had PARE cleavage support whereas 70% did among those newly identified in this study. When the individual cleavage sites for the multiple targets predicted for each miRNA are considered, 42 sites were Level 4 targets, and 38 were level 2 or 3. The Level 1 cleavage sites predominated for the *Brachypodium* miRNA targets with precise PARE data, particularly those of the newly identified miRNAs. Level 1 did not predominate when PARE levels were compared for the *Arabidopsis* target sites used to establish the cut-offs (Figure 5e). However, the target cleavage sites examined for *Arabidopsis* were biased toward those that were easier to detect because only those that had prior experimental validation were examined by PARE.

**Figure 7:**
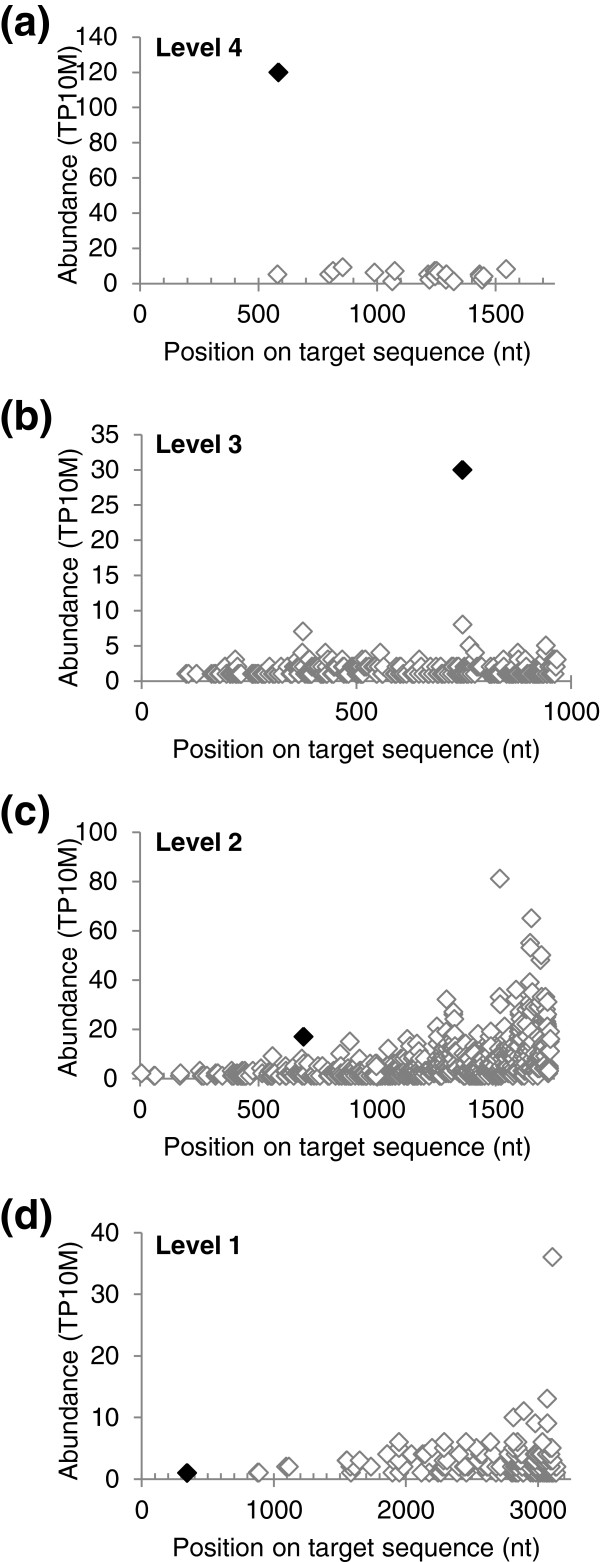
***Brachypodium *PARE data demonstrate miRNA-guided target cleavage.** Examples of degradation plots for miRNA targets for each prominence level. The abundance of all the PARE sequences matching miRNA targets are plotted with blank diamonds. The PARE sequence corresponding to the miRNA-mediated cleavage is represented by a black diamond. **(a)***Bradi1g52240*, encoding a GIBBERELLIC-ACID INSENSITIVE (GAI), REPRESSOR of GAI (RGA), and SCARECROW (SCR) (GRAS) transcription factor, is a Level 4 target of Bdi-miR171bcef. **(b)***Bradi3g05510*, encoding a SQUAMOSA PROMOTER BINDING PROTEIN-LIKE (SPL) transcription factor is a Level 3 target of Bdi-miR156b-ij. **(c)***Bradi4g39330*, encoding a laccase, is a Level 2 target of Bdi-miR397ac. **(d)***Bradi3g51077*, encoding argonaute 1a, is a Level 1 target of Bdi-miR168. nt, nucleotide; miRNA, microRNA; PARE, parallel analysis of RNA ends; TP10M, transcripts per 10 million.

An important observation made above for the level designations of *Arabidopsis* miRNA targets was also observed for those of *Brachypodium*; all prominence levels contain valid miRNA targets. For *Brachypodium*, this is indicated by the examples in the degradation plots (D-plots) in Figure 7, which indicate the location and abundance of the PARE sequence at the precise cleavage site as well as all PARE sequences matching these target RNAs. Although the PARE sequences at the Level 1 and Level 2 miRNA cleavage sites are not as prominent as those in the Level 3 and Level 4 examples, they are homologous to experimentally validated targets of conserved miRNAs in other plant systems. Additional D-plots for Level 1 to 4 targets are shown in Additional file 2: Figure S3. These data indicate that the level numbers represent a prominence score that is useful for characterizing miRNA target cleavage, and even a Level 1 cleavage at the precise predicted site for a miRNA should be considered quite strongly as evidence for cleavage.

### Analysis of PARE data for identifying microRNA cleavage-initiating phased small RNAs

A small group of miRNAs initiate the production of phased siRNAs from their target loci. The cleavage guided by the miRNA defines the beginning of the phase register, which can have a 21- or 24-nucleotide periodicity. Examples of miRNAs initiating 21-nucleotide phased loci are miR173 [19], miR390 [25] and miR2118 [30], whereas miR2275 [30] is the only miRNA currently known that initiates 24-nucleotide phased loci. It has previously been reported that *Brachypodium* contains hundreds of 21- and 24-nucleotide phased loci in its panicles [31]. Since *Brachypodium* produces miR390, miR2118 and miR2275, all known for initiating phased loci, these miRNAs may initiate some or all of these previously reported loci.

PARE data captures the downstream products of miRNA-mediated cleavage and thus could help identify the location of the cleavages initiating these phased loci and the identity of the miRNAs responsible. Using the same method as we used previously [31], over 1,100 phased loci were identified in leaf, panicle, stem and root small RNA libraries (as detailed in Materials and methods). The PARE sequences corresponding to each locus +/-5 phase cycles (105- or 120-nucleotide for 21- or 24-nucleotide periods, respectively) were collected. The 13 nucleotides upstream of the PARE sequence start was added to the first 11 nucleotides of the PARE sequence to create the miRNA-complementary site ±1. These miRNA-complementary sites were compared to Bdi-miR390, Bdi-miR2118 and Bdi-miR2275, and scored such that 1 was added for each mismatch and 0.5 was added for each G:U pair. Duplexes with a score of 5 or less were considered targets of the respective miRNA. PARE sequences were associated with 945 phased loci. The miRNAs initiating 437 of the phased loci were identified (Table 5). For all but six of the loci, the PARE sequence generated by that respective miRNA was within ±1 of the dominant phase register. An example of the PARE and small RNA sequence distribution from representative loci initiated by miR2118 is shown in Additional file 2: Figure S4. Identifying the miRNAs initiating approximately 500 phased loci showed that PARE data can be a valuable tool for analysis of these loci and their cleavage sites.

**Table 5:** PARE data identifies miRNA-initiated phased loci

**miRNA**	**Phased loci**^**a**^
miR390	2
miR2118a/b	288
miR2275	146
Bdi-miR5163b-3p	1

^a^Loci with a minimum phasing score of 25.

PARE, parallel analysis of RNA ends.

**Figure 8:**
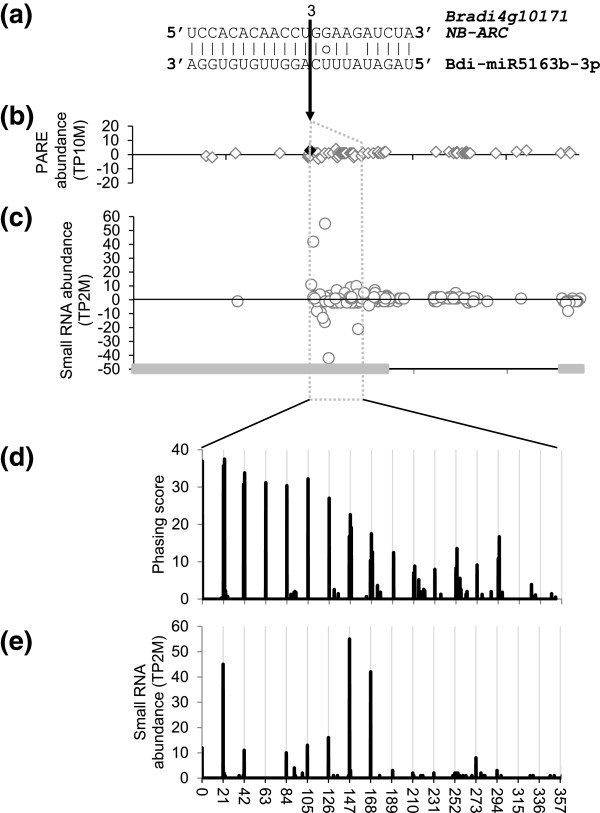
**Evidence that Bdi-miR5163b-3p**-**guided cleavage induces phased siRNAs from an NB-ARC mRNA. (a)** The sequence alignment of the *Bradi4g10171*, an *NB-ARC* gene, and Bdi-Bdi-miR5163b-3p. Lines indicate a perfect match. The circle indicates a G:U pair. The black arrow indicates the cleavage site with the TP10M PARE abundance listed above. **(b,c)** PARE and small RNA distributions for *Bradi4g10171*. The region shown is 9771971 to 9776753 on chromosome 4 and the gray boxes on the *x*-axis are the exons. Gray diamonds and circles represent PARE and small RNA sequences, respectively. The filled black diamond at the position indicated by the arrow is the PARE site generated by Bdi-miR5163b-3p cleavage. **(d)** The phasing score profile for the phased region. **(e)** The small RNA abundance profile for the phased region. PARE, parallel analysis of RNA ends; siRNA, small interfering RNA; TP10M, transcripts per 10 million; TP2M, transcripts per 2 million.

Only about half of the phased loci were accounted for by miR390, miR2118 and miR2275. Since it has been reported that 22-nucleotide miRNAs can potentially initiate phased loci [27,28], the remaining seven 22-nucleotide miRNAs from Additional file 1: Table S2 were examined for their potential to target the phased loci. PARE data indicates that Bdi-miR5163b-3p targets one locus, *Bradi4g10171*, encoding an NB-ARC (nucleotide binding human APAF-1, plant R protein, *Caenorhabditis elegans* CED-4) domain-containing R protein (Table 5, Figure 8). Phasing at this locus was found in the leaf, panicle, stem and root. Figure 8a shows the complementarity between Bdi-miR5163b-3p and *Bradi4g10171*. The PARE and small RNA were not evenly distributed across the gene, but rather concentrated after the PARE sequence indicative of Bdi-miR5163b-3p-mediated cleavage (Figure 8b,c). The position with the maximum phase score coincides with the exact position of Bdi-miR5163b-3p cleavage (Figure 8d), and nearly all of the small RNAs within the phased region precisely coincide with the phase register (Figure 8e). Taken together, this shows that Bdi-miR5163b-3p is a novel miRNA that initiates phasing of its target locus.

It has been proposed that phasiRNAs are often generated from protein-coding loci by miRNA-triggered cleavage and function to suppress gene families, such as those of the *PPR*, *NB-LRR* and *MYB* genes in *Arabidopsis*, *Medicago*, apple, and peach [21,23,65-67]. The aforementioned NB-ARC target of Bdi-miR5163b-3p is a member of the *NB-LRR* family of disease resistance genes as are several other predicted *NB-ARC* targets of this miRNA. Although we were not able to obtain cleavage evidence for the others from PARE data, all the *NB-ARC* targets of Bdi-miR5163b-3p are phasiRNA loci. It is notable that Bdi-miR5163b-3p does not share sequence similarity with miR472/482, miR2118, miR1507 and miR2109/miR5213, which are known to target NB domains of *NB-LRR* and trigger phasiRNA generation [21,22,24,67]. In addition, Bdi-miR5163b-3p targets the LRR domain instead of the NB domain. Bdi-miR5163b-3p has not been identified in other plants yet. These results imply that Bdi-miR5163b-3p may be a *Brachypodium*-specific miRNA that regulates *NB-ARC* genes and plays a role in disease resistance.

### PARE identifies differential cleavage by microRNA family members

**Figure 9:**
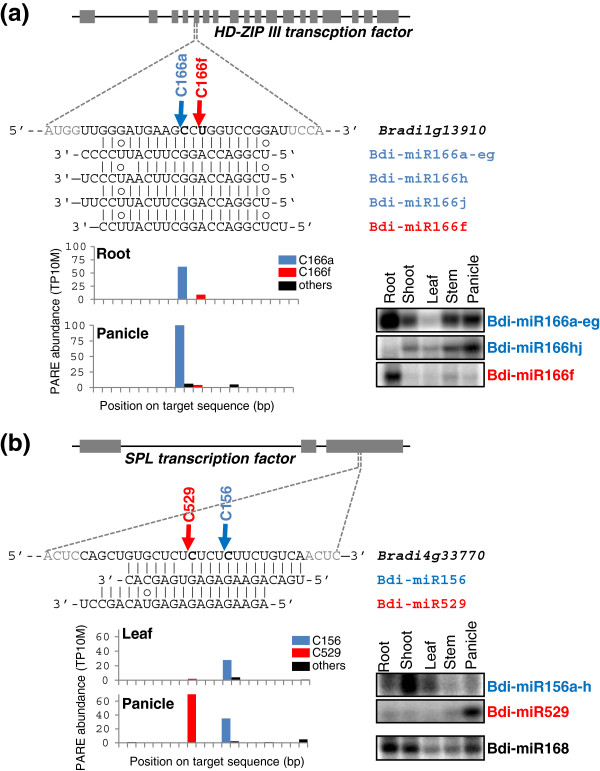
**miRNA family members show different patterns of tissue-preferential expression and target cleavage. (a)** miR166 family-mediated cleavage of *Bradi1g13910*, which encodes an HD-ZIP III transcription factor. **(b)** miR156 family-mediated cleavage of *Bradi4g33770*, which encodes a SPL transcription factor. The schematic diagrams of genomic structures for miRNA target genes are shown. The gray boxes indicate exons. The sequences of miRNA target sites and miRNA family members were aligned. Lines indicate a perfect match. Circles indicate G:U pairs. PARE sequence distribution for target sites are shown with histograms. The expression patterns of miRNA family members were examined for diverse tissues using the splinted ligation-mediated miRNA detection method. Bdi-miR168 was used for the control. bp, base pair; HD-ZIP, homeobox domain-leucine zipper; SPL, Squamosa Promoter Binding Protein-Like; miRNA, microRNA; PARE, parallel analysis of RNA ends; TP10M, transcripts per 10 million.

Many miRNA family members have slight differences in sequences and differential expression patterns, which could cause their specificity for target regulation [34]. We investigated differential regulation of targets by miRNA family members using PARE data. We observed that two miRNA families, miR166 and miR156/529, have members with both sequence variation and differential regulation patterns. In *Brachypodium*, ten *MIR* genes encode miR166. While six *MIR166* genes generate identical guide sequences, *MIR166h*, *MIR166j* and *MIR166f* give rise to miRNA variants with slight sequence variations. Interestingly, the three miR166 sequences showed differential expression patterns in diverse tissues (Figure 9a). The most abundant sequence, miR166a-eg, was ubiquitously expressed except in mature leaf tissue of six-week-old plants. However, miR166f and miR166h exhibited tissue-preferential expression in roots and panicles, respectively. The expression pattern of miR166j was not distinguishable due to sequence similarity with miR166h. Given the clustered structure of *MIR166h* and *MIR166j* (Figure 3c), it is likely that miR166j expression is similar to miR166h. To address whether the differentially expressed miR166 family members also differentially regulate their target genes, we examined the cleavage events in a target gene encoding a homeobox domain-leucine zipper (HD-ZIP) transcription factor. As shown in Figure 9a, the target gene has two major PARE sequences corresponding to cleavage sites by miR166a-eg, miR166h, miR166j and miR166f. Since miR166f has a 5′-end two-nucleotide offset relative to the other miR166 family members, the miR166f-mediated cleavage site is also shifted relative to the cleavage site mediated by other family members. In root tissue, two different PARE sequences were mapped to the cleavage sites of both the root-preferential miR166f and the remaining miR166 family members. However, the panicle library showed only one prominent cleavage site by miR166a-eg, miR166h and miR166j, while miR166f-mediated target cleavage was detected at only a basal level. This implies that miR166 members may regulate *HD-ZIP* gene expression in a tissue-preferential manner.

It has been reported that the regulation of *OsSPL14* expression by Osa-miR156 and Osa-miR529 may play a crucial role in both tillering and panicle-branching in rice [34,68,69]. To explore this regulation in *Brachypodium*, we first examined the expression pattern of Bdi-miR156 and Bdi-miR529. Bdi-miR156 was highly expressed in young shoot tissues, and downregulated in mature leaf tissues (Figure 9b). In addition, Bdi-miR156 expression in panicle tissue was much lower than that in the other tissues, whereas Bdi-miR529 was preferentially expressed in panicle tissue. These expression patterns are similar to those in rice [34,70]. Next, we addressed the effect of these two miRNAs on a target gene encoding BdiSPL14L (*Brachypodium distachyon* Squamosa Promoter Binding Protein 14-Like, an OsSPL14 homolog in *Brachypodium*), using PARE data. In leaf tissue of three-week-old plants, a prominent PARE sequence was mapped to a cleavage site by miR156, while the PARE sequence from the site of miR529-mediated cleavage had a very low abundance. In contrast, the PARE sequence corresponding to the site of cleavage mediated by panicle-preferential miR529 was more abundant than that of miR156 in the panicle library. This result implies that cleavage of *BdiSPL14L* during vegetative growth is predominantly miR156-guided, whereas miR529 and miR156 regulate the same gene during reproductive development. In conclusion, the two examples of miRNA families shown to be differentially expressed and to guide differential target cleavage highlight the potential importance, in general, of distinct miRNA family members for post-transcriptional control.

## Discussion

In this study, we have provided a better understanding of *Brachypodium* miRNAs in several ways. The deep sequencing of 17 small RNA libraries as well as four PARE libraries from various tissues and environmentally stress-treated plants allowed for the identification of many new miRNAs and the characterization of their roles in target cleavage. The miRNAs identified include both conserved miRNAs that have not been reported in *Brachypodium*, as well as non-conserved miRNAs that have not been found in other plants and thus Thus, these miRNAs may function in gene regulatory mechanisms specific to *Brachypodium* or those shared with related species*.* Our analysis uncovered a total of 81 new miRNA precursors of conserved and non-conserved miRNAs. These confirmed 11 conserved and five non-conserved miRNA precursors that were recently reported [42]. In addition, evidence that miR162-mediated *DCL1* regulation may not function in *Brachypodium* was presented. We also identified a new mode of miR5200 regulation in response to submergence stress. More than 260 targets of new and known miRNAs with PARE sequences at the precise cleavage site were identified and characterized with a set of prominence criteria. Validated miRNA targets were found at all prominence levels and even low prominence was found to be characteristic of some targets. Combining PARE data with the small RNA data identified the miRNAs responsible for initiating approximately 500 phased loci, including one of the novel miRNAs, Bdi-miR5163b-3p. PARE data also supported differential target cleavage in various tissues by miRNA variants.

### Null function of miR162-mediated *DCL1* regulation in *Brachypodium*

It has been proposed that miR162-mediated negative feedback regulation of *DCL1*, encoding a protein responsible for miRNA biogenesis, is a widely conserved mechanism in flowering plants [71,72]. In *Arabidopsis*, *DCL1* is targeted not only by miR162, encoded at two distinct loci, but also by miR838, generated from a stem-loop in intron 14 of the *DCL1* pre-mRNA [73]. Both miRNAs repress *DCL1* mRNA expression, while DCL1 is required to process those miRNAs. This negative feedback mechanism may be required for a sophisticated homeostatic regulation of miRNA processing and subsequent target regulation in flowering plants. Even though miR838 seems to be an *Arabidopsis*-specific miRNA, miR162 is widely conserved and identified in most flowering plant species, including *Arabidopsis*, poplar, *Medicago*, tomato, rice, maize and others. However, miR162 expression was found to be extremely low in wheat [39,74], barley [75] and sorghum [76]. Our data, as well as a recent publication [39] indicate extremely low expression of miR162 in *Brachypodium* despite the presence of a miR162 precursor. Among the 17 libraries reported here, miR162 was only sequenced once and was beneath detection when examined by RNA blot (Figure 2). We also could not find any PARE sequence corresponding to a Bdi-miR162 target site in a *Brachypodium* gene that is homologous to *DCL1* (*Bradi1g77087.1*).

We identified a one-nucleotide difference in miR162 compared to the conserved sequence and hypothesized that this change may affect the stability of miRNA:miRNA* pairing, resulting in inefficient processing of the duplex. We cannot rule out other possibilities like reduced stability, less efficient nuclear export or inefficient AGO1 loading of the miRNA:miRNA* duplex. However, the lack of PARE sequences corresponding to the *MIR162* locus supports the inefficient processing of the precursor by DCL1. We also cannot rule out the possibility that the *MIR162* promoter is not active in the tissues analyzed in *Brachypodium* and its relatives. It has been reported that altered miRNA:miRNA* base pairing can affect miRNA processing in viruses, animals and plants [77,78]. In animals, those mutations are associated with cancer and other diseases [79]. In *Arabidopsis*, it has been reported that a natural variation in miR164a* from the C24 ecotype affects efficient processing of miR164a, resulting in altered leaf serration and an accessory bud formation phenotype in the C24 ecotype [80]. The functional effect of low expression of miR162 in *Brachypodium* is still unclear. In the moss *Physcomitrella patens*, Ppt-miR1047, embedded in intron 7 of *PpDCL1a*, targets a host gene, *PpDCL1* mRNA [72]. Despite the sequence and position differences between Ath-miR838 and Ppt-miR1047, they appear to have analogous functions in the negative feedback regulation of *DCL1* pre-mRNAs. This finding suggests that the miRNA-*DCL1* feedback loop is critical for control of miRNA function in land plants. Therefore, it is likely that *Brachypodium* and its relatives may have some other mechanism to regulate *DCL1* gene expression, or have evolved to control DCL1 function without a miRNA-*DCL1* feedback loop.

### miR5200 regulates gene expression of a flowering time regulator under submergence conditions

We have identified a submergence-inducible miR5200 that is predicted to target two genes encoding *FT*-*like* genes in *Brachypodium*. Interestingly, *Bdi-FTL1* was downregulated during submergence when miR5200 is induced (Figure 4b). It has been proposed that flowering is inhibited by submergence both in *Arabidopsis* and rice [81]. Ectopic expression of the rice *SUBMERGENCE1A* (*SUB1A*) gene in *Arabidopsis* led to flowering inhibition by reducing the level of *CONSTANS* (*CO*) and *FT* mRNAs. In addition, ectopic over-expression or submergence-induced expression of *SUB1A* in rice affected the abundance of mRNAs encoding *Hd1* and *Hd3a*, which encode two conserved flowering regulators of *CO* and *FT*, respectively. It is possible that *Brachypodium* also contains a similar regulatory mechanism where submergence represses flowering time by downregulation of *Bdi-FTL1*. In addition, miR5200 may also play a crucial role in downregulation of *Bdi-FTL1.* However, miR5200 was not identified in either rice or *Arabidopsis*. Our miRNA conservation analysis (Additional file 2: Figure S5) showed that wheat and barley express miR5200 or related small RNAs at low levels. Thus, miR5200 in wheat and barley also could be upregulated under submergence and target *FT-*like genes.

### Known and potential outcomes of modulating miR156/529 expression

Recently, it has been shown that over-expression of miR156 in rice, maize, switchgrass and *Brachypodium* caused morphological alterations including an increased number of branches, resulting in a favorable phenotype for improved biomass production [70,82-84]. However, over-expression of miR156 can also result in other defects like dwarfism and male sterility, likely because miR156 represses many *SPL* genes. Increased expression of *OsSPL14* due to sequence alteration of the miR156 target site leads to less tillers and more panicle branching, resulting in increased grain yields in rice [68,69]. Additionally, miR529, which is in the same miRNA family as miR156, has been proposed to play a crucial role in panicle branching [34]. Sequence variations and differential expression patterns between miR156 and miR529 could cause their distinct function in *SPL* gene repression. In our data, Bdi-miR156 and Bdi-miR529 also showed a similar differential regulation of their target gene, *Bdi-SPL14L*, when they were expressed (Figure 9b). In contrast to miR156, the effects of over-expressing miR529 have not been examined yet for transgenic plants. Since Bdi-miR529 and Bdi-miR156 share several target genes and may differ for others, their over-expression should be significant and, depending on the outcome, may suggest strategies to improve food or bioenergy crops.

### Power of PARE to identify cleaved targets of *Brachypodium* microRNAs

The PARE data we examined in this study represents a major advance because, prior to this work, the cleavage of miRNA targets had only been demonstrated by examining a limited set of *Brachypodium* miRNA targets [39,53]. The distinct cleavage of *Bdi-SPL14L* guided by Bdi-miR156 and Bdi-miR529 as discussed above, was precisely pinpointed at sites four nucleotides apart using PARE (Figure 9b), reminiscent of similar observations from rice PARE data [34]. Cleavage guided by Bdi-miR166 family members in an *HD-ZIP III* mRNA at sites two nucleotides apart was also validated with PARE (Figure 9a). In total, PARE evidence for precise cleavage at more than 263 miRNA cleavage sites was obtained. The targets with PARE cleavage evidence corresponded to 70% of the new miRNAs and 85% of the annotated miRNAs.

We found that using two distinct target prediction programs, CleaveLand and psRNATarget, was a more powerful way to predict more miRNA targets for cleavage validation with PARE than either algorithm alone (Additional file 2: Figure S2). Accordingly, this larger pool enhanced the number of cleaved targets precisely identified with PARE. We developed a characterization tool to examine the prominence of the PARE sequence at the precise cleavage site of these targets using criteria established based on *Arabidopsis* miRNA targets with independent experimental validation. This tool proved useful in several ways. First, it identified the PARE-validated targets that had the most prominent target cleavage data as exemplified by the D-plots for Level 3 or 4 targets (Figure 7 and Additional file 2: Figure S3). Second, since experimentally confirmed miRNA targets are present at all levels, by knowing which criteria a particular Level 2 or 3 target has passed (as listed in Additional file 1: Table S3), potential explanations and future experiments can be identified. For instance, a target with a D-plot exhibiting many decay intermediates towards the 3′ end or throughout the RNA may explain why the precise target may not be in the top two most abundant PARE sequences or pass the peak percentage criteria. Alternatively, the PARE sequence at the cleavage site may be of low abundance. This could be due to low accumulation of the miRNA or the target RNA in the samples examined, or because the miRNA largely inhibits translation and cleavage is less prominent. Cleavage can be detected for translationally regulated miRNA targets as is the case for miR172 and miR398 targets [16,85]. Moreover, for some targets, a low prominence level may be a conserved characteristic of the target RNA. This is clearly the case for *AGO1*. Cleavage of *AGO1* mRNA is guided by miR168 and AGO1 is a Level 2 target in *Arabidopsis* and a Level 1 target in rice and *Brachypodium* (Figure 7d and Additional file 2: Figure S6). As PARE libraries are sequenced from diverse samples and closely related species, some of the *Brachypodium* targets of new or annotated miRNAs may be classified in higher levels or found to be characteristically Level 1. The PARE data contributed by this work is extensive but likely will be augmented with additional *Brachypodium* PARE data in the future.

## Conclusions

The knowledge gained from the identification of new miRNAs and cleavage-validated miRNA targets enhances our understanding of regulatory pathways in *Brachypodium* and should inform similar studies in related plants. The novel regulation of miRNA expression detected under submergence conditions is one example. Another is the differential regulation of closely related miRNA family members, which correlates with differential target cleavage detected with PARE. The PARE data contributed by this study also contains RNA decay intermediates derived from mechanisms independent of miRNAs and thus should prove useful for studying other components of the RNA degradome.

## Materials and methods

### Plant growth and stress treatment

*Brachypodium* (Line Bd21) was used for all libraries. For the BDI small RNA and PARE libraries, seeds were germinated in soil and the plants were grown in a growth chamber at a constant 20°C, under a 20 h light, 4 h dark cycle. Leaf, stem and panicle tissues were harvested from six-week-old plants. Leaf tissue was taken from the top of the blades of the first and second leaves, not including the leaf sheath. Stem tissue was harvested from just above the first top node to just below the second node, including both nodes as well as the leaf sheath of the second leaf. Panicle tissue was harvested from plants with immature, unfertilized flowers from the tip of the spikelet to just above, but not including, the panicle node at approximately 10 h into the subjective day. To collect root tissue, seeds were husked, surface-sterilized with a 50% bleach solution, rinsed with sterile water and germinated on half-strength Murashige and Skoog (MS) medium containing 0.2% (w/v) Phytagel in sterile plastic boxes. Root tissue was harvested from three-week-old plants from the tip of the root to just below, but not including the seed.

For the OBD set of small RNA libraries from tissues, *Brachypodium* plants were grown with supplemental light for a 16 h photoperiod at 25°C. For OBD02, leaf and stem tissues were collected at days 7 to 8, 14 to 15, and 27 to 28 and pooled. Panicle tissue was collected at days 28 to 35 at four times: 0700 (dawn), 1300, 1900 and 0100 hours. For OBD03, whole seedlings including roots were pooled. Two samples were grown in soil under a 16 h photoperiod and tissues were collected from three- and four-day-old plants. Two additional samples included five-day-old etiolated seedlings and light-grown seedlings grown on moistened filter paper.

Stress treatments and controls except phosphate starvation and its control for BDI small RNA libraries were conducted using three-week-old plants planted in soil under normal growth conditions with a 20 h photoperiod as above. Stresses began 5 h to 6 h after the lights came on. For heat stress, plants were moved to 40°C for 12 h. For cold stress, plants were moved to 4°C in a cold room for 12 h. For drought stress, plants were removed from the soil and rinsed, blotted dry with paper towels, and air-dried for 12 h. For salt stress, the base of the plant pot was submerged in 300 mM NaCl solution for 12 h to a depth of approximately 1 inch, without covering the soil. For submergence stress, plants were fully submerged in 20°C water for 24 h. For phosphate starvation stress, seeds were planted in MS media without KH_2_PO_4_, and control plants were planted in MS media; plants were harvested at three weeks, 5 to 6 h after lights on. Control plants were sampled at the same time as the stress treatments.

For OBD04, tissues from plants treated with abiotic, biotic and mechanical stresses were pooled. For abiotic stress, leaves and stems were collected from plants subjected to heat (37°C), cold (4°C), salt (200 mM NaCl) and osmotic (300 mM mannitol) treatments. For biotic stress, leaves were collected from plants with the following treatments. For the bacterial treatment, *Pseudomonas fluorescens* and *Pseudomonas syringae* pv. *tomato* DC3000 (*Pto* DC3000) were delivered to 28–29-day-old *Brachypodium* leaves under vacuum at 1 × 10^7^ cfu/mL. Leaves of four-week-old plants were placed in a 1.5 ml microcentrifuge tube containing 20 μg/mL victorin. Tissue was harvested 24 h after treatment with victorin. Mechanical stress was applied by crushing the leaves of 29–30-day-old plants with tweezers. Unless otherwise indicated, libraries were made from a pool of samples collected every 4 h after the stress began, that is, 4 h + 8 h + 12 h + 16 h + 20 h + 24 h.

All collected tissues were immediately frozen in liquid nitrogen and stored at -80°C until RNA extraction.

### Small RNA library construction and sequencing

The BDI set of small RNA libraries were constructed as described [31,86] with minor modifications. Briefly, total RNAs were isolated using the Trizol reagent (Molecular Research Center), and low molecular weight RNA was purified from total RNA by PEG8000/NaCl precipitation. Following denaturing urea PAGE purification of the 20- to 30-nucleotide components, small RNAs were ligated first with the 5′ RNA adapter (5′ GUUCAGAGUUCUACAGUCCGACGAUC 3′) and then the 3′ RNA adapter (5′ PUCGUAUGCCGUCUUCUGCUUG-idT 3′). In each step, the ligated products were purified with PAGE gel. After the first strands of cDNAs were synthesized using RT primer (5′ CAAGCAGAAGACGGCATACGA 3′) and purified with PAGE gel, the final bands were amplified using 18 cycles of PCR using PCR primers (forward PCR primer: 5′ AATGATACGGCGACCACCGACAGGTTCAGAGTTCTACAGTCCGA 3′, reverse PCR primer: 5′ CAAGCAGAAGACGGCATACGA 3′) and purified with PAGE gel for sequencing. The OBD set of small RNA libraries was constructed as described [31]. Libraries were sequenced on an Illumina GAIIx instrument.

### Computational analysis of small RNA sequencing data and microRNA identification

The raw sequencing data was trimmed by removing adapter sequences, and mapped to the *Brachypodium* genome 8X assembly [87] using Bowtie [88]. The two pipelines used for the computational analysis of the small RNA libraries and the identification of miRNAs is outlined in Figure 1. For the stringent criteria pipeline, small RNA sequences 20 to 24 nucleotides in length, with an abundance of ≥10 TP2M in at least one library, and ≤20 total genome hits, were evaluated for potential pairing of miRNA and miR* using a modified version of miREAP optimized for plant miRNAs [21,34]. Strand bias was calculated as the total abundance of sequences matching the sense strand divided by the total abundance of all sequences matching both strands. Abundance bias was calculated as the sum of the abundances of the top two sequences for the precursor divided by the total abundance of all matching sequences. Sequences with a strand bias of ≥0.9 and an abundance bias of ≥0.7 were selected and the stem-loop structure was predicted using UNAFold [89]. Precursor sequences with ≤4 mismatches and bulges in the miRNA:miRNA* pairing, and a bulge size of ≤1 nucleotide were selected for final manual analysis. Each precursor structure was inspected manually to give visual confirmation of the secondary structure, that the predicted miRNA sequence was the most abundant sequence variant at the precursor and that additional miRNA sequences originate from the same precursor. A custom Perl script for this pipeline is available as Supplemental Dataset 1 in Jeong *et al*. [34].

For the sequence homology pipeline, annotated miRNAs from 47 flowering plant species in miRBase Release 18 were mapped to the *Brachypodium* genome 8X using Bowtie [88] with zero mismatches. miRBase sequences 20 to 24 nucleotides in size with ≤20 genome hits were then matched to the small RNA libraries to identify conserved miRNAs. Further conserved candidates were selected from the small RNA sequences that had an abundance ≥1 TP2M in at least one library and did not pass the stringent criteria pipeline. If multiple sequences mapped to the same genome position, the most abundant sequence was chosen. A putative precursor was selected from sequences 150-nucleotide upstream and 150-nucleotide downstream from the 5′ end of the miRNA. For sequences within 100 nucleotides of each other, the most abundant miRNA was selected for choosing the precursor location. Stem-loop structures of the putative precursors were predicted using UNAFold [89], and precursor sizes and positions were adjusted manually to achieve the best folding structure. Sequences with ≤8 mismatches plus bulges within the miRNA/miRNA* pairing were then evaluated for strand bias and abundance bias with cut-offs of 0.9 and 0.4, respectively. Finally, precursors were evaluated manually for additional miRNA candidates.

Identified miRNAs were matched to miRBase Release 18 with up to two mismatches allowed for naming. Sequences that did not match any known miRNA were considered novel; those sequences only found in *Brachypodium* were considered non-conserved and annotated, and those sequences matching at least one additional species were considered conserved miRNAs.

Additionally, miRNAs with known family members that did not pass any of the pipelines were manually searched for in miRBase Release 19 using the criteria of up to three mismatches.

### microRNA detection using RNA blots

Low molecular weight RNA was isolated from 150 μg of total RNA by PEG/NaCl precipitation and separated on 15% denaturing PAGE gels, transferred to Hybond N^+^ nylon membranes (Amersham Biosciences), and fixed by UV cross-linking. The DNA oligonucleotide probes listed in Additional file 1: Table S4 were purified and hybridized [34].

### microRNA RNA detection with splinted ligation-mediated miRNA detection

miRNAs were detected using the miRNAtect-It miRNA labeling and detection kit (Affymetrix) as in [90-92]. Specific bridge oligonucleotides shown in Additional file 1: Table S5 were ligated to both 5 to 20 μg of total RNA and a P^32^-labeled detection oligo. Products were separated on a 15% denaturing PAGE gel and visualized using a Typhoon Phosphoimager.

### mRNA detection using RNA blots

To detect mRNA, 20 μg of total RNA was loaded on 1.5% formaldehyde agarose gels, transferred to Hybond N^+^ nylon membranes, and probed with P^32^-labeled complementary DNA sequences generated by PCR using the primers listed in Additional file 1: Table S5 and the Church and Gilbert hybridization buffer [93].

### PARE library construction and sequencing

PARE libraries were constructed as described [94] with minor modifications. Briefly, total RNA was extracted with Trizol Reagent (Molecular Research Center) and PolyA RNA was isolated. A 5′ RNA adapter was added to ligation-competent RNA molecules containing a 5′ monophosphate, and the resulting products were reverse-transcribed into cDNA using an oligo(dT) primer with a 3′ adapter sequence, and amplified by PCR. In each step the ligated products were purified with PAGE gel. The resulting product was digested with *Mme*I to capture 20- or 21-bp fragments. *Mme*I cleavage products were ligated with a double-stranded DNA 3′ adapter with degenerate overhangs and amplified by PCR. Final bands were purified with PAGE gel and used for high-throughput sequencing. Libraries were sequenced on an Illumina GAIIx instrument.

### Identifying microRNA targets

Putative miRNA targets were identified using three target prediction schemes. Using the CleaveLand v2.0 suite of software [95], miRNAs were matched to *Brachypodium* coding sequence CDSv1.2 [96] using SHRiMP V2.2.0 [87], and miRNA targets with a target score ≤7 were identified using the Perl script described previously [97]. A second, modified version of CleaveLand (CleaveLandM) was run, again using SHRiMP V2.2.0, to match to the coding sequence, but modified to use the scoring system detailed in [98]. Targets were identified with a score ≤4. Finally, targets were predicted using psRNATarget [99] with the following parameters: the maximum expectation was 5.0, length for complementarity scoring (hspsize) was set to the length of the miRNA, and the range of central pairing was 10 to 11 [100].

### Characterizing microRNA targets in PARE libraries

PARE sequences were trimmed of adapters and down to 20 nucleotides. PARE sequences were then matched to the *Brachypodium* genome 8X assembly and CDS (MIPSV1.2) using Bowtie. Sequences matching the genome more than 20 times, as well as regions of single-, double- or triple-nucleotide sequences >12 nucleotides in length were removed. PARE libraries were normalized to TP10M by multiplying by a factor of 10,000,000 divided by the number of genome-matched sequences Using custom-written Perl scripts, predicted miRNA targets were matched to the PARE data from each of the four tissue libraries in Table 3, and PARE sequences that paired with the miRNA at the precise canonical cleavage site were identified. For each PARE sequence passing these filters, the data from the library in which the precise PARE sequence at the target cleavage site had the highest abundance was used for further characterization using the prominence criteria outlined in the text.

Previously published *Arabidopsis* inflorescence wild-type (Col-0) and *xrn4* mutant PARE libraries [44] were exactly matched to the TAIR9 genome and cDNAs. Sequences matching the genome more than 20 times, sequences matching rRNAs, tRNAs, small nuclear RNAs and small nucleolar RNAs, and sequences with single-, double- or triple-nucleotide repeats >12 nucleotides in length were removed. PARE parameters, including abundance, peak percentage and abundance rank in the transcript, were established for the 137 previously reported *Arabidopsis* miRNA targets listed in Additional file 1: Table S4, and criteria for the *Brachypodium* PARE libraries was established from parameters of the *Arabidopsis* miRNA targets. Abundance is the abundance of the PARE sequence at the exact miRNA cleavage site. The peak percentage was calculated as the abundance of the PARE sequence exactly matching the miRNA cleavage site divided by the sum of all PARE sequences matching the transcript times 100. The abundance rank was determined by listing all PARE sequences matching the transcript in order from largest to smallest.

### Phasing analysis

The 21- and 24-nucleotide phased loci were identified using Perl scripts as previously described [31] with a minimum phasing score ≥25. The small RNA libraries BDI02, BDI04, BDI05, BDI06, BDI08, OBD01, OBD02 and OBD03 were used for this analysis. To set the boundaries of the phased loci, the libraries were combined and the 5′ end was set to the position where the phase score first was ≥20 in any library. The 3′ boundary was set at the last phase window with a score ≥20. The PARE sequences within five phase periods of these loci were pulled from the BDI25 PARE library and scored for complementarity to Bdi-miR390, Bdi-miR2118, Bdi-2275 and the 22-nucleotide small RNAs listed in Additional file 1: Table S2. Each mismatch between the target and miRNA had a penalty of 1 and each G:U pairing had a penalty of 0.5. Those with a score of 5 or less were considered targets of the respective miRNA.

### Accession numbers

Newly generated small RNA data have been deposited at the National Center for Biotechnology Information Gene Expression Omnibus (NCBI GEO) under accession number GSE52441 (GSM1266839 to GSM1266857). Two panicle small RNA libraries were previously published [31] and registered under GEO numbers GSM506620 and GSM506621. The data are also available online [101,102].

## Abbreviations

AGO: ARGONAUTE protein; bp: base pair; CDS: coding sequence; DCL: Dicer-like protein; D-plot: degradation plot; kb: kilobase; HD-ZIP: homeobox domain-leucine zipper; LRR: leucine-rich repeat; miRNA: microRNA; MS medium: Murashige and Skoog medium; NB: nucleotide binding; PAGE: polyacrylamide gel electrophoresis; PARE: parallel analysis of RNA ends; PCR: polymerase chain reaction; phasiRNA: phased siRNA; RISC: RNA-induced silencing complex; RT: reverse transcriptase; siRNA: small interfering RNA; tasiRNA: trans-acting siRNA; TP10M: transcripts per 10 million; TP2M: transcripts per 2 million; WT: wild type.

## Competing interests

The authors declare that they have no competing interests.

## Authors’ contributions

D-HJ, SAS, SP, MG, MAG and MA performed the experiments. D-HJ, SAS, LAR, JZ, BCM and PJG analyzed the data. DFG provided materials. NF, SEF, TCM and JCC developed and provided data for the OBD libraries. D-HJ, SAS, LAR and PJG wrote the manuscript. All authors read and approved the final manuscript.

## Supplementary Material

Additional file 1: Table S1 Precursors of *Brachypodium* miRNAs. **Table S2.** Conserved and non-conserved annotated miRNAs. **Table S3.** miRNA targets with PARE sequences at the predicted cleavage sites. **Table S4.***Arabidopsis* miRNA targets. **Table S5.** Oligomers used in this study. Click here for file

Additional file 2: Figure S1 Predicted secondary structure of Bdi-MIR444 precursors. **Figure S2.** Target prediction programs identified distinct subsets of targets. **Figure S3.** Representative D-plots for miRNA targets at each stringency level. **Figure S4.** Bdi-miR2118 cleavage induces phasing. **Figure S5.** Bdi-miR5200 in diverse plant species. **Figure S6.** D-plots of AGO1 for *Arabidopsis*, rice and *Brachypodium.* Click here for file
