# Supplementary material for: Parallel analysis of RNA ends enhances global investigation of microRNAs and target RNAs of Brachypodium distachyon
Source: Genome Biol. 2013 Dec 24;14(12):R145. doi: 10.1186/gb-2013-14-12-r145 (PMC4053937; doi:10.1186/gb-2013-14-12-r145)
Supplement: Additional file 1: Table S1 — Precursors of Brachypodium miRNAs. Table S2. Conserved and non-conserved annotated miRNAs. Table S3. miRNA targets with PARE sequences at the predicted cleavage sites. Table S4.Arabidopsis miRNA targets. Table S5. Oligomers used in this study. [file gb-2013-14-12-r145-S1.docx]

**Table S1. Precursors of Brachypodium miRNAs.**

^1^miRNA gene families were grouped based on the sequence similarity of the mature miRNAs. Strand ratio is the ratio of the sum of TP2M values from the most abundant small RNAs, to the sum of all the small RNAs matching a miRNA precursor. The abundance ratio is the ratio of the sum of TP2M values of the small RNAs matching the same strand of a precursor, to the sum of TP2M values of the small RNAs matching both strands.

| Family | Precursor | Chr. | Strand | Start | End | Strand Ratio | Abundance Ratio | Pipeline | Classification | miRbase19 |  |
| --- | --- | --- | --- | --- | --- | --- | --- | --- | --- | --- | --- |
| 156/529 | Bdi-MIR156b | 3 | - | 39,258,133 | 39,258,291 | 1 | 0.99 | S | conserved | annotated^3^ |  |
|  | Bdi-MIR156c | 3 | - | 4,336,042 | 4,336,153 | 1 | 0.99 | S | conserved | annotated^3^ |  |
|  | Bdi-MIR156d | 5 | - | 18,202,193 | 18,202,324 | 1 | 0.99 | S | conserved | annotated^3^ |  |
|  | Bdi-MIR156e | 2 | + | 4,030,441 | 4,030,592 | 1 | 0.98 | S | conserved | newly identified |  |
|  | Bdi-MIR156f | 2 | + | 4,030,530 | 4,030,732 | 1 | 0.98 | S | conserved | newly identified |  |
|  | Bdi-MIR156g | 2 | + | 4,030,923 | 4,031,015 | 1 | 0.98 | S | conserved | newly identified |  |
|  | Bdi-MIR156h | 3 | - | 49,973,606 | 49,973,693 | 1 | 0.99 | S | conserved | newly identified |  |
|  | Bdi-MIR156i | 4 | + | 36,168,970 | 36,169,104 | 1 | 0.98 | S | conserved | newly identified |  |
|  | Bdi-MIR156j | 4 | - | 38,856,201 | 38,856,309 | 1 | 0.99 | S | conserved | newly identified |  |
|  | Bdi-MIR529 | 3 | + | 44,898,503 | 44,898,618 | 1 | 0.92 | S | conserved | annotated^2^ |  |
| 159/319 | Bdi-MIR159b | 2 | + | 1,091,343 | 1,091,535 | 1 | 0.89 | S | conserved | newly identified |  |
|  | Bdi-MIR159c | 1 | - | 302,390 | 302,552 | 1 | 0.98 | S | conserved | newly identified |  |
|  | Bdi-MIR319a | 4 | + | 31,253,311 | 31,253,546 | 0.99 | 0.86 | S | conserved | annotated^2,3^ |  |
|  | Bdi-MIR319b | 2 | - | 45,992,393 | 45,992,578 | 1 | 0.81 | S | conserved | annotated^2^ |  |
| 160 | Bdi-MIR160a | 1 | - | 4,550,728 | 4,550,853 | 1 | 0.92 | S | conserved | annotated^3^ |  |
|  | Bdi-MIR160b | 1 | - | 28,020,440 | 28,020,529 | 1 | 0.93 | S | conserved | annotated^3^ |  |
|  | Bdi-MIR160c | 3 | - | 3,414,603 | 3,414,697 | 1 | 0.94 | S | conserved | annotated^3^ |  |
|  | Bdi-MIR160d | 3 | + | 12,734,311 | 12,734,423 | 1 | 0.94 | S | conserved | annotated^3^ |  |
|  | Bdi-MIR160e | 3 | + | 41,554,298 | 41,554,416 | 1 | 0.98 | S | conserved | annotated^3^ |  |
|  | Bdi-MIR160f | 5 | - | 19,337,418 | 19,337,548 | 1 | 0.92 | S | conserved | newly identified |  |
| 162 | Bdi-MIR162 | 3 | + | 49,591,993 | 49,592,143 | 1 | 1 | H | conserved | annotated^2^ |  |
| 164 | Bdi-MIR164a | 2 | - | 19,949,340 | 19,949,500 | 0.99 | 0.94 | S | conserved | annotated^2,3^ |  |
|  | Bdi-MIR164b | 1 | + | 14,544,043 | 14,544,193 | 1 | 0.98 | S | conserved | annotated^3^ |  |
|  | Bdi-MIR164c | 3 | - | 59,107,108 | 59,107,181 | 1 | 0.69 | H | conserved | annotated^3^ |  |
|  | Bdi-MIR164e | 2 | - | 26,808,991 | 26,809,097 | 1 | 0.99 | S | conserved | annotated^2^ |  |
| 166 | Bdi-MIR166a | 1 | + | 6,574,370 | 6,574,504 | 1 | 0.92 | S | conserved | annotated^1,3^ |  |
|  | Bdi-MIR166b | 1 | - | 30,655,630 | 30,655,851 | 1 | 0.94 | S | conserved | annotated^3^ |  |
|  | Bdi-MIR166c | 3 | + | 33,098,776 | 33,098,923 | 1 | 0.92 | S | conserved | annotated^3^ |  |
|  | Bdi-MIR166d | 1 | - | 71,419,403 | 71,419,518 | 1 | 0.93 | S | conserved | annotated^3^ |  |
|  | Bdi-MIR166e | 3 | + | 51,437,882 | 51,438,002 | 1 | 0.94 | S | conserved | annotated^3^ |  |
|  | Bdi-MIR166f | 4 | - | 6,090,880 | 6,090,999 | 1 | 0.89 | S | conserved | annotated^3^ |  |
|  | Bdi-MIR166g | 4 | + | 37,627,843 | 37,628,050 | 1 | 0.87 | H | conserved | annotated^3^ |  |
|  | Bdi-MIR166h | 3 | + | 57,427,325 | 57,427,477 | 1 | 0.93 | S | conserved | newly identified |  |
|  | Bdi-MIR166i | 3 | - | 27,457,693 | 27,457,817 | 1 | 0.94 | S | conserved | newly identified |  |
|  | Bdi-MIR166j | 3 | + | 57,427,224 | 57,427,375 | 1 | 0.75 | S | conserved | newly identified |  |
| 167 | Bdi-MIR167a | 1 | + | 6,349,049 | 6,349,174 | 1 | 0.97 | S | conserved | annotated^1,3^ |  |
|  | Bdi-MIR167b | 1 | + | 3,770,025 | 3,770,170 | 1 | 0.96 | S | conserved | annotated^3^ |  |
|  | Bdi-MIR167c | 1 | + | 54,067,084 | 54,067,222 | 1 | 0.97 | S | conserved | annotated^3^ |  |
|  | Bdi-MIR167d | 3 | - | 3,632,427 | 3,632,586 | 1 | 0.97 | S | conserved | annotated^3^ |  |
|  | Bdi-MIR167e | 4 | - | 1,641,428 | 1,641,565 | 1 | 0.97 | S | conserved | newly identified |  |
|  | Bdi-MIR167f | 4 | - | 1,643,731 | 1,643,832 | 1 | 0.97 | S | conserved | newly identified |  |
|  | Bdi-MIR167g | 1 | - | 71,590,930 | 71,591,103 | 1 | 0.97 | S | conserved | newly identified |  |
| 168 | Bdi-MIR168 | 3 | - | 1,774,720 | 1,774,823 | 1 | 0.99 | S | conserved | annotated^2,3^ |  |
| 169 | Bdi-MIR169a | 2 | + | 7,704,155 | 7,704,288 | 1 | 0.65 | H | conserved | newly identified |  |
|  | Bdi-MIR169b | 1 | - | 27,159,079 | 27,159,231 | 1 | 0.77 | S | conserved | annotated^1,3^ |  |
|  | Bdi-MIR169c | 5 | - | 23,763,877 | 23,764,012 | 1 | 0.79 | S | conserved | annotated^3^ |  |
|  | Bdi-MIR169d | 4 | + | 26,242,415 | 26,242,581 | 1 | 0.72 | S | conserved | annotated^3^ |  |
|  | Bdi-MIR169e | 3 | + | 43,441,523 | 43,441,689 | 1 | 0.87 | S | conserved | annotated^3^ |  |
|  | Bdi-MIR169g | 3 | + | 43,444,486 | 43,444,666 | 1 | 0.75 | H | conserved | annotated^3^ |  |
|  | Bdi-MIR169h | 5 | - | 11,563,844 | 11,563,989 | 1 | 0.83 | S | conserved | annotated^3^ |  |
|  | Bdi-MIR169j | 4 | + | 44,513,754 | 44,513,936 | 1 | 0.68 | H | conserved | annotated^3^ |  |
|  | Bdi-MIR169k | 1 | + | 1,175,438 | 1,175,583 | 1 | 0.64 | H | conserved | annotated^3^ |  |
|  | Bdi-MIR169l | 4 | - | 35,762,160 | 35,762,251 | 1 | 0.88 | H | conserved | newly identified |  |
|  | Bdi-MIR169m | 3 | + | 58,636,718 | 58,636,827 | 1 | 0.85 | S | conserved | newly identified |  |
|  | Bdi-MIR169n | 1 | - | 11,346,973 | 11,347,080 | 1 | 0.9 | S | conserved | newly identified |  |
| 171 | Bdi-MIR171b | 1 | + | 6,911,151 | 6,911,282 | 1 | 0.98 | S | conserved | annotated^3^ |  |
|  | Bdi-MIR171c | 5 | - | 24,711,137 | 24,711,229 | 1 | 0.99 | H | conserved | annotated^1^ |  |
|  | Bdi-MIR171d | 1 | - | 72,765,318 | 72,765,447 | 1 | 0.96 | S | conserved | annotated^3^ |  |
|  | Bdi-MIR171e | 1 | - | 30,824,648 | 30,824,742 | 1 | 0.98 | S | conserved | newly identified |  |
|  | Bdi-MIR171f | 5 | + | 21,672,257 | 21,672,347 | 1 | 0.91 | S | conserved | newly identified |  |
| 172 | Bdi-MIR172a | 3 | - | 55,737,324 | 55,737,434 | 1 | 0.99 | S | conserved | annotated^3^ |  |
|  | Bdi-MIR172b | 2 | - | 58,915,775 | 58,915,988 | 1 | 0.89 | S | conserved | annotated^3^ |  |
| 390 | Bdi-MIR390a | 1 | + | 2,722,080 | 2,722,265 | 0.99 | 0.73 | S | conserved | annotated^3^ |  |
| 393 | Bdi-MIR393a | 2 | - | 2,001,028 | 2,001,136 | 1 | 0.88 | S | conserved | annotated^2,3^ |  |
|  | Bdi-MIR393b | 5 | + | 27,613,851 | 27,613,959 | 1 | 0.53 | H | conserved | annotated^3^ |  |
| 394 | Bdi-MIR394 | 3 | - | 52,316,402 | 52,316,543 | 1 | 0.87 | S | conserved | annotated^2,3^ |  |
| 395 | Bdi-MIR395a | 4 | + | 16,374,451 | 16,374,593 | 0.99 | 0.94 | S | conserved | annotated^2,3^ |  |
|  | Bdi-MIR395b | 1 | - | 55,440,734 | 55,440,819 | 0.99 | 0.95 | S | conserved | annotated^2,3^ |  |
|  | Bdi-MIR395c | 5 | + | 25,455,924 | 25,456,053 | 0.94 | 0.87 | H | conserved | annotated^2,3^ |  |
|  | Bdi-MIR395d | 3 | - | 14,764,322 | 14,764,424 | 1 | 0.66 | H | conserved | annotated^3^ |  |
|  | Bdi-MIR395e | 5 | + | 25,455,201 | 25,455,340 | 0.99 | 0.85 | S | conserved | annotated^2^ |  |
|  | Bdi-MIR395f | 5 | + | 25,455,484 | 25,455,612 | 0.97 | 0.83 | S | conserved | annotated^2^ |  |
|  | Bdi-MIR395g | 5 | + | 25,455,725 | 25,455,811 | 0.95 | 0.87 | S | conserved | annotated^2^ |  |
|  | Bdi-MIR395h | 5 | + | 25,455,887 | 25,455,952 | 0.91 | 0.81 | H | conserved | annotated^2^ |  |
|  | Bdi-MIR395j | 5 | + | 25,456,063 | 25,456,189 | 0.95 | 0.44 | H | conserved | annotated^2^ |  |
|  | Bdi-MIR395k | 5 | + | 25,456,199 | 25,456,328 | 0.95 | 0.44 | H | conserved | annotated^2^ |  |
|  | Bdi-MIR395l | 5 | + | 25,456,562 | 25,456,657 | 0.97 | 0.88 | H | conserved | annotated^2^ |  |
|  | Bdi-MIR395m | 1 | - | 55,440,157 | 55,440,247 | 1 | 0.96 | S | conserved | annotated^2^ |  |
|  | Bdi-MIR395n | 5 | + | 25,457,119 | 25,457,204 | 0.93 | 0.87 | S | conserved | annotated^2^ |  |
|  | Bdi-MIR395o | 1 | - | 55,440,489 | 55,440,616 | 0.94 | 0.87 | S | conserved | newly identified |  |
|  | Bdi-MIR395p | 5 | + | 25,456,423 | 25,456,522 | 0.61 | 0.41 | H | conserved | newly identified |  |
|  | Bdi-MIR395q | 5 | + | 25,456,843 | 25,456,926 | 0.99 | 0.97 | S | conserved | newly identified |  |
| 396 | Bdi-MIR396a | 3 | - | 59,349,800 | 59,349,975 | 1 | 0.94 | S | conserved | annotated^2,3^ |  |
|  | Bdi-MIR396b | 5 | + | 27,112,486 | 27,112,628 | 1 | 0.96 | S | conserved | annotated^3^ |  |
|  | Bdi-MIR396c | 3 | - | 54,968,153 | 54,968,275 | 1 | 0.57 | H | conserved | annotated^3^ |  |
|  | Bdi-MIR396d | 1 | - | 46,677,004 | 46,677,155 | 1 | 0.56 | H | conserved | annotated^3^ |  |
|  | Bdi-MIR396e | 3 | + | 54,962,856 | 54,963,007 | 1 | 0.62 | H | conserved | annotated^3^ |  |
| 397 | Bdi-MIR397a | 3 | - | 3,149,714 | 3,149,810 | 1 | 0.93 | S | conserved | annotated^1,3^ |  |
|  | Bdi-MIR397b | 3 | - | 3,177,772 | 3,177,886 | 1 | 0.93 | S | conserved | newly identified |  |
| 398 | Bdi-MIR398a | 2 | - | 35,924,432 | 35,924,545 | 1 | 0.66 | H | conserved | annotated^3^ |  |
|  | Bdi-MIR398b | 3 | + | 21,918,241 | 21,918,359 | 1 | 0.47 | H | conserved | annotated^3^ |  |
| 399 | Bdi-MIR399b | 3 | + | 7,135,280 | 7,135,410 | 1 | 0.53 | H | conserved | annotated^3^ |  |
|  | Bdi-MIR399c | 2 | + | 16,765,663 | 16,765,802 | 0.88 | 0.82 | H | conserved | newly identified |  |
|  | Bdi-MIR399d | 3 | - | 7,132,920 | 7,133,061 | 1 | 0.93 | S | conserved | newly identified |  |
| 408 | Bdi-MIR408 | 2 | + | 10,450,362 | 10,450,513 | 1 | 0.46 | H | conserved | annotated^4,3^ |  |
| 444 | Bdi-MIR444a | 3 | + | 38,887,737 | 38,888,859 | 1.00 | 0.93 | M | conserved | newly identified |  |
|  | Bdi-MIR444b | 3 | + | 56,701,604 | 56,704,223 | 1.00 | 0.58 | M | conserved | newly identified |  |
|  | Bdi-MIR444c | 3 | - | 48,666,800 | 48,671,168 | 1.00 | 0.56 | M | conserved | newly identified |  |
|  | Bdi-MIR444d | 5 | - | 15,919,916 | 15,922,479 | 1.00 | 0.60 | M | conserved | newly identified |  |
| 528 | Bdi-MIR528 | 1 | - | 73,059,633 | 73,059,853 | 1 | 0.99 | S | conserved | annotated^2,3^ |  |
| 530 | Bdi-MIR530a | 5 | - | 16,956,517 | 16,956,678 | 1 | 0.83 | H | conserved | newly identified |  |
|  | Bdi-MIR530b | 5 | - | 16,956,889 | 16,957,043 | 0.99 | 0.63 | H | conserved | newly identified |  |
| 531 | Bdi-MIR531 | 2 | + | 9,542,419 | 9,542,492 | 1 | 0.78 | S | conserved | newly identified |  |
| 827 | Bdi-MIR827 | 5 | - | 18,037,487 | 18,037,635 | 1 | 0.89 | S | conserved | annotated^2,3^ |  |
| 845 | Bdi-MIR845 | 3 | - | 17,791,439 | 17,791,533 | 0.97 | 0.91 | S | conserved | newly identified |  |
| 1432 | Bdi-MIR1432 | 1 | + | 18,961,246 | 18,961,366 | 1 | 0.92 | S | conserved | newly identified |  |
| 2118 | Bdi-MIR2118a | 5 | - | 13,861,046 | 13,861,241 | 1 | 0.83 | M | conserved | newly identified |  |
|  | Bdi-MIR2118b | 5 | - | 13,861,144 | 13,861,372 | 1 | 0.67 | M | conserved | newly identified |  |
| 2275 | Bdi-MIR2275a | 3 | + | 12,513,972 | 12,514,077 | 1 | 0.92 | S | conserved | newly identified |  |
|  | Bdi-MIR2275b | 3 | + | 13,941,819 | 13,941,925 | 1 | 0.98 | S | conserved | newly identified |  |
|  | Bdi-MIR2275c | 3 | + | 12,514,129 | 12,514,225 | 0.99 | 0.78 | H | conserved | newly identified |  |
| 5163 | Bdi-MIR5163a | 4 | - | 11,947,799 | 11,948,046 | 1 | 0.9 | H | annotated | annotated^3^ |  |
|  | Bdi-MIR5163b | 4 | - | 11,948,707 | 11,948,885 | 1 | 0.93 | S | novel | newly identified |  |
| 5169 | Bdi-MIR5169 | 4 | - | 42,248,188 | 42,248,351 | 0.98 | 0.81 | S | annotated | annotated^3^ |  |
|  | Bdi-MIR5169b | 5 | - | 20,527,418 | 20,527,519 | 0.93 | 0.89 | S | annotated | newly identified |  |
| 5171 | Bdi-MIR5171b | 2 | - | 11,086,341 | 11,086,534 | 0.91 | 0.42 | H | annotated | newly identified |  |
| 5173 | Bdi-MIR5173 | 1 | - | 22,135,908 | 22,136,071 | 1 | 0.79 | S | annotated | annotated^3^ |  |
| 5174 | Bdi-MIR5174b | 2 | + | 38,216,527 | 38,216,583 | 0.94 | 0.76 | S | annotated | newly identified |  |
|  | Bdi-MIR5174d | 4 | + | 47,097,725 | 47,097,810 | 0.91 | 0.79 | S | annotated | newly identified |  |
|  | Bdi-MIR5174f | 3 | - | 18,576,851 | 18,576,934 | 0.9 | 0.86 | S | annotated | newly identified |  |
| 5176 | Bdi-MIR5176 | 1 | - | 28,306,180 | 28,306,285 | 0.99 | 0.77 | S | annotated | annotated^2^ |  |
| 5178 | Bdi-MIR5178 | 3 | - | 8,463,679 | 8,463,864 | 1 | 0.5 | H | annotated | annotated^3^ |  |
| 5179 | Bdi-MIR5179 | 3 | - | 9,493,281 | 9,493,494 | 1 | 0.93 | S | conserved | annotated^3^ |  |
| 5181 | Bdi-MIR5181c | 2 | - | 22,827,671 | 22,827,757 | 0.97 | 0.85 | S | annotated | newly identified |  |
|  | Bdi-MIR5181e | 1 | - | 63,715,748 | 63,715,844 | 0.96 | 0.83 | S | novel | newly identified |  |
| 5182 | Bdi-MIR5182 | 2 | - | 51,090,561 | 51,090,740 | 0.99 | 0.56 | H | annotated | annotated^3^ |  |
| 5185 | Bdi-MIR5185l | 1 | - | 50,986,585 | 50,986,683 | 0.98 | 0.88 | S | novel | newly identified |  |
| 5199 | Bdi-MIR5199 | 1 | - | 42,791,360 | 42,791,615 | 0.96 | 0.56 | H | annotated | annotated^2^ |  |
| 5200 | Bdi-MIR5200a | 1 | + | 47,454,228 | 47,454,322 | 0.99 | 0.95 | S | annotated | newly identified |  |
|  | Bdi-MIR5200b | 1 | + | 47,483,420 | 47,483,526 | 1 | 0.95 | S | annotated | annotated^2^ |  |
| 5201 | Bdi-MIR5201 | 2 | - | 42,461,398 | 45,461,540 | 0.99 | 0.62 | H | annotated | annotated^2^ |  |
| 5281 | Bdi-MIR5281a | 3 | + | 11,656,552 | 11,656,636 | 1 | 0.72 | S | conserved | newly identified |  |
|  | Bdi-MIR5281b | 3 | - | 48,737,185 | 48,737,430 | 0.92 | 0.87 | S | conserved | newly identified |  |
| 7731 | Bdi-NIR7731 | 4 | + | 4,382,748 | 4,382,918 | 1 | 0.95 | S | novel | newly identified |  |
| 7738 | Bdi-MIR7738 | 3 | - | 47,406,348 | 47,406,440 | 0.99 | 0.9 | S | novel | newly identified |  |
| 7754 | Bdi-MIR7754 | 3 | - | 22,735,435 | 22,735,530 | 1 | 0.92 | S | novel | newly identified |  |
| 7782 | Bdi-MIR7782 | 3 | + | 30,460,948 | 30,461,090 | 1 | 0.92 | S | conserved | newly identified |  |
| 9480 | Bdi-MIR9480a | 4 | - | 31,011,548 | 31,011,617 | 0.98 | 0.93 | S | novel | newly identified |  |
|  | Bdi-MIR9480b | 5 | - | 22,720,969 | 22,721,075 | 1 | 0.95 | S | novel | newly identified |  |
| 9481 | Bdi-MIR9481a | 4 | - | 4,930,203 | 4,930,325 | 0.94 | 0.85 | S | novel | newly identified |  |
|  | Bdi-MIR9481b | 4 | - | 4,929,512 | 4,929,646 | 0.94 | 0.74 | S | novel | newly identified |  |
| 9482 | Bdi-MIR9482 | 4 | - | 18,009,072 | 18,009,224 | 1 | 0.86 | S | novel | newly identified |  |
| 9483 | Bdi-MIR9483a | 3 | - | 2,804,863 | 2,805,036 | 1 | 0.79 | S | novel | newly identified |  |
|  | Bdi-MIR9483b | 3 | - | 2,804,995 | 2,805,256 | 1 | 0.94 | S | novel | newly identified |  |
| 9484 | Bdi-MIR9484 | 2 | - | 43,034,395 | 43,034,529 | 0.94 | 0.89 | S | novel | newly identified |  |
| 9485 | Bdi-MIR9485 | 2 | + | 9,203,374 | 9,203,540 | 1 | 0.78 | S | novel | newly identified |  |
| 9486 | Bdi-MIR9486a | 4 | - | 7,498,369 | 7,498,481 | 0.97 | 0.71 | S | novel | newly identified |  |
|  | Bdi-MIR9486b | 3 | - | 10,710,427 | 10,710,554 | 0.98 | 0.75 | S | novel | newly identified |  |
| 9487 | Bdi-MIR9487 | 2 | + | 25,442,100 | 25,442,286 | 1 | 0.85 | S | novel | newly identified |  |
| 9488 | Bdi-MIR9488 | 1 | - | 50,046,751 | 50,046,943 | 1 | 0.9 | S | novel | newly identified |  |
| 9489 | Bdi-MIR9489 | 2 | - | 49,503,475 | 49,503,595 | 1 | 0.9 | S | novel | newly identified |  |
| 9490 | Bdi-MIR9490 | 3 | - | 18,715,092 | 18,715,183 | 0.93 | 0.7 | S | novel | newly identified |  |
| 9491 | Bdi-MIR9491 | 1 | + | 14,711,883 | 14,711,980 | 0.99 | 0.73 | S | novel | newly identified |  |
| 9492 | Bdi- MIR9492 | 1 | - | 25,584,132 | 25,584,222 | 1 | 0.8 | S | novel | newly identified |  |
| 9493 | Bdi- MIR9493 | 4 | - | 36,753,406 | 36,753,517 | 0.96 | 0.75 | S | novel | newly identified |  |
| 9494 | Bdi- MIR9494 | 1 | + | 2,643,359 | 2,643,510 | 0.99 | 0.79 | S | novel | newly identified |  |
| 9495 | Bdi- MIR9495 | 3 | - | 7,679,238 | 7,679,324 | 1 | 0.91 | S | novel | newly identified |  |
| 9496 | Bdi- MIR9496 | 3 | - | 1,747,940 | 1,748,017 | 0.96 | 0.92 | S | novel | newly identified |  |
| 9497 | Bdi- MIR9497 | 2 | - | 49,315,484 | 49,315,725 | 0.97 | 0.71 | S | novel | newly identified |  |
| 9498 | Bdi- MIR9498 | 1 | + | 38,422,189 | 38,422,246 | 1 | 0.78 | S | novel | newly identified |  |
| 9499 | Bdi- MIR9499 | 4 | - | 17,265,172 | 17,265,253 | 1 | 0.73 | S | novel | newly identified |  |
| ^1^Planta 230:659-669(2009) |  |  |  |  |  |  |  |  |  |  |  |
| ^2^BMC Genomics 10:449(2009) | |  |  |  |  |  |  |  |  |  |  |
| ^3^Genomics 97:282-293(2011) | | |  |  |  |  |  |  |  |  |  |
| ^4^BMC Genomics 12:129(2011) | | |  |  |  |  |  |  |  |  |  |
| H - Homology pipeline | | |  |  |  |  |  |  |  |  |  |
| M - Manual identification | |  |  |  |  |  |  |  |  |  |  |
| S - stringent pipeline | |  |  |  |  |  |  |  |  |  |  |
|  | |  |  |  |  |  |  |  |  |  |  |

**Table S2. Conserved and Non-conserved Annotated miRNAs.**

miRNA gene families were grouped based on the sequence similarity. The sequences of miRNA family members were aligned, and nucleotides that differ are shown in red. Abundance is from sum of TP2M values from all the libraries.

| Family | Mature miRNA | Sequence | Length | Abundance |
| --- | --- | --- | --- | --- |
| 156/529 | Bdi-miR156b-i | UGACAGAAGAGAGUGAGCAC | 20 | 1,060,915 |
|  | Bdi-miR156j | UGACAGAAGAGAGAGAGCAC | 20 | 752 |
|  | Bdi-miR529 | ----AGAAGAGAGAGAGUACAGCCU | 21 | 888 |
| 159/319 | Bdi-miR159b | UUUGGAUUGAAGGGAGCUCUG | 21 | 146,343 |
|  | Bdi-miR159c | UUUGGUUUGAAGGGGGCUCUG | 21 | 35 |
|  | Bdi-miR319a | --UGGACUGAAGGGAGCUCCCUC | 21 | 790 |
|  | Bdi-miR319b | -UUGGACUGAAGGGUGCUCCCU | 21 | 16,051 |
| 160 | Bdi-miR160a-d | UGCCUGGCUCCCUGUAUGCCA | 21 | 3,130 |
|  | Bdi-miR160e | UGCCUGGCUCCCUGAAUGCCA | 21 | 16,193 |
|  | Bdi-miR160f | UGCCUGGCUCCCUGUAUGCC | 20 | 85 |
| 162 | Bdi-miR162 | UCGAUAAACCUCUGCAUCCGG | 21 | 1 |
| 164 | Bdi-miR164abe | UGGAGAAGCAGGGCACGUGCA | 21 | 7,368 |
|  | Bdi-miR164c | UGGAGAAGCAGGGCACGUGCU | 21 | 358 |
|  | Bdi-miR164c.2 | UGCUGGAGAAGCAGGGCACGU | 21 | 949 |
| 166 | Bdi-miR166a-ei | --UCGGACCAGGCUUCAUUCCCC | 21 | 70,179 |
|  | Bdi-miR166f | UCUCGGACCAGGCUUCAUUCC | 21 | 25,400 |
|  | Bdi-miR166h | --UCGGACCAGGCUUCAAUCCCU | 21 | 6,040 |
|  | Bdi-miR166j | --UCGGACCAGGCUUCAUUCCUU | 21 | 4,317 |
|  | Bdi-miR166g | UGUGGUGAUCUCGGACCAGGC | 21 | 2,087 |
| 167 | Bdi-miR167abf | UGAAGCUGCCAGCAUGAUCUA | 21 | 19,018 |
|  | Bdi-miR167cdeg | UGAAGCUGCCAGCAUGAUCUGA | 22 | 183,788 |
| 168 | Bdi-miR168 | UCGCUUGGUGCAGAUCGGGAC | 21 | 2,961,266 |
| 169 | Bdi-miR169a | CAGCCAAGGAUGACUUGCCGA | 21 | 17,292 |
|  | Bdi-miR169bn | UAGCCAAGGAUGACUUGCCG | 20 | 8,917 |
|  | Bdi-miR169c | CAGCCAAGGAUGACUUGCCGG | 21 | 12,866 |
|  | Bdi-miR169d | UAGCCAAGAAUGACUUGCCUA | 21 | 701 |
|  | Bdi-miR169e | UAGCCAAGGAUGACUUGCCUG | 21 | 3,599 |
|  | Bdi-miR169g-3p | UAGCCAGGAAUGGCUUGCCUA | 21 | 12 |
|  | Bdi-miR169hm | UAGCCAAGGAUGACUUGCCUA | 21 | 2,366 |
|  | Bdi-miR169k-5p | UAGCCAAGGAUGAUUUGCCUGUA | 23 | 975 |
|  | Bdi-miR169l | UAGCCAAGGAUGAAUUGCCGG | 21 | 8 |
|  | Bdi-miR169k-3p | UGGGCAAGUCAGCCUGGCUACC | 22 | 1,165 |
| 171 | Bdi-miR171b-e | UGAUUGAGCCGUGCCAAUAUC | 21 | 24,242 |
|  | Bdi-miR171f | ----UGAGCCGAACCAAUAUCACCC | 21 | 5 |
|  | Bdi-miR171f-5p | CAUGGUAUUGUUUCGGCUCAUG | 22 | 117 |
| 172 | Bdi-miR172a | AGAAUCUUGAUGAUGCUGCAU | 21 | 139,702 |
|  | Bdi-miR172b | GGAAUCUUGAUGAUGCUGCAU | 21 | 2,082 |
| 390 | Bdi-miR390a | AAGCUCAGGAGGGAUAGCGCC | 21 | 1,415 |
| 393 | Bdi-miR393ab | UCCAAAGGGAUCGCAUUGAUC | 21 | 1,711 |
|  | Bdi-miR393b-3p | UCAGUGCAAUCCCUUUGGAAU | 21 | 4,884 |
| 394 | Bdi-miR394 | UUGGCAUUCUGUCCACCUCC | 20 | 2,330 |
| 395 | Bdi-miR395a-ce-hj-oq | UGAAGUGUUUGGGGGAACUC | 20 | 1,355 |
|  | Bdi-miR395d | --AAGUGUUUGGGGAACUCUAGG | 21 | 172 |
|  | Bdi-miR395p | UGAAGUGUUUGGAGGAACUC | 20 | 53 |
| 396 | Bdi-miR396ab | -UCCACAGGCUUUCUUGAACUG | 21 | 82,150 |
|  | Bdi-miR396cd | UUCCACAG-CUUUCUUGAACUG | 21 | 418 |
|  | Bdi-miR396e | UUCCACAG-CUUUCUUGAACUU | 21 | 3,142 |
| 397 | Bdi-miR397ab | AUUGAGUGCAGCGUUGAUGAA | 21 | 30,860 |
| 398 | Bdi-miR398a | UGUGUUCUCAGGUCGCCCCUG | 21 | 69 |
|  | Bdi-miR398b | UGUGUUCUCAGGUCACCCCUU | 21 | 3 |
| 399 | Bdi-miR399bc | UGCCAAAGGAGAAUUGCCCUG | 21 | 13 |
|  | Bdi-miR399d | UGCCAAAGGAGAUUUGCCCGG | 21 | 152 |
| 408 | Bdi-miR408 | CUGCACUGCCUCUUCCCUGGC | 21 | 499 |
|  | Bdi-miR408-5p | ACAGGGAUGGAGCAGAGCAUG | 21 | 544 |
| 444 | Bdi-miR444ab | UGCAGUUGCUGCCUCAAGCUU | 21 | 2,623 |
|  | Bdi-miR444cd | UGCAGUUGUUGUCUCAAGCUU | 21 | 635 |
|  | Bdi-miR444cd.2 | ------UGUUGUCUCAAGCUUGCUGCC | 21 | 1,904 |
|  | Bdi-miR444b.3 | UUGUGGCUUUCUUGCAAGUUG | 21 | 1,829 |
| 528 | Bdi-miR528 | UGGAAGGGGCAUGCAGAGGAG | 21 | 67,688 |
| 530 | Bdi-miR530ab | UGCAUUUGCACCUGCACCUAC | 21 | 4 |
|  | Bdi-miR530b-3p | AGGUGCAGUGGCAGAUGCAGC | 21 | 62 |
| 531 | Bdi-miR531 | GAUGCUCGCCGGAGCAGCGUGCUG | 24 | 1,334 |
| 827 | Bdi-miR827 | UUAGAUGACCAUCAGCAAACA | 21 | 587 |
| 845 | Bdi-miR845 | UGCUCUGAUACCAAUUGUUGG | 21 | 594 |
| 1432 | Bdi-miR1432 | UUCAGGAGAGAUGACACCGACA | 22 | 6,157 |
| 2118 | Bdi-miR2118a | UUUCCGAUGCCUCCCAUUCCUA | 22 | 4 |
|  | Bdi-miR2118b | UUCCUGAUGCCUCCCAUUCCUA | 22 | 8 |
| 2275 | Bdi-miR2275a | UUUGGUUUCCUCCAAUGUCUCA | 22 | 110 |
|  | Bdi-miR2275b | UUCAGUUUCUUCUAAUAUCUCA | 22 | 12 |
|  | Bdi-miR2275c-3p | UUUGGUUUCCUCCAAUAUCUCA | 22 | 26 |
|  | Bdi-miR2275b-5p | ACUAGUCCGUUGCAUUUUGC | 20 | 42 |
|  | Bdi-miR2275c-5p | AGAUUUGGAUGGAACCAAAUC | 21 | 39 |
| 5163 | Bdi-miR5163a | UUAGGUAUUUCAGGUUAGGUG | 21 | 7,423 |
|  | Bdi-miR5163b-3p | UAGAUAUUUCAGGUUGUGUGGA | 22 | 36,348 |
| 5169 | Bdi-miR5169ab | UUUGACCAAGUUUGUAGAACA | 21 | 292 |
| 5171 | Bdi-miR5171b | ACUUAAUAUGGGACGGAAGAA | 21 | 2 |
| 5173 | Bdi-miR5173 | UCUCGUAUAUGCGGAUGUACC | 21 | 257 |
| 5174 | Bdi-miR5174bdf | CCUCCGUUUCAUAAAGGUUGG | 21 | 367 |
| 5176 | Bdi-miR5176 | UGUGAUGAUGUGGCAUAGAAU | 21 | 1,441 |
| 5178 | Bdi-miR5178 | UCUGACCGGUGGGCCUGAGCG | 21 | 153 |
| 5179 | Bdi-miR5179 | UUUUGCUCAAGACCGCGCAAC | 21 | 1,179 |
| 5181 | Bdi-miR5181c | ACUUCUUAUGGAUUGUAGGGA | 21 | 136 |
|  | Bdi-miR5181e | CGACACUUACUGUGGCUCGGA | 21 | 277 |
| 5182 | Bdi-miR5182 | UGAUGAUCUUGGAACACGUGC | 21 | 453 |
| 5185 | Bdi-miR5185l-3p.2 | UGGAGAUUGACUUAGAAGCGG | 21 | 360 |
| 5199 | Bdi-miR5199 | UGUUCAUACGGUUGAUAGCAC | 21 | 84 |
| 5200 | Bdi-miR5200ab | UGUAGAUACUCCCUAAGGCUU | 21 | 901 |
| 5201 | Bdi-miR5201 | AGGGCGAGGCAAAUGAUCAAA | 21 | 51 |
| 5281 | Bdi-miR5281ab | UCUUAUAAAUAGGAACGGAGG | 21 | 50 |
| 7731 | Bdi-miR7731 | AACAAGGGAUGCACAUACUUUGAG | 24 | 836 |
| 7738 | Bdi-miR7738-3p | GUGCUUGACAGACGACUCUGG | 21 | 446 |
| 7754 | Bdi-miR7754-3p | UUCUCUCGGCUAAGGAACUGC | 21 | 392 |
| 7782 | Bdi-miR7782 | ACCUGCUCUGAUACCAUGUUGUGA | 24 | 64,824 |
| 9480 | Bdi-miR9480ab | UAUGUGAGGGUGGUAACUGAA | 21 | 1,645 |
|  |  |  |  |  |
| 9481 | Bdi-miR9481a | UCAGUCGGAUUUCUCACCUUCGAA | 24 | 384 |
|  | Bdi-miR9481b | UCAGUCGGAUUUCUCACCUUC | 21 | 73 |
| 9482 | Bdi-miR9482 | CCUUUGGGGAAGAAGGGAAAC | 21 | 339 |
| 9483 | Bdi-miR9483ab | UUGAACUGUUUCCUCUGAAGUUCC | 24 | 317 |
| 9484 | Bdi- miR9484 | UAGUGCAGGGAGAAGUCGGUC | 21 | 259 |
| 9485 | Bdi- miR9485 | UUAUGACGUGUAGGAGUUGCA | 21 | 260 |
| 9486 | Bdi-miR9486a.1 | AUGCUUUCAAGGGAUUAGAGGUUC | 24 | 254 |
|  | Bdi-miR9486a.2 | UCUAAUGGCUGAAAUGGGAAG | 21 | 183 |
|  | Bdi-miR9486b | UAAGUGAUUAGAGGUUCCAGU | 21 | 121 |
| 9487 | Bdi-miR9487 | CCUUGUUCGAUUGCAAGAUGA | 21 | 174 |
| 9488 | Bdi- miR9488 | UGAGGGCUAGGCUUUUAUGUAA | 22 | 161 |
| 9489 | Bdi- miR9489 | UCAGCUCCACGGACUUGGUGA | 21 | 144 |
| 9490 | Bdi- miR9490 | AGGCCACACCCUAAUGGUCGUGCG | 24 | 111 |
| 9491 | Bdi- miR9491 | UGGUAUGUUACCUCUGAUCAG | 21 | 69 |
| 9492 | Bdi- miR9492 | UAUCUACUCUGUCAUGGUAUC | 21 | 60 |
| 9493 | Bdi- miR9493 | AAGAAUUAUGAAACGAAGGGAGUA | 24 | 60 |
| 9494 | Bdi- miR9494 | UUCAUCACCUUCGUCUCCGUC | 21 | 56 |
| 9495 | Bdi- miR9495 | UGAAAAAUGCCUCUGGACGUG | 21 | 55 |
| 9496 | Bdi- miR9496 | CUGGUUGGGCUUAGAUGGGUCC | 22 | 44 |
| 9497 | Bdi- miR9497 | UUUCUGAAUACAUGGUGUAUC | 21 | 35 |
| 9498 | Bdi- miR9498 | GACCGUCAAGUGGUUGUUGAG | 21 | 23 |
| 9499 | Bdi- miR9499 | CCCUCGUCGACGCGGCAGCUC | 21 | 86 |

**Table S3. miRNA Targets with PARE Sequences at the Predicted Cleavage Sites.**

^a^The sum of abundance of the PARE sequence in all four libraries. ^b^NP indicates that the score was over the cutoff for the program, i.e. not predicted. ^c^A= Abundance, T= Rank, P=Peak %.

|  |  |  |  |  |  |  | Score^b^ |  |  |
| --- | --- | --- | --- | --- | --- | --- | --- | --- | --- |
| Family | miRNA(s) | cDNA | Cleavage site (nt) | Max Level | Sum of Abudance (TP10M)^a^ | CleaveLand | CleaveLandM | psRNATarget | Criteria passed^c^ |
| 156/529 | Bdi-miR156b-i | Bradi3g40030.1 | 783 | 4 | 77 | 1 | 1 | 1 | A,T,P |
|  | Bdi-miR156b-i | Bradi4g18900.1 | 429 | 4 | 17 | 1 | 1 | 1 | A,T,P |
|  | Bdi-miR156b-i | Bradi4g33770.1 | 825 | 4 | 77 | 1 | 1 | 1 | A,T,P |
|  | Bdi-miR156b-i | Bradi4g34667.1 | 864 | 4 | 93 | 1 | 1 | 1 | A,T,P |
|  | Bdi-miR156b-i | Bradi2g59110.1 | 840 | 3 | 78 | 1 | 1 | 1 | A,T |
|  | Bdi-miR156b-i | Bradi3g41250.1 | 960 | 3 | 51 | 1 | 1 | 1 | A,T |
|  | Bdi-miR156b-i | Bradi3g03510.1 | 1176 | 3 | 27 | 2 | 2 | 2 | A,T |
|  | Bdi-miR156b-i | Bradi3g05510.1 | 747 | 3 | 80 | 2 | 2 | 2 | A,T |
|  | Bdi-miR156j | Bradi3g40030.1 | 783 | 4 | 77 | 0 | 0 | 0 | A,T,P |
|  | Bdi-miR156j | Bradi4g18900.1 | 429 | 4 | 17 | 0 | 0 | 0 | A,T,P |
|  | Bdi-miR156j | Bradi4g33770.1 | 825 | 4 | 77 | 0 | 0 | 0 | A,T,P |
|  | Bdi-miR156j | Bradi4g34667.1 | 864 | 4 | 93 | 0 | 0 | 0 | A,T,P |
|  | Bdi-miR156j | Bradi2g59110.1 | 840 | 3 | 78 | 0 | 0 | 0 | A,T |
|  | Bdi-miR156j | Bradi3g41250.1 | 960 | 3 | 51 | 0 | 0 | 0 | A,T |
|  | Bdi-miR156j | Bradi3g03510.1 | 1176 | 3 | 27 | 1 | 1 | 1 | A,T |
|  | Bdi-miR156j | Bradi3g05510.1 | 747 | 3 | 80 | 1 | 1 | 1 | A,T |
|  | Bdi-miR529 | Bradi4g33770.1 | 821 | 4 | 76 | 2.5 | 2.5 | 2.5 | A,T,P |
|  | Bdi-miR529 | Bradi3g40030.1 | 779 | 4 | 76 | 2.5 | 2.5 | 2.5 | A,T,P |
|  | Bdi-miR529 | Bradi2g59110.1 | 836 | 1 | 2 | 3.5 | 3.5 | 3.5 |  |
|  | Bdi-miR529 | Bradi3g41250.1 | 956 | 1 | 3 | NP | NP | 2 |  |
|  | Bdi-miR529 | Bradi4g34667.1 | 860 | 1 | 1 | NP | NP | 3 |  |
| 159/319 | Bdi-miR159b | Bradi2g53010.1 | 970 | 3 | 37 | 3.5 | 2 | 3 | A,T |
|  | Bdi-miR159b | Bradi1g15580.1 | 550 | 3 | 1 | 4 | 2 | NP | T,P |
|  | Bdi-miR159b | Bradi1g36540.1 | 736 | 1 | 1 | 3.5 | 1.5 | 3 |  |
|  | Bdi-miR159b | Bradi1g10140.2 | 144 | 1 | 5 | 7 | 4 | NP |  |
|  | Bdi-miR159c | Bradi2g53010.1 | 970 | 3 | 37 | NP | NP | 5 | A,T |
|  | Bdi-miR159c | Bradi3g37620.1 | 1864 | 1 | 2 | 6.5 | 4 | NP |  |
|  | Bdi-miR159c | Bradi1g36540.1 | 736 | 1 | 1 | NP | NP | 5 |  |
|  | Bdi-miR319b | Bradi4g01547.1 | 719 | 4 | 53 | 4 | 3 | 3 | A,T,P |
|  | Bdi-miR319b | Bradi1g58450.1 | 1097 | 4 | 203 | NP | NP | 3.5 | A,T,P |
|  | Bdi-miR319b | Bradi1g06460.1 | 953 | 1 | 2 | NP | NP | 3.5 |  |
|  | Bdi-miR319b | Bradi2g45870.1 | 1634 | 1 | 3 | NP | NP | 4 |  |
| 160 | Bdi-miR160a-d | Bradi3g28950.1 | 1334 | 4 | 870 | 1 | 1 | 1 | A,T,P |
|  | Bdi-miR160a-d | Bradi5g27400.2 | 1154 | 4 | 150 | 0.5 | 0.5 | NP | A,T,P |
|  | Bdi-miR160a-d | Bradi1g33160.1 | 1403 | 4 | 870 | 1 | 1 | NP | A,T,P |
|  | Bdi-miR160a-d | Bradi3g49320.1 | 1370 | 4 | 1764 | 1 | 1 | NP | A,T,P |
|  | Bdi-miR160a-d | Bradi5g15904.1 | 1418 | 4 | 504 | 1 | 1 | NP | A,T,P |
|  | Bdi-miR160a-d | Bradi1g36430.1 | 701 | 1 | 5 | 7 | NP | NP |  |
|  | Bdi-miR160a-d | Bradi4g25620.1 | 1906 | 1 | 7 | 7 | NP | NP |  |
|  | Bdi-miR160e | Bradi3g28950.1 | 1334 | 4 | 870 | 2 | 2 | 2 | A,T,P |
|  | Bdi-miR160e | Bradi5g27400.2 | 1154 | 4 | 150 | 1 | 1 | NP | A,T,P |
|  | Bdi-miR160e | Bradi1g33160.1 | 1403 | 4 | 870 | 2 | 2 | NP | A,T,P |
|  | Bdi-miR160e | Bradi3g49320.1 | 1370 | 4 | 1764 | 2 | 2 | NP | A,T,P |
|  | Bdi-miR160e | Bradi5g15904.1 | 1418 | 4 | 504 | 2 | 2 | NP | A,T,P |
|  | Bdi-miR160e | Bradi2g24290.1 | 1304 | 1 | 11 | 6 | NP | NP |  |
|  | Bdi-miR160e | Bradi3g51077.1 | 2828 | 1 | 2 | NP | NP | 4.5 |  |
|  | Bdi-miR160e | Bradi5g18540.1 | 2954 | 1 | 6 | NP | NP | 4.5 |  |
|  | Bdi-miR160f | Bradi3g28950.1 | 1334 | 4 | 870 | 0 | 0 | 0 | A,T,P |
|  | Bdi-miR160f | Bradi1g33160.1 | 1403 | 4 | 870 | 0 | 0 | NP | A,T,P |
|  | Bdi-miR160f | Bradi3g49320.1 | 1370 | 4 | 1764 | 0 | 0 | NP | A,T,P |
|  | Bdi-miR160f | Bradi5g15904.1 | 1418 | 4 | 504 | 0 | 0 | NP | A,T,P |
|  | Bdi-miR160f | Bradi5g27400.2 | 1154 | 4 | 150 | 0.5 | 0.5 | NP | A,T,P |
|  | Bdi-miR160f | Bradi4g25620.1 | 1906 | 1 | 7 | 6 | 4 | NP |  |
|  | Bdi-miR160f | Bradi1g36430.1 | 701 | 1 | 5 | 7 | NP | NP |  |
| 162 | Bdi-miR162 | Bradi4g20620.1 | 893 | 1 | 3 | NP | NP | 3 |  |
|  | Bdi-miR162 | Bradi5g09500.1 | 576 | 1 | 4 | NP | NP | 5 |  |
| 164 | Bdi-miR164abe | Bradi4g02060.1 | 670 | 4 | 106 | 2 | 2 | 2 | A,T,P |
|  | Bdi-miR164abe | Bradi4g42720.1 | 59 | 4 | 37 | 3 | 2 | 3 | A,T,P |
|  | Bdi-miR164abe | Bradi5g12407.1 | 628 | 4 | 83 | 4 | 3 | 3 | A,T,P |
|  | Bdi-miR164abe | Bradi3g46900.1 | 697 | 4 | 59 | 4 | 3 | NP | A,T,P |
|  | Bdi-miR164abe | Bradi4g28260.1 | 431 | 3 | 89 | 4.5 | 3.5 | NP | A,T |
|  | Bdi-miR164abe | Bradi1g32660.1 | 652 | 3 | 16 | 2 | 2 | 2 | T,P |
|  | Bdi-miR164abe | Bradi1g41710.1 | 316 | 3 | 5 | 2 | 2 | 2 | T,P |
|  | Bdi-miR164abe | Bradi1g07520.1 | 536 | 2 | 17 | 5.5 | 2.5 | 4.5 | A |
|  | Bdi-miR164abe | Bradi1g24360.1 | 550 | 1 | 1 | 4.5 | 2.5 | 3.5 |  |
|  | Bdi-miR164abe | Bradi3g17287.1 | 643 | 1 | 2 | 4.5 | 3 | NP |  |
|  | Bdi-miR164abe | Bradi3g19860.1 | 940 | 1 | 2 | 6 | 4 | NP |  |
|  | Bdi-miR164abe | Bradi2g43950.1 | 1837 | 1 | 2 | 7 | NP | NP |  |
|  | Bdi-miR164c | Bradi4g02060.1 | 670 | 4 | 106 | 1 | 1 | 1 | A,T,P |
|  | Bdi-miR164c | Bradi5g12407.1 | 628 | 4 | 83 | 4 | 3 | 3 | A,T,P |
|  | Bdi-miR164c | Bradi3g46900.1 | 697 | 4 | 59 | 4 | 3 | NP | A,T,P |
|  | Bdi-miR164c | Bradi4g28260.1 | 431 | 3 | 89 | 5 | NP | NP | A,T |
|  | Bdi-miR164c | Bradi1g32660.1 | 652 | 3 | 16 | 1 | 1 | 1 | T,P |
|  | Bdi-miR164c | Bradi1g41710.1 | 316 | 3 | 5 | 1 | 1 | 1 | T,P |
|  | Bdi-miR164c | Bradi1g68830.1 | 1524 | 1 | 3 | 5.5 | 2.5 | 4 |  |
|  | Bdi-miR164c | Bradi1g24360.1 | 550 | 1 | 1 | 5.5 | 3.5 | 4.5 |  |
|  | Bdi-miR164c | Bradi3g17287.1 | 643 | 1 | 2 | 3.5 | 2 | NP |  |
|  | Bdi-miR164c | Bradi2g43950.1 | 1837 | 1 | 2 | 6 | 3.5 | NP |  |
|  | Bdi-miR164c | Bradi3g19860.1 | 940 | 1 | 2 | 6 | 4 | NP |  |
|  | Bdi-miR164c.2 | Bradi4g28260.1 | 434 | 3 | 17 | 6 | 3 | NP | A,T |
|  | Bdi-miR164c.2 | Bradi3g53520.1 | 105 | 2 | 19 | NP | 3.5 | NP | A |
|  | Bdi-miR164c.2 | Bradi4g02060.1 | 673 | 1 | 5 | 2 | 1 | 2 |  |
|  | Bdi-miR164c.2 | Bradi3g12627.1 | 3145 | 1 | 1 | 5 | 3 | 3 |  |
|  | Bdi-miR164c.2 | Bradi3g46900.1 | 700 | 1 | 2 | 3.5 | 2 | NP |  |
|  | Bdi-miR164c.2 | Bradi1g23450.1 | 1186 | 1 | 2 | 5.5 | 3 | NP |  |
|  | Bdi-miR164c.2 | Bradi4g33550.1 | 1306 | 1 | 1 | 5.5 | 3.5 | NP |  |
|  | Bdi-miR164c.2 | Bradi1g45710.1 | 244 | 1 | 10 | 5.5 | 3.5 | NP |  |
|  | Bdi-miR164c.2 | Bradi2g26240.1 | 307 | 1 | 1 | 6 | 4 | NP |  |
| 166 | Bdi-miR166a-ei | Bradi2g06210.1 | 544 | 4 | 133 | 3 | 2.5 | 3 | A,T,P |
|  | Bdi-miR166a-ei | Bradi3g28970.1 | 586 | 3 | 142 | 3 | 2.5 | 3 | A,T |
|  | Bdi-miR166a-ei | Bradi1g13910.1 | 631 | 3 | 133 | 3 | 2.5 | 3 | A,T |
|  | Bdi-miR166a-ei | Bradi4g01887.1 | 586 | 3 | 133 | 3 | 2.5 | 3 | A,T |
|  | Bdi-miR166a-ei | Bradi1g47670.1 | 2117 | 2 | 9 | NP | NP | 1.5 | T |
|  | Bdi-miR166a-ei | Bradi4g38980.1 | 1353 | 1 | 4 | NP | NP | 4.5 |  |
|  | Bdi-miR166a-ei | Bradi5g18830.1 | 203 | 1 | 2 | NP | NP | 3 |  |
|  | Bdi-miR166f | Bradi1g13910.1 | 633 | 2 | 14 | 2.5 | 2 | 3.5 | A |
|  | Bdi-miR166f | Bradi2g06210.1 | 546 | 2 | 14 | 2.5 | 2 | 3.5 | A |
|  | Bdi-miR166f | Bradi4g01887.1 | 588 | 2 | 14 | 2.5 | 2 | 3.5 | A |
|  | Bdi-miR166f | Bradi3g28970.1 | 588 | 1 | 4 | 2.5 | 2 | 3.5 |  |
|  | bdi-miR166g | Bradi1g40990.1 | 713 | 1 | 1 | NP | NP | 4 |  |
|  | bdi-miR166g | Bradi3g42400.1 | 401 | 1 | 3 | 7 | 4 | NP |  |
|  | Bdi-miR166h | Bradi2g33900.1 | 499 | 4 | 31 | NP | NP | 5 | A,T,P |
|  | Bdi-miR166h | Bradi2g06210.1 | 544 | 4 | 133 | NP | NP | 4 | A,T,P |
|  | Bdi-miR166h | Bradi1g13910.1 | 631 | 3 | 133 | NP | NP | 4 | A,T |
|  | Bdi-miR166h | Bradi3g28970.1 | 586 | 3 | 142 | NP | NP | 4 | A,T |
|  | Bdi-miR166h | Bradi4g01887.1 | 586 | 3 | 133 | NP | NP | 4 | A,T |
|  | Bdi-miR166h | Bradi1g47670.1 | 2117 | 2 | 9 | NP | NP | 3 | T |
|  | Bdi-miR166h | Bradi2g51350.1 | 550 | 1 | 2 | NP | NP | 4 |  |
|  | Bdi-miR166h | Bradi2g05140.1 | 484 | 1 | 3 | NP | NP | 5 |  |
|  | Bdi-miR166h | Bradi4g15750.1 | 914 | 1 | 1 | NP | NP | 5 |  |
|  | Bdi-miR166j | Bradi2g06210.1 | 544 | 4 | 133 | 3 | 2.5 | 3 | A,T,P |
|  | Bdi-miR166j | Bradi1g13910.1 | 631 | 3 | 133 | 3 | 2.5 | 3 | A,T |
|  | Bdi-miR166j | Bradi3g28970.1 | 586 | 3 | 142 | 3 | 2.5 | 3 | A,T |
|  | Bdi-miR166j | Bradi4g01887.1 | 586 | 3 | 133 | 3 | 2.5 | 3 | A,T |
|  | Bdi-miR166j | Bradi2g52350.1 | 682 | 3 | 13 | NP | NP | 5 | A,T |
|  | Bdi-miR166j | Bradi1g47670.1 | 2117 | 2 | 9 | NP | NP | 3.5 | T |
|  | Bdi-miR166j | Bradi3g51577.1 | 646 | 1 | 2 | NP | NP | 4.5 |  |
|  | Bdi-miR166j | Bradi5g18830.1 | 203 | 1 | 2 | NP | NP | 5 |  |
| 167 | Bdi-miR167abf | Bradi4g01730.1 | 2564 | 4 | 215 | 5 | 3 | 4.5 | A,T,P |
|  | Bdi-miR167abf | Bradi1g32547.1 | 2468 | 4 | 215 | 5 | 3 | 4.5 | A,T,P |
|  | Bdi-miR167abf | Bradi3g04920.1 | 2510 | 4 | 249 | 5 | 3 | 4.5 | A,T,P |
|  | Bdi-miR167abf | Bradi5g25767.1 | 2339 | 4 | 215 | 4 | 2 | NP | A,T,P |
|  | Bdi-miR167abf | Bradi1g03480.1 | 867 | 2 | 8 | 6.5 | NP | NP | T |
|  | Bdi-miR167abf | Bradi3g06640.1 | 1150 | 1 | 1 | 7 | NP | NP |  |
|  | Bdi-miR167cdeg | Bradi4g01730.1 | 2564 | 4 | 215 | 5 | 3 | 4.5 | A,T,P |
|  | Bdi-miR167cdeg | Bradi3g04920.1 | 2510 | 4 | 249 | 5 | 3 | 4.5 | A,T,P |
|  | Bdi-miR167cdeg | Bradi5g25767.1 | 2339 | 4 | 215 | 5.5 | 3.5 | NP | A,T,P |
|  | Bdi-miR167cdeg | Bradi1g32547.1 | 2468 | 4 | 215 | NP | NP | 4.5 | A,T,P |
|  | Bdi-miR167cdeg | Bradi1g03480.1 | 867 | 2 | 8 | 7 | NP | NP | T |
|  | Bdi-miR167cdeg | Bradi1g55550.1 | 1459 | 1 | 1 | 6 | 3.5 | 4.5 |  |
| 168 | Bdi-miR168 | Bradi2g45920.1 | 328 | 4 | 16 | NP | NP | 4.5 | A,T,P |
|  | Bdi-miR168 | Bradi3g51077.1 | 343 | 1 | 1 | 5 | 3 | 4.5 |  |
|  | Bdi-miR168 | Bradi3g05060.1 | 1104 | 1 | 1 | 6 | 4 | NP |  |
|  | Bdi-miR168 | Bradi1g52020.1 | 498 | 1 | 11 | NP | NP | 4 |  |
| 169 | Bdi-miR169a | Bradi2g58570.1 | 244 | 3 | 2 | 5.5 | 3.5 | 3.5 | T,P |
|  | Bdi-miR169a | Bradi1g20360.1 | 311 | 1 | 10 | NP | NP | 3.5 |  |
|  | Bdi-miR169a | Bradi1g14940.1 | 1895 | 1 | 1 | NP | NP | 4 |  |
|  | Bdi-miR169bn | Bradi2g58570.1 | 244 | 3 | 2 | NP | NP | 3 | T,P |
|  | Bdi-miR169bn | Bradi2g37630.1 | 1673 | 1 | 3 | 6 | 4 | NP |  |
|  | Bdi-miR169bn | Bradi1g14940.1 | 1895 | 1 | 1 | NP | NP | 3 |  |
|  | Bdi-miR169bn | Bradi1g20360.1 | 311 | 1 | 10 | NP | NP | 4.5 |  |
|  | Bdi-miR169c | Bradi2g58570.1 | 244 | 3 | 2 | 5.5 | 3.5 | 3.5 | T,P |
|  | Bdi-miR169c | Bradi2g24160.2 | 124 | 1 | 1 | 7 | 4 | NP |  |
|  | Bdi-miR169c | Bradi4g28260.1 | 70 | 1 | 1 | 7 | NP | NP |  |
|  | Bdi-miR169c | Bradi1g14940.1 | 1895 | 1 | 1 | NP | NP | 4 |  |
|  | Bdi-miR169c | Bradi1g20360.1 | 311 | 1 | 10 | NP | NP | 4.5 |  |
|  | Bdi-miR169d | Bradi1g20360.1 | 311 | 1 | 10 | NP | NP | 5 |  |
|  | Bdi-miR169e | Bradi2g58570.1 | 244 | 3 | 2 | NP | NP | 4.5 | T,P |
|  | Bdi-miR169e | Bradi2g59920.1 | 682 | 1 | 11 | NP | NP | 5 |  |
|  | Bdi-miR169e | Bradi1g20360.1 | 311 | 1 | 10 | NP | NP | 4.5 |  |
|  | Bdi-miR169e | Bradi1g14940.1 | 1895 | 1 | 1 | NP | NP | 5 |  |
|  | Bdi-miR169g-3p | Bradi1g25460.1 | 1695 | 1 | 1 | NP | NP | 5 |  |
|  | Bdi-miR169g-5p | Bradi2g35187.1 | 1158 | 1 | 1 | 5 | 3.5 | NP |  |
|  | Bdi-miR169g-5p | Bradi1g47090.1 | 1372 | 1 | 1 | 7 | NP | NP |  |
|  | Bdi-miR169hm | Bradi2g58570.1 | 244 | 3 | 2 | NP | NP | 4.5 | T,P |
|  | Bdi-miR169hm | Bradi1g20360.1 | 311 | 1 | 10 | NP | NP | 4 |  |
|  | Bdi-miR169hm | Bradi1g14940.1 | 1895 | 1 | 1 | NP | NP | 5 |  |
|  | Bdi-miR169l | Bradi4g18870.1 | 187 | 1 | 1 | 7 | 3.5 | NP |  |
|  | Bdi-miR169l | Bradi2g35587.1 | 3951 | 1 | 9 | NP | NP | 4.5 |  |
|  | Bdi-miR169l | Bradi1g14940.1 | 1895 | 1 | 1 | NP | NP | 4 |  |
| 171 | Bdi-miR171b-e | Bradi1g52240.1 | 584 | 4 | 309 | 1 | 0.5 | 0.5 | A,T,P |
|  | Bdi-miR171b-e | Bradi3g50930.1 | 1163 | 4 | 277 | 1 | 0.5 | NP | A,T,P |
|  | Bdi-miR171b-e | Bradi1g78230.1 | 833 | 4 | 802 | 1 | 1 | NP | A,T,P |
|  | Bdi-miR171b-e | Bradi3g32890.1 | 899 | 1 | 2 | 2 | 1 | NP |  |
|  | Bdi-miR171b-e | Bradi1g02440.1 | 2241 | 1 | 8 | 5.5 | 3.5 | NP |  |
|  | Bdi-miR171b-e | Bradi1g44177.1 | 10014 | 1 | 4 | 7 | 4 | NP |  |
|  | Bdi-miR171b-e | Bradi1g10780.1 | 257 | 1 | 5 | NP | NP | 4.5 |  |
|  | Bdi-miR171f | Bradi1g67270.1 | 1256 | 1 | 1 | 6 | 4 | 5 |  |
|  | Bdi-miR171f | Bradi1g61350.1 | 528 | 1 | 1 | 6 | NP | 4.5 |  |
|  | Bdi-miR171f-5p | Bradi3g30170.1 | 478 | 1 | 1 | NP | NP | 5 |  |
| 172 | Bdi-miR172a | Bradi1g03880.1 | 1267 | 4 | 187 | 2 | 1.5 | 1.5 | A,T,P |
|  | Bdi-miR172a | Bradi1g30337.1 | 1048 | 4 | 28 | 4 | 2.5 | 3.5 | A,T,P |
|  | Bdi-miR172a | Bradi5g24100.1 | 1318 | 4 | 68 | 5 | 3 | 4.5 | A,T,P |
|  | Bdi-miR172a | Bradi1g53650.1 | 1174 | 4 | 187 | NP | NP | 1.5 | A,T,P |
|  | Bdi-miR172a | Bradi2g37800.1 | 1372 | 4 | 84 | NP | NP | 1.5 | A,T,P |
|  | Bdi-miR172a | Bradi1g07140.1 | 1533 | 1 | 1 | NP | NP | 5 |  |
|  | Bdi-miR172b | Bradi1g53650.1 | 1174 | 4 | 187 | 2.5 | 1.5 | 2 | A,T,P |
|  | Bdi-miR172b | Bradi1g03880.1 | 1267 | 4 | 187 | 2.5 | 1.5 | 2 | A,T,P |
|  | Bdi-miR172b | Bradi2g37800.1 | 1372 | 4 | 84 | 2.5 | 1.5 | 2 | A,T,P |
|  | Bdi-miR172b | Bradi1g30337.1 | 1048 | 4 | 28 | 3 | 2.5 | 2.5 | A,T,P |
|  | Bdi-miR172b | Bradi5g24100.1 | 1318 | 4 | 68 | 4 | 3 | 3.5 | A,T,P |
| 390 | Bdi-miR390a | Bradi4g27480.3 | 489 | 1 | 3 | NP | NP | 4.5 |  |
|  | Bdi-miR390a | Bradi1g32310.1 | 327 | 1 | 1 | NP | NP | 5 |  |
| 393 | Bdi-miR393ab | Bradi5g08680.1 | 1513 | 4 | 55 | 1 | 1.5 | 3 | A,T,P |
|  | Bdi-miR393ab | Bradi2g35720.1 | 1561 | 3 | 69 | 1 | 1.5 | 3 | A,T |
|  | Bdi-miR393ab | Bradi3g09350.1 | 1397 | 1 | 1 | NP | NP | 5 |  |
|  | Bdi-miR393b-3p | Bradi4g41070.1 | 282 | 1 | 1 | NP | NP | 5 |  |
| 394 | Bdi-miR394 | Bradi2g59200.1 | 1099 | 4 | 1200 | 0 | 0 | 0 | A,T,P |
|  | Bdi-miR394 | Bradi3g41490.1 | 1652 | 1 | 5 | 6 | NP | NP |  |
|  | Bdi-miR394 | Bradi4g14097.1 | 758 | 1 | 9 | NP | NP | 3 |  |
|  | Bdi-miR394 | Bradi4g16560.1 | 673 | 1 | 1 | NP | NP | 4 |  |
| 395 | Bdi-miR395a-ce-hj-oq | Bradi1g09030.1 | 338 | 3 | 549 | 2 | 1 | 1 | A,T |
|  | Bdi-miR395a-ce-hj-oq | Bradi1g24110.1 | 127 | 1 | 3 | NP | NP | 3 |  |
|  | Bdi-miR395a-ce-hj-oq | Bradi1g52550.1 | 305 | 1 | 3 | NP | NP | 3.5 |  |
|  | Bdi-miR395a-ce-hj-oq | Bradi3g20730.1 | 761 | 1 | 2 | NP | NP | 4.5 |  |
|  | bdi-miR395d | Bradi3g30590.1 | 619 | 1 | 1 | NP | NP | 4 |  |
|  | Bdi-miR395p | Bradi1g09030.1 | 338 | 3 | 549 | 1 | 0.5 | 0.5 | A,T |
|  | Bdi-miR395p | Bradi5g12740.1 | 286 | 1 | 2 | 6.5 | 4 | NP |  |
|  | Bdi-miR395p | Bradi5g00640.1 | 508 | 1 | 3 | 7 | NP | NP |  |
|  | Bdi-miR395p | Bradi1g24110.1 | 127 | 1 | 3 | NP | NP | 2.5 |  |
|  | Bdi-miR395p | Bradi1g52550.1 | 305 | 1 | 3 | NP | NP | 3 |  |
|  | Bdi-miR395p | Bradi3g20730.1 | 761 | 1 | 2 | NP | NP | 4 |  |
| 396 | Bdi-miR396ab | Bradi3g52547.1 | 494 | 4 | 3853 | 1 | 1 | 1 | A,T,P |
|  | Bdi-miR396ab | Bradi3g57267.1 | 395 | 4 | 159 | 1 | 1 | 1 | A,T,P |
|  | Bdi-miR396ab | Bradi5g20607.1 | 497 | 4 | 3853 | 1 | 1 | 1 | A,T,P |
|  | Bdi-miR396ab | Bradi1g46427.1 | 392 | 4 | 129 | 1 | 1 | 1 | A,T,P |
|  | Bdi-miR396ab | Bradi1g50597.1 | 392 | 4 | 2171 | 1 | 1 | 1 | A,T,P |
|  | Bdi-miR396ab | Bradi3g51685.1 | 509 | 4 | 366 | 1 | 1 | NP | A,T,P |
|  | Bdi-miR396ab | Bradi1g12650.1 | 551 | 4 | 424 | NP | NP | 2 | A,T,P |
|  | Bdi-miR396ab | Bradi4g16450.1 | 710 | 4 | 183 | NP | NP | 3.5 | A,T,P |
|  | Bdi-miR396ab | Bradi1g09900.1 | 335 | 3 | 127 | 3.5 | 2.5 | 3.5 | A,T |
|  | Bdi-miR396ab | Bradi2g11230.1 | 818 | 3 | 50 | 4 | 4 | NP | A,T |
|  | Bdi-miR396ab | Bradi2g45300.1 | 852 | 2 | 4 | 2 | 1.5 | NP | P |
|  | Bdi-miR396cd | Bradi3g52547.1 | 494 | 4 | 3853 | NP | NP | 3 | A,T,P |
|  | Bdi-miR396cd | Bradi3g57267.1 | 395 | 4 | 159 | NP | NP | 3 | A,T,P |
|  | Bdi-miR396cd | Bradi5g20607.1 | 497 | 4 | 3853 | NP | NP | 3 | A,T,P |
|  | Bdi-miR396cd | Bradi1g46427.1 | 392 | 4 | 129 | NP | NP | 3 | A,T,P |
|  | Bdi-miR396cd | Bradi1g50597.1 | 392 | 4 | 2171 | NP | NP | 3 | A,T,P |
|  | Bdi-miR396cd | Bradi2g45300.1 | 852 | 2 | 4 | 5 | 2 | NP | P |
|  | Bdi-miR396cd | Bradi1g44460.1 | 525 | 1 | 3 | 7 | 3 | NP |  |
|  | Bdi-miR396cd | Bradi3g12240.1 | 549 | 1 | 1 | 5 | 4 | NP |  |
|  | Bdi-miR396cd | Bradi5g21080.1 | 1116 | 1 | 4 | 6 | NP | NP |  |
|  | Bdi-miR396cd | Bradi3g53247.1 | 660 | 1 | 3 | 6.5 | NP | NP |  |
|  | Bdi-miR396e | Bradi4g16450.1 | 710 | 4 | 183 | NP | NP | 3 | A,T,P |
|  | Bdi-miR396e | Bradi1g46427.1 | 392 | 4 | 129 | NP | NP | 3 | A,T,P |
|  | Bdi-miR396e | Bradi1g50597.1 | 392 | 4 | 2171 | NP | NP | 3 | A,T,P |
|  | Bdi-miR396e | Bradi2g45300.1 | 852 | 2 | 4 | 6 | 3 | NP | P |
|  | Bdi-miR396e | Bradi5g21080.1 | 1116 | 1 | 4 | 5 | 4 | NP |  |
|  | Bdi-miR396e | Bradi1g45800.1 | 759 | 1 | 2 | NP | 4 | NP |  |
|  | Bdi-miR396e | Bradi3g14490.1 | 498 | 1 | 3 | NP | 4 | NP |  |
|  | Bdi-miR396e | Bradi3g12240.1 | 549 | 1 | 1 | 6 | NP | NP |  |
|  | Bdi-miR396e | Bradi3g53247.1 | 660 | 1 | 3 | 7 | NP | NP |  |
|  | Bdi-miR396e | Bradi4g34360.1 | 222 | 1 | 4 | NP | NP | 4.5 |  |
| 397 | Bdi-miR397ab | Bradi4g39330.1 | 690 | 2 | 17 | 5 | 2.5 | NP | A |
|  | Bdi-miR397ab | Bradi2g54690.1 | 687 | 1 | 1 | 4 | 2 | 3 |  |
|  | Bdi-miR397ab | Bradi3g45460.1 | 455 | 1 | 3 | NP | NP | 4.5 |  |
| 398 | Bdi-miR398b | Bradi5g23166.1 | 207 | 1 | 3 | 6.5 | 4 | NP |  |
| 399 | Bdi-miR399d | Bradi3g22020.1 | 383 | 1 | 1 | NP | NP | 4.5 |  |
| 408 | Bdi-miR408 | Bradi1g11240.1 | 16 | 1 | 1 | 5 | 3 | NP |  |
|  | Bdi-miR408 | Bradi3g03720.1 | 1258 | 1 | 1 | 7 | NP | NP |  |
|  | Bdi-miR408 | Bradi4g16750.1 | 534 | 1 | 3 | 7 | NP | NP |  |
|  | Bdi-miR408-5p | Bradi4g09417.1 | 395 | 2 | 31 | 4 | 2 | 3.5 | A |
|  | Bdi-miR408-5p | Bradi3g33080.1 | 1342 | 1 | 1 | 5.5 | 3.5 | NP |  |
|  | Bdi-miR408-5p | Bradi3g52150.1 | 1022 | 1 | 1 | 7 | 3.5 | NP |  |
| 444 | Bdi-miR444ab | Bradi3g46920.1 | 293 | 3 | 10 | 4 | 2 | 2 | A,T |
|  | Bdi-miR444ab | Bradi2g32290.1 | 797 | 3 | 10 | NP | NP | 2.5 | A,T |
|  | Bdi-miR444ab | Bradi2g41210.1 | 606 | 3 | 13 | 6.5 | NP | NP | A,T |
|  | Bdi-miR444ab | Bradi3g16220.1 | 992 | 3 | 9 | 1.5 | 1 | 1 | T,P |
|  | Bdi-miR444ab | Bradi3g08820.1 | 513 | 1 | 8 | 7 | NP | NP |  |
|  | Bdi-miR444ab | Bradi1g07170.1 | 333 | 1 | 8 | NP | NP | 4.5 |  |
|  | Bdi-miR444b.3 | Bradi3g57017.1 | 314 | 3 | 5 | NP | NP | 0 | T,P |
|  | Bdi-miR444cd | Bradi3g46920.1 | 293 | 3 | 10 | 0 | 0 | 0 | A,T |
|  | Bdi-miR444cd | Bradi2g32290.1 | 797 | 3 | 10 | NP | NP | 3.5 | A,T |
|  | Bdi-miR444cd | Bradi3g16220.1 | 992 | 3 | 9 | 3.5 | 2 | 2 | T,P |
|  | Bdi-miR444cd | Bradi1g64130.1 | 978 | 1 | 5 | 7 | 3 | NP |  |
|  | Bdi-miR444cd.2 | Bradi1g17050.1 | 295 | 1 | 1 | 7 | NP | NP |  |
|  | Bdi-miR444cd.2 | Bradi1g64130.1 | 972 | 1 | 7 | 7 | NP | NP |  |
| 528 | Bdi-miR528 | Bradi3g43070.1 | 92 | 2 | 51 | 6 | 3.5 | NP | A |
|  | Bdi-miR528 | Bradi4g28320.1 | 347 | 1 | 2 | 7 | 4 | NP |  |
| 530 | Bdi-miR530ab | Bradi3g41260.1 | 608 | 1 | 3 | 7 | NP | NP |  |
|  | Bdi-miR530ab | Bradi5g07410.1 | 164 | 1 | 1 | 7 | NP | NP |  |
|  | Bdi-miR530b-3p | Bradi4g40800.1 | 202 | 2 | 77 | 6.5 | 3.5 | NP | A |
|  | Bdi-miR530b-3p | Bradi1g04080.1 | 828 | 1 | 3 | NP | NP | 3 |  |
| 845 | Bdi-miR845 | Bradi5g12660.5 | 2903 | 1 | 2 | 6.5 | 3.5 | NP |  |
|  | Bdi-miR845 | Bradi2g15730.1 | 141 | 1 | 5 | NP | NP | 4.5 |  |
| 1432 | Bdi-miR1432 | Bradi1g53830.1 | 53 | 1 | 2 | 4.5 | 3.5 | 4.5 |  |
|  | Bdi-miR1432 | Bradi5g19240.1 | 851 | 1 | 1 | 4.5 | 2.5 | NP |  |
|  | Bdi-miR1432 | Bradi2g26930.1 | 999 | 1 | 11 | 7 | NP | NP |  |
|  | Bdi-miR1432 | Bradi2g12320.1 | 1062 | 1 | 2 | NP | NP | 5 |  |
|  | Bdi-miR1432 | Bradi4g20890.1 | 1467 | 1 | 1 | NP | NP | 5 |  |
| 2118 | Bdi-miR2118b | Bradi2g31500.1 | 553 | 3 | 2 | 6 | 4 | NP | T,P |
|  | Bdi-miR2118b | Bradi5g13180.1 | 1260 | 1 | 2 | NP | NP | 3.5 |  |
| 2275 | Bdi-miR2275a | Bradi5g24130.1 | 357 | 1 | 2 | 4.5 | 2.5 | 3 |  |
|  | Bdi-miR2275a | Bradi4g06017.1 | 615 | 1 | 1 | 4.5 | 4 | NP |  |
|  | Bdi-miR2275a | Bradi1g66620.1 | 1047 | 1 | 1 | NP | 4 | NP |  |
|  | Bdi-miR2275a | Bradi3g15290.1 | 801 | 1 | 1 | 6 | NP | NP |  |
|  | Bdi-miR2275a | Bradi3g43540.1 | 1122 | 1 | 2 | NP | NP | 4.5 |  |
|  | Bdi-miR2275a | Bradi2g20200.1 | 1359 | 1 | 1 | NP | NP | 4 |  |
|  | Bdi-miR2275b | Bradi2g20200.1 | 1359 | 1 | 1 | NP | NP | 3.5 |  |
|  | Bdi-miR2275b-5p | Bradi3g58280.1 | 325 | 1 | 1 | NP | NP | 5 |  |
|  | bdi-miR2275c-5p | Bradi1g23350.1 | 312 | 1 | 2 |  |  | 3.5 |  |
|  | bdi-miR2275c-5p | Bradi3g20460.1 | 1286 | 1 | 8 |  |  | 3.5 |  |
|  | bdi-miR2275c-3p | Bradi5g24130.1 | 357 | 1 | 2 | 4 | 2 | 2.5 |  |
| 5163 | Bdi-miR5163b-3p | Bradi4g10171.1 | 1746 | 2 | 3 | 3 | 1.5 | 2 | T |
|  | Bdi-miR5163b-3p | Bradi4g07430.1 | 1000 | 1 | 2 | 6.5 | NP | NP |  |
| 5169 | Bdi-miR5169ab | Bradi5g18060.1 | 220 | 1 | 1 | NP | NP | 4 |  |
| 5173 | Bdi-miR5173 | Bradi2g19360.1 | 313 | 1 | 2 | 7 | NP | NP |  |
|  | Bdi-miR5173 | Bradi3g38045.1 | 79 | 1 | 2 | NP | NP | 4.5 |  |
|  | Bdi-miR5173 | Bradi3g10530.1 | 1993 | 1 | 1 | NP | NP | 5 |  |
|  | Bdi-miR5173 | Bradi3g38040.1 | 79 | 1 | 2 | NP | NP | 5 |  |
| 5174 | Bdi-miR5174bdf | Bradi2g05530.1 | 1279 | 1 | 2 | NP | NP | 4.5 |  |
| 5176 | Bdi-miR5176 | Bradi3g05520.2 | 1119 | 1 | 7 | 6.5 | NP | NP |  |
| 5178 | bdi-miR5178 | Bradi3g05010.1 | 332 | 1 | 3 | NP | N | 4.5 |  |
| 5179 | Bdi-miR5179 | Bradi1g35000.1 | 371 | 1 | 3 | 1 | 1 | 1 |  |
|  | Bdi-miR5179 | Bradi1g68887.1 | 625 | 1 | 6 | NP | 4 | NP |  |
| 5181 | Bdi-miR5181c | Bradi1g52160.1 | 964 | 1 | 1 | 7 | NP | NP |  |
|  | Bdi-miR5181c | Bradi1g09110.1 | 864 | 1 | 2 | NP | NP | 4 |  |
|  | Bdi-miR5181c | Bradi1g33800.1 | 867 | 1 | 2 | NP | NP | 4 |  |
|  | Bdi-miR5181c | Bradi1g61230.2 | 349 | 1 | 1 | NP | NP | 4.5 |  |
|  | Bdi-miR5181c | Bradi3g14980.1 | 286 | 1 | 10 | NP | NP | 4.5 |  |
| 5185 | Bdi-miR5185l-3p.2 | Bradi1g25002.2 | 5313 | 1 | 2 | 6 | NP | NP |  |
| 5199 | bdi-miR5199 | Bradi5g21090.1 | 1959 | 1 | 1 | NP | N | 4.5 |  |
| 5201 | bdi-miR5201 | Bradi5g19840.1 | 759 | 1 | 3 | NP | N | 4 |  |
| 7738 | Bdi-miR7738-3p | Bradi1g73450.1 | 452 | 1 | 1 | NP | NP | 4 |  |
|  | Bdi-miR7738-3p | Bradi2g11370.1 | 262 | 1 | 1 | NP | NP | 4.5 |  |
|  | Bdi-miR7738-3p | Bradi3g17900.1 | 697 | 1 | 1 | NP | NP | 4.5 |  |
| 7754 | Bdi-miR7754-3p | Bradi1g56137.1 | 539 | 1 | 2 | NP | NP | 4.5 |  |
|  | Bdi-miR7754-3p | Bradi1g58260.1 | 2021 | 1 | 2 | NP | NP | 4.5 |  |
| 9408 | Bdi-miR9408ab | Bradi2g36550.2 | 1332 | 1 | 1 | NP | NP | 4 |  |
| 9481 | Bdi-miR9481a | Bradi3g07980.2 | 170 | 1 | 2 | 7 | NP | NP |  |
|  | Bdi-miR9481b | Bradi2g55210.1 | 989 | 1 | 2 | NP | NP | 4.5 |  |
| 9482 | Bdi-miR9482 | Bradi4g17410.2 | 1522 | 1 | 2 | NP | NP | 4.5 |  |
| 9484 | Bdi-miR9484 | Bradi2g25376.1 | 251 | 1 | 1 | 6 | NP | NP |  |
|  | Bdi-miR9484 | Bradi1g29267.1 | 2555 | 1 | 2 | NP | NP | 4.5 |  |
|  | Bdi-miR9484 | Bradi4g44838.1 | 1224 | 1 | 3 | NP | NP | 4.5 |  |
| 9486 | Bdi-miR9486a | Bradi3g48630.1 | 1438 | 1 | 2 | NP | NP | 5 |  |
|  | Bdi-miR9486b | Bradi3g02487.1 | 645 | 1 | 2 | NP | NP | 5 |  |
|  | Bdi-miR9486b | Bradi3g42710.1 | 153 | 1 | 3 | NP | NP | 5 |  |
| 9487 | Bdi-miR9487 | Bradi2g14310.1 | 563 | 1 | 1 | 5 | 2.5 | 3 |  |
|  | Bdi-miR9487 | Bradi2g47850.1 | 563 | 1 | 2 | 6 | 3 | 3.5 |  |
|  | Bdi-miR9487 | Bradi4g24740.1 | 1225 | 1 | 2 | NP | NP | 3.5 |  |
|  | Bdi-miR9487 | Bradi2g56320.1 | 811 | 1 | 4 | NP | NP | 4 |  |
| 9488 | Bdi-miR9488 | Bradi2g27360.1 | 34 | 1 | 2 | 7 | NP | NP |  |
|  | Bdi-miR9488 | Bradi4g36290.1 | 774 | 1 | 4 | NP | NP | 4.5 |  |
| 9489 | Bdi-miR9489 | Bradi1g15190.1 | 655 | 1 | 5 | 4 | 2.5 | 3.5 |  |
|  | Bdi-miR9489 | Bradi2g34650.1 | 196 | 1 | 1 | 6 | 2.5 | NP |  |
|  | Bdi-miR9489 | Bradi2g26240.1 | 1390 | 1 | 1 | 6 | 4 | NP |  |
|  | Bdi-miR9489 | Bradi2g54680.1 | 1387 | 1 | 1 | 6.5 | 4 | NP |  |
|  | Bdi-miR9489 | Bradi1g55120.1 | 13 | 1 | 4 | 6.5 | NP | NP |  |
| 9483 | Bdi-miR9493 | Bradi4g06340.1 | 646 | 1 | 5 | NP | NP | 4 |  |
| 9484 | Bdi-miR9494 | Bradi2g10617.1 | 782 | 1 | 2 | 2 | 2 | 2 |  |
|  | Bdi-miR9494 | Bradi3g37950.1 | 902 | 1 | 2 | 2 | 2 | 2 |  |
|  | Bdi-miR9494 | Bradi3g03280.1 | 902 | 1 | 1 | 4 | 2.5 | 3 |  |
|  | Bdi-miR9494 | Bradi2g56970.1 | 857 | 1 | 1 | 5 | 3 | NP |  |
|  | Bdi-miR9494 | Bradi3g31850.1 | 629 | 1 | 8 | 7 | 3.5 | NP |  |
|  | Bdi-miR9494 | Bradi5g23325.1 | 186 | 1 | 3 | 7 | 3.5 | NP |  |
|  | Bdi-miR9494 | Bradi2g02730.1 | 324 | 1 | 1 | NP | 4 | NP |  |
|  | Bdi-miR9494 | Bradi5g15540.2 | 503 | 1 | 5 | 7 | NP | NP |  |
|  | Bdi-miR9494 | Bradi3g38410.1 | 788 | 1 | 1 | NP | NP | 2.5 |  |
| 9486 | Bdi-miR9496 | Bradi3g49100.1 | 1232 | 1 | 1 | 6 | NP | NP |  |
|  | Bdi-miR9496 | Bradi1g28310.3 | 1179 | 1 | 4 | 7 | NP | NP |  |
|  | Bdi-miR9496 | Bradi3g52400.1 | 501 | 1 | 2 | NP | NP | 4 |  |
| 9487 | Bdi-miR9497 | Bradi5g08235.1 | 2483 | 1 | 1 | 7 | 3.5 | NP |  |
| 9498 | Bdi-miR9498 | Bradi2g11220.1 | 373 | 1 | 4 | 6 | 3 | NP |  |
|  | Bdi-miR9498 | Bradi2g21120.1 | 411 | 1 | 8 | 5.5 | NP | NP |  |
|  | Bdi-miR9498 | Bradi4g03787.1 | 1773 | 1 | 1 | NP | NP | 4.5 |  |
| 9499 | Bdi-miR9499 | Bradi5g13600.1 | 110 | 1 | 2 | 5 | 3 | NP |  |
|  | Bdi-miR9499 | Bradi2g45740.1 | 245 | 1 | 4 | 3 | 3.5 | NP |  |
|  | Bdi-miR9499 | Bradi1g72450.1 | 992 | 1 | 15 | 6 | 3.5 | NP |  |
|  | Bdi-miR9499 | Bradi1g63610.1 | 371 | 1 | 1 | 7 | NP | NP |  |
|  | |  |  |  |  |  |  |  |  |

.

**Table S4. Arabidopsis miRNA Targets.**

| miRNA(s) | Gene | Reference |
| --- | --- | --- |
| miR156 | AT5G38610 | 9 |
| miR156/7 | AT1G27370 | 1 |
| miR156/7 | AT3G15270 | 9 |
| miR156a | AT5G50570 | 9 |
| miR156g | AT1G27360 | 9 |
| miR156g | AT1G53160 | 1 |
| miR156g | AT1G69170 | 2 |
| miR156g | AT2G33810 | 1 |
| miR156g | AT2G42200 | 9 |
| miR156g | AT3G57920 | 2 |
| miR156g | AT5G43270 | 1 |
| miR159a | AT5G18100 | 2 |
| miR159b | AT2G34010 | 2 |
| miR159b | AT3G11440 | 3 |
| miR159b | AT5G06100 | 3 |
| miR160c | AT1G77850 | 1 |
| miR160c | AT2G28350 | 1 |
| miR160c | AT4G30080 | 1 |
| miR161 | AT1G06580 | 1 |
| miR161 | AT1G62590 | 9 |
| miR161 | AT1G63080 | 1 |
| miR161 | AT1G63130 | 4 |
| miR161 | AT1G63230 | 9 |
| miR161 | AT1G63330 | 9 |
| miR161 | AT1G63400 | 4 |
| miR161.a2 | AT5G41170 | 1 |
| miR161a.1 | AT1G63150 | 1 |
| miR161a.1 | AT5G41170 | 1 |
| miR161a.2 | AT1G63150 | 1 |
| miR162 | AT1G01040 | 5 |
| miR163 | AT1G15125 | 6 |
| miR163 | AT1G66690 | 6 |
| miR163 | AT1G66700 | 6 |
| miR163 | AT1G66720 | 6 |
| miR163 | AT3G44860 | 6 |
| miR164 | AT1G56010 | 1 |
| miR164b | AT3G15170 | 1 |
| miR164b | AT5G07680 | 1 |
| miR164b | AT5G39610 | 5 |
| miR164b | AT5G53950 | 1 |
| miR164b | AT5G61430 | 1 |
| miR165 | AT1G30490 | 1 |
| miR165 | AT2G34710 | 1 |
| miR165 | AT4G32880 | 1 |
| miR165a | AT5G60690 | 1 |
| miR166 | AT1G52150 | 1 |
| miR167 | AT1G30330 | 5 |
| miR167d | AT5G37020 | 1 |
| miR168 | AT1G48410 | 1 |
| miR168a | AT3G58030 | 2 |
| miR169f | AT1G17590 | 1 |
| miR169n | AT1G54160 | 1 |
| miR169n | AT1G72830 | 5 |
| miR169n | AT3G05690 | 5 |
| miR169n | AT5G06510 | 5 |
| miR169n | AT5G12840 | 2 |
| miR170 | AT3G60630 | 1 |
| miR170 | AT4G00150 | 1 |
| miR171a | AT2G45160 | 7 |
| miR171b | AT3G60630 | 1 |
| miR171b | AT4G00150 | 1 |
| miR172a | AT5G60120 | 5 |
| miR172e | AT2G28550 | 5 |
| miR172e | AT2G39250 | 2 |
| miR172e | AT3G54990 | 2 |
| miR172e | AT4G36920 | 5 |
| miR172e | AT5G67180 | 5 |
| miR173 | AT1G50055 | 4 |
| miR173 | AT2G27400 | 4 |
| miR173 | AT2G39675 | 4 |
| miR173 | AT2G39681 | 8 |
| miR173* | AT5G10950 | 9 |
| miR319 | AT1G30210 | 3 |
| miR319c | AT1G53230 | 3 |
| miR319c | AT2G31070 | 3 |
| miR319c | AT3G15030 | 3 |
| miR319c | AT4G18390 | 3 |
| miR390 | AT3G17185 | 8 |
| miR390a | AT5G49615 | 8 |
| miR390b | AT5G57735 | 8 |
| miR393 | AT1G12820 | 5 |
| miR393 | AT4G03190 | 5 |
| miR393b | AT3G23690 | 5 |
| miR393b | AT3G26810 | 5 |
| miR393b | AT3G62980 | 5 |
| miR394 | AT1G27340 | 5 |
| miR395a | AT4G14680 | 8 |
| miR395e | AT3G22890 | 5 |
| miR395e | AT5G10180 | 5 |
| miR395e | AT5G43780 | 5 |
| miR396 | AT1G10120 | 2 |
| miR396 | AT2G22840 | 5 |
| miR396 | AT2G45480 | 5 |
| miR396 | AT4G24150 | 5 |
| miR396 | AT4G37740 | 5 |
| miR396a | AT2G36400 | 5 |
| miR396a | AT5G53660 | 5 |
| miR397b | AT2G29130 | 5 |
| miR397b | AT2G38080 | 5 |
| miR397b | AT3G60250 | 2 |
| miR397b | AT5G60020 | 5 |
| miR398 | AT3G15640 | 5 |
| miR398a | AT1G08830 | 9 |
| miR398a | AT2G28190 | 5 |
| miR399e | AT2G33770 | 5 |
| miR400 | AT1G06580 | 9 |
| miR403 | AT1G31280 | 10 |
| miR408 | AT1G72230 | 2 |
| miR408 | AT2G02850 | 2 |
| miR408 | AT2G30210 | 10 |
| miR408 | AT2G44790 | 2 |
| miR447a | AT5G60760 | 10 |
| miR472 | AT1G51480 | 11 |
| miR472 | AT5G43740 | 11 |
| miR773 | AT4G14140 | 11 |
| miR774 | AT3G19890 | 11 |
| miR775 | AT1G53290 | 10 |
| miR778 | AT2G22740 | 11 |
| miR780 | AT5G41610 | 11 |
| miR822 | AT5G02350 | 9 |
| miR823 | AT1G69770 | 2 |
| miR824 | AT3G14560 | 2 |
| miR824 | AT3G57230 | 10 |
| miR827 | AT1G02860 | 10 |
| miR837-3p | AT5G08415 | 9 |
| miR842 | AT5G38550 | 10 |
| miR844 | AT5G51270 | 11 |
| miR846 | AT5G49850 | 11 |
| miR856 | AT5G41610 | 11 |
| miR857 | AT3G09220 | 11 |
| miR858 | AT1G06180 | 2 |
| miR858 | AT1G66230 | 2 |
| miR858 | AT2G47460 | 11 |
| miR858 | AT3G08500 | 11 |
| miR858 | AT5G49330 | 2 |
| miR859 | AT3G49510 | 11 |
| miR863-3p | AT4G13495 | 9 |

1. Rhoades MW, Reinhart BJ, Lim LP, Burge CB, Bartel B, Bartel DP: Prediction of plant microRNA targets. *Cell* 2002,110:513.

2. German MA, Pillay M, Jeong DH, Hetawal A, Luo S, Janardhanan P, Kannan V, Rymarquis LA, Nobuta K, German R, De Paoli E, Lu C, Schroth G, Meyers BC, Green PJ: Global identification of microRNA-target pairs by parallel analysis of RNA ends. *Nature Biotech* 2008,26:941.

3. Palatnik JF, Wollmann H, Schommer C, Schwab R, Boisbouvier J, Rodriguex R, Warthmann N, Allens E, Dezulian T, Huson D, Carrington JC, Weigel D: Sequence and expression differences underlie functional specialization of *Arabidopsis* microRNAs miR159 and miR319. *Dev Cell* 2007,13:115.

4. Howell, MD. Genome-wide analysis of the RNA-Dependent RNA Polymerase 6/Dicer-Like 4 pathway in Arabidopsis reveals dependency on miRNA- and tasiRNA-directed targeting. *Plant Cell* 2007,19:926.

5. Rhoades MW, Bartel P: Computational identification of plant microRNAs and their targets, including a stress-induced miRNA. *Mol Cell* 2004,14:787.

6. Xie Z, Allen E, Fahlgren N, Clamar A, Givan SA, Carrington JC: Expression of Arabidopsis miRNA genes. *Plant Phys* 2005,18:2145.

7. Souret FF, Kastenmayer JP, Green PJ: AtXRN4 degrades mRNA in Arabidopsis and its substrates include selected miRNA targets. *Mol Cell* 2004,15:173.

8. Allen EA, Xie Z, Gustafson AM, Carrington JC: MicroRNA-directed phasing during trans-acting siRNA biogenesis in plants. *Cell* 2005,121:207.

9. Addo-Quaye C, Eshoo TW, Bartel DP, Axtell MJ: Exogenous siRNA and miRNA targets identified by sequencing of the *Arabidopsis* degradome. *Curr Biol* 2008,18:758.

10. Rajogopalan R, Vaucheret J, Trejo J, Bartel DP: A diverse and evolutionarily fluid set of microRNAs in Arabidopsis thaliana. *Genes Dev* 2006,20:3407.

11. Fahlgren N, Howell MD, Kasschau KD, Chapman EJ, Sullivan CM, Cumbie JS, Givan SA, Law TF, Grant SR, Dangl JL, Carrington JC: High-throughput sequencing of *Arabidopsis* microRNAs: Evidence for frequent birth and death of miRNA genes. *PLoS ONE* 2007,2: e219. doi:10.1371/journal.pone.0000219.

**Table S5. Oligomers Used in This Study**

| **Name** | **Sequence** | **Purpose** |
| --- | --- | --- |
| Bdi-FTL1 (Bradi1g48830) Forward | ATGCTCCTAGTCCAAGCGAC | Oligomer to amplify gene specific probe |
| Bdi-FTL1 (Bradi1g48830) Reverse | AGAGCTCGGCGAAGTCCCTG | Oligomer to amplify gene specific probe |
| Bdi-miR162 | CCGGATGCAGAGGTTTATCGA | miRNA probe |
| Ath-miR162ab, Osa-miR162a | CTGGATGCAGAGGTTTATCGA | miRNA probe |
| miR166 | GGGGAATGAAGCCTGGTCCGA | miRNA probe |
| Bridgeoligo of Bdi-miR399 | gaatgtcataagcgCCGGGCAAATCTCCTTTGGCA | Bridge oligonucleotide to detect miRNA |
| Bridgeoligo of Bdi-miR827 | gaatgtcataagcgTGTTTGCTGATGGTCATCTAA | Bridge oligonucleotide to detect miRNA |
| Bridgeoligo of Bdi-miR168 | gaatgtcataagcgGTCCCGATCTGCACCAAGCGA | Bridge oligonucleotide to detect miRNA |
| Bridgeoligo of Bdi-miR5200 | gaatgtcataagcgAAGCCTTAGGGAGTATCTACA | Bridge oligonucleotide to detect miRNA |
| Bridgeoligo of Bdi-miR166a-eg | gaatgtcataagcgGGGGAATGAAGCCTGGTCCGA | Bridge oligonucleotide to detect miRNA |
| Bridgeoligo of Bdi-miR166h | gaatgtcataagcgAGGGATTGAAGCCTGGTCCGA | Bridge oligonucleotide to detect miRNA |
| Bridgeoligo of Bdi-miR166f | gaatgtcataagcgGGAATGAAGCCTGGTCCGAGA | Bridge oligonucleotide to detect miRNA |
| Bridgeoligo of Bdi-miR156a-h | gaatgtcataagcgGTGCTCACTCTCTTCTGTCA | Bridge oligonucleotide to detect miRNA |
| Bridgeoligo of Bdi-miR529 | gaatgtcataagcgAGGCTGTACTCTCTCTCTTCT | Bridge oligonucleotide to detect miRNA |
