# Supplementary material for: Parallel analysis of RNA ends enhances global investigation of microRNAs and target RNAs of Brachypodium distachyon
Source: Genome Biol. 2013 Dec 24;14(12):R145. doi: 10.1186/gb-2013-14-12-r145 (PMC4053937; doi:10.1186/gb-2013-14-12-r145)
Supplement: Additional file 2: Figure S1 — Predicted secondary structure of Bdi-MIR444 precursors. Figure S2. Target prediction programs identified distinct subsets of targets. Figure S3. Representative D-plots for miRNA targets at each stringency level. Figure S4. Bdi-miR2118 cleavage induces phasing. Figure S5. Bdi-miR5200 in diverse plant species. Figure S6. D-plots of AGO1 for Arabidopsis, rice and Brachypodium. [file gb-2013-14-12-r145-S2.pptx]

## Slide 1
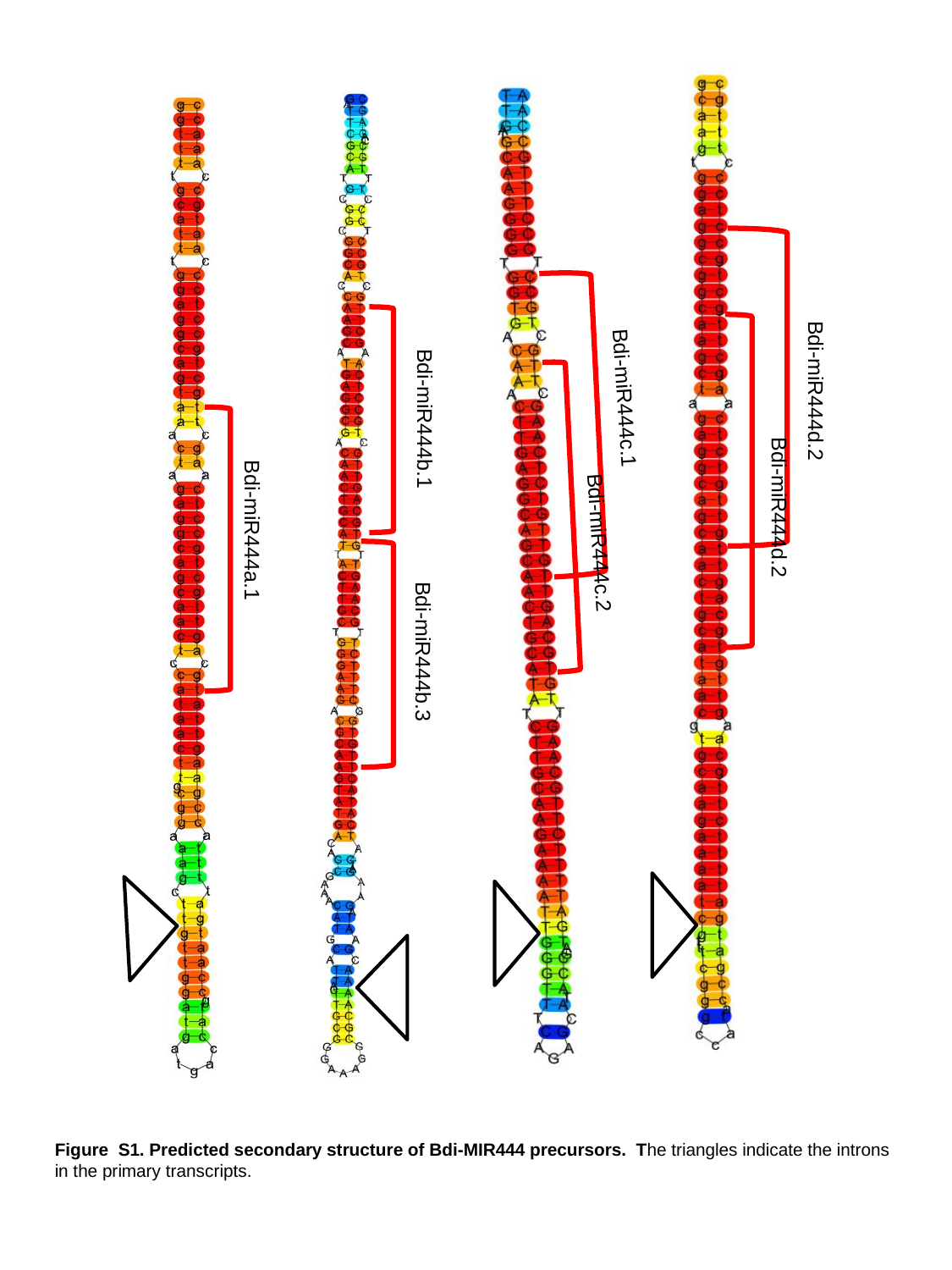

Bdi-miR444d.2
Bdi-miR444c.1
Bdi-miR444b.1
Bdi-miR444d.2
Bdi-miR444a.1
Bdi-miR444c.2
Bdi-miR444b.3
Figure S1. Predicted secondary structure of Bdi-MIR444 precursors. The triangles indicate the introns in the primary transcripts.

## Slide 2
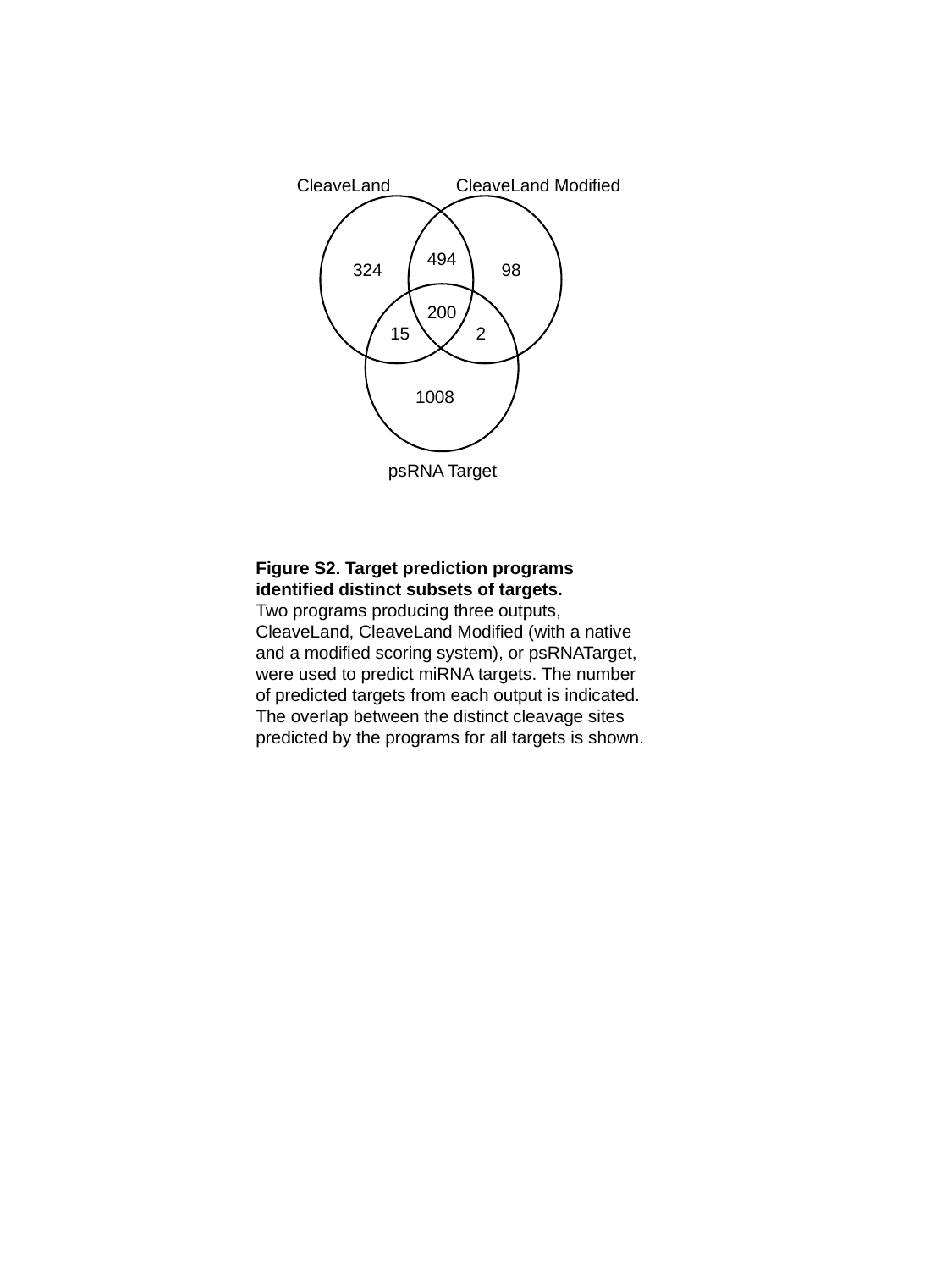

CleaveLand
CleaveLand Modified
494
324
98
200
15
2
1008
psRNA Target
Figure S2. Target prediction programs identified distinct subsets of targets.
Two programs producing three outputs, CleaveLand, CleaveLand Modified (with a native and a modified scoring system), or psRNATarget, were used to predict miRNA targets. The number of predicted targets from each output is indicated. The overlap between the distinct cleavage sites predicted by the programs for all targets is shown.

## Slide 3
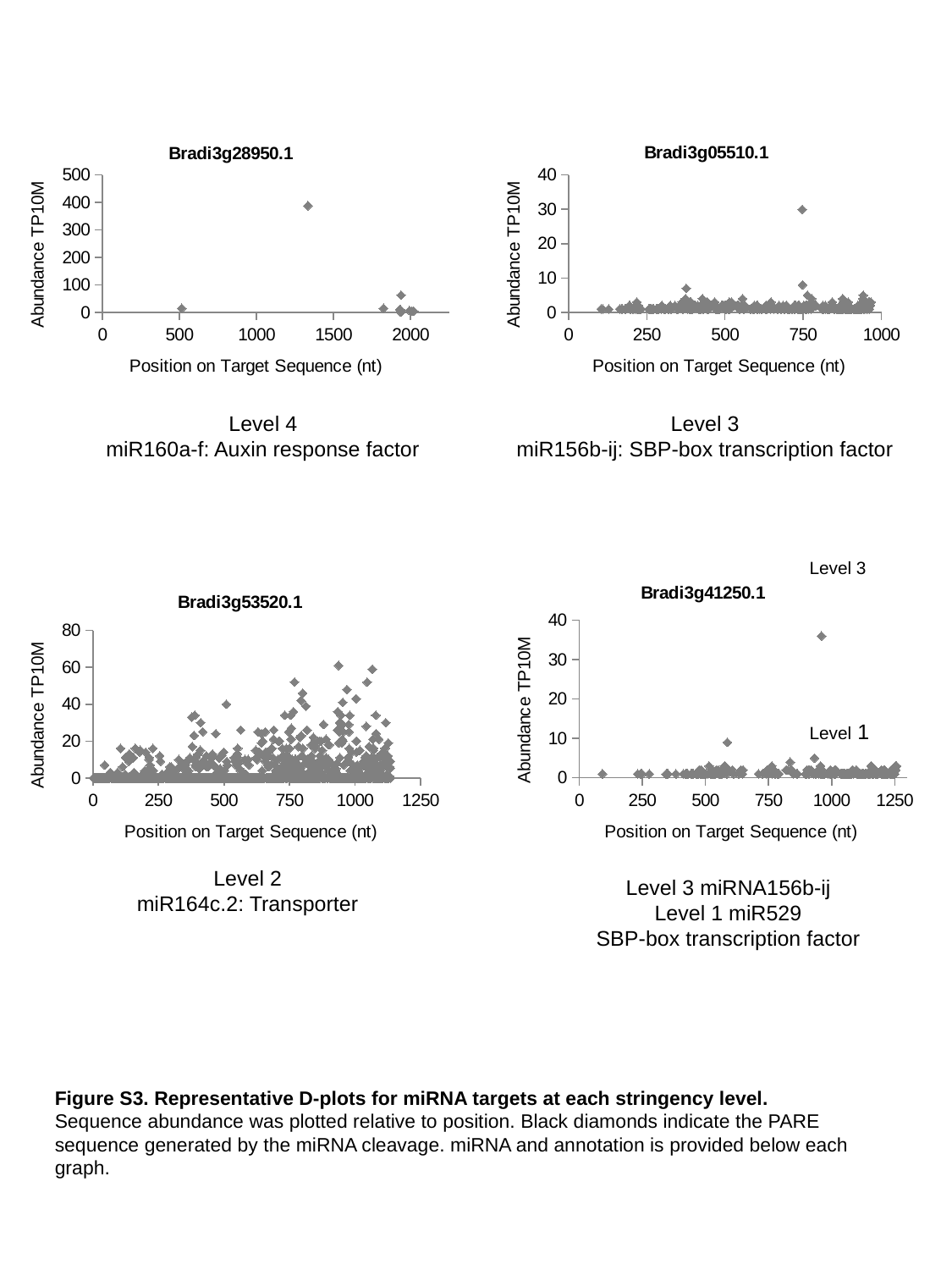

### Chart: Bradi3g28950.1
| Category | |
|---|---|Level 4
miR160a-f: Auxin response factor
### Chart: Bradi3g05510.1
| Category | |
|---|---|Level 3
miR156b-ij: SBP-box transcription factor
Level 3
### Chart: Bradi3g41250.1
| Category | |
|---|---|Level 1
Level 3 miRNA156b-ij
Level 1 miR529
SBP-box transcription factor
### Chart: Bradi3g53520.1
| Category | |
|---|---|Level 2
miR164c.2: Transporter
Figure S3. Representative D-plots for miRNA targets at each stringency level. Sequence abundance was plotted relative to position. Black diamonds indicate the PARE sequence generated by the miRNA cleavage. miRNA and annotation is provided below each graph.

## Slide 4
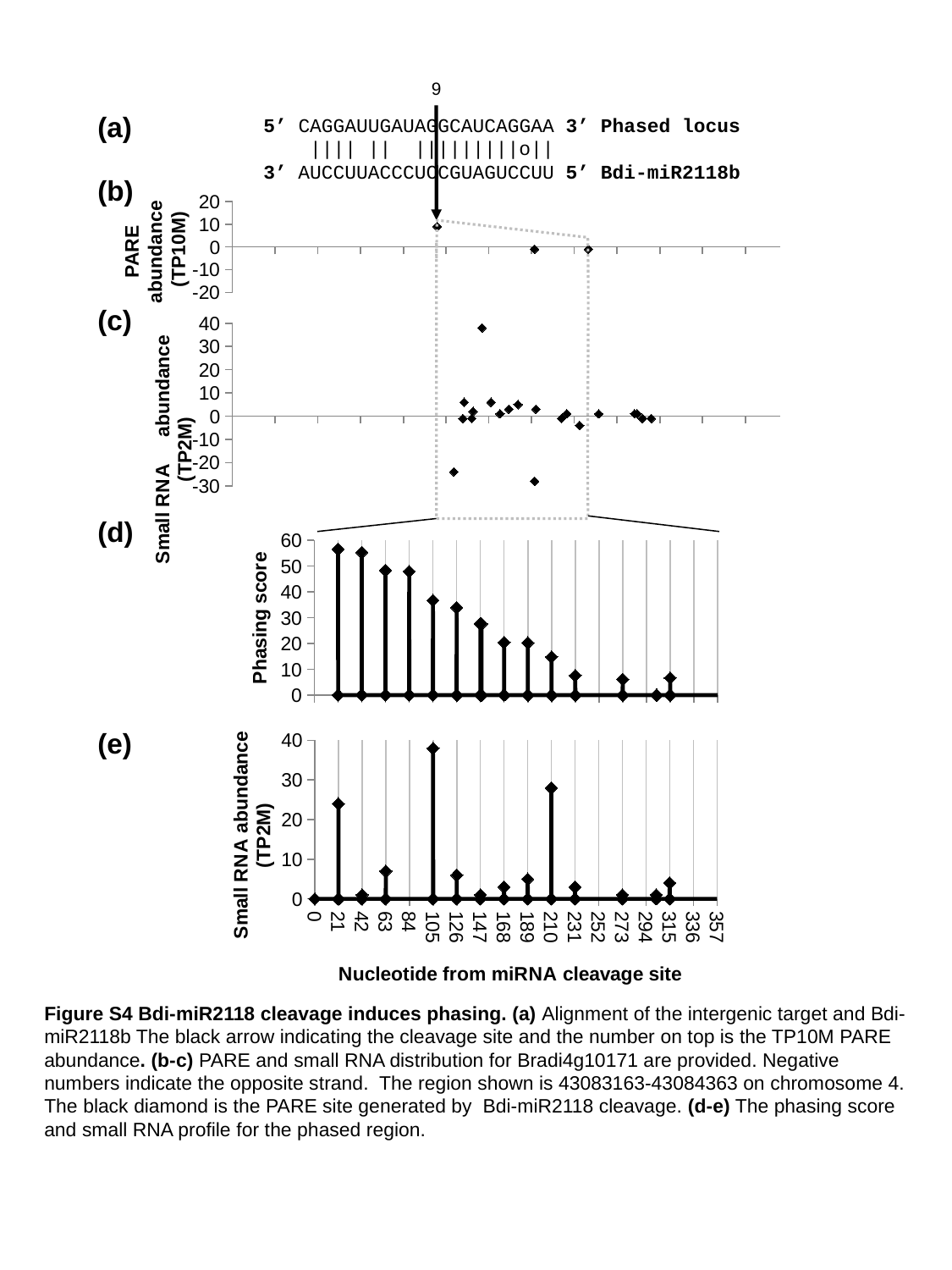

9
5’ CAGGAUUGAUAGGCAUCAGGAA 3’ Phased locus
 |||| || |||||||||o||
3’ AUCCUUACCCUCCGUAGUCCUU 5’ Bdi-miR2118b
(a)
(b)
### Chart
| Category | |
|---|---|PARE
 abundance
 (TP10M)
(c)
### Chart
| Category | |
|---|---|(d)
### Chart
| Category | |
|---|---|(e)
### Chart
| Category | |
|---|---|Figure S4 Bdi-miR2118 cleavage induces phasing. (a) Alignment of the intergenic target and Bdi-miR2118b The black arrow indicating the cleavage site and the number on top is the TP10M PARE abundance. (b-c) PARE and small RNA distribution for Bradi4g10171 are provided. Negative numbers indicate the opposite strand. The region shown is 43083163-43084363 on chromosome 4. The black diamond is the PARE site generated by Bdi-miR2118 cleavage. (d-e) The phasing score and small RNA profile for the phased region.

## Slide 5
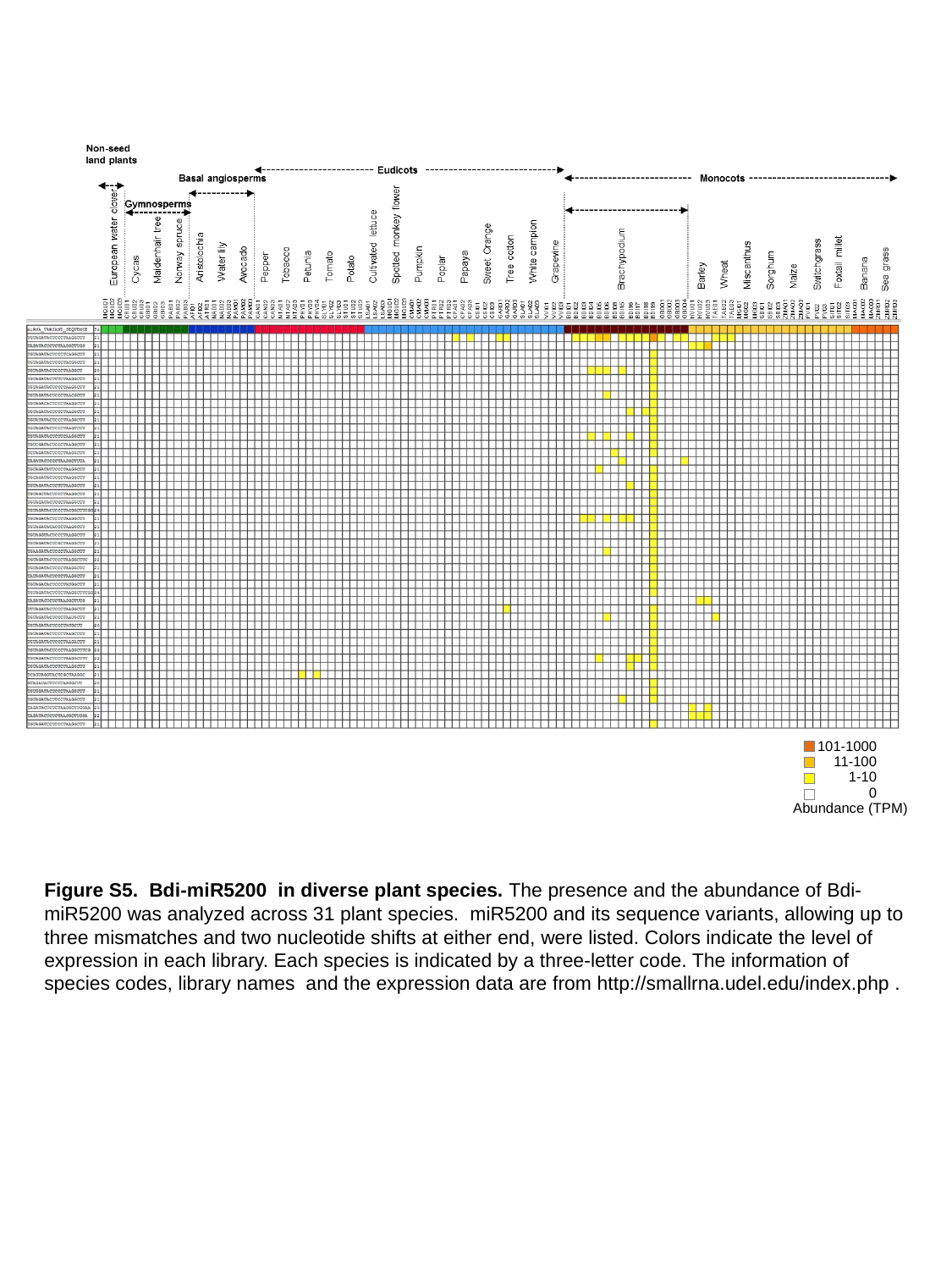

101-1000
11-100
1-10
0
Abundance (TPM)
Figure S5. Bdi-miR5200 in diverse plant species. The presence and the abundance of Bdi-miR5200 was analyzed across 31 plant species. miR5200 and its sequence variants, allowing up to three mismatches and two nucleotide shifts at either end, were listed. Colors indicate the level of expression in each library. Each species is indicated by a three-letter code. The information of species codes, library names and the expression data are from http://smallrna.udel.edu/index.php .

## Slide 6
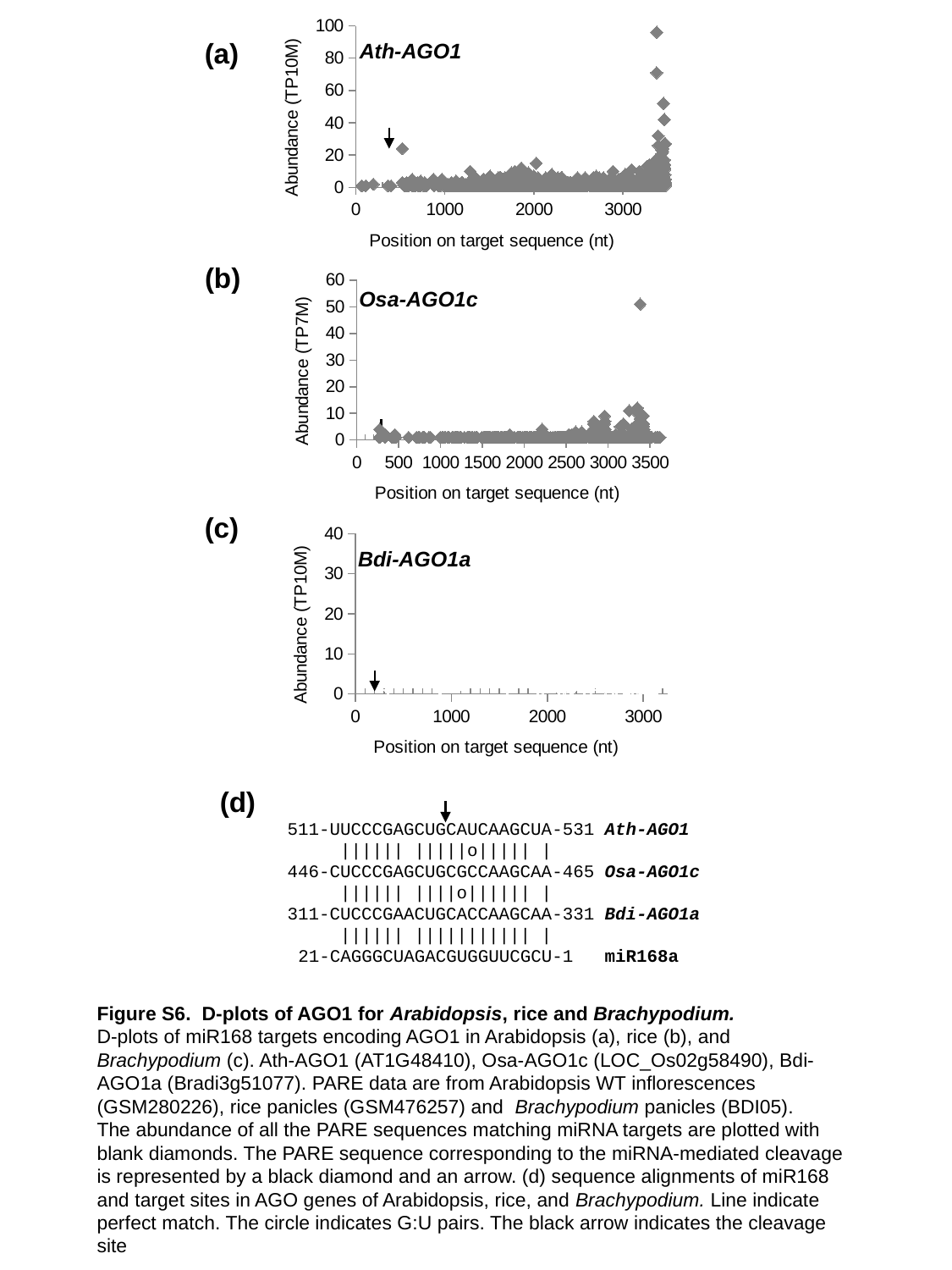

### Chart
| Category | TWF-Abundance |
|---|---|(a)
Ath-AGO1
(b)
### Chart
| Category | INF9311a-Abundance |
|---|---|Osa-AGO1c
(c)
### Chart
| Category | |
|---|---|Bdi-AGO1a
(d)
511-UUCCCGAGCUGCAUCAAGCUA-531 Ath-AGO1
 |||||| |||||o||||| |
446-CUCCCGAGCUGCGCCAAGCAA-465 Osa-AGO1c
 |||||| ||||o|||||| |
311-CUCCCGAACUGCACCAAGCAA-331 Bdi-AGO1a
 |||||| ||||||||||| |
 21-CAGGGCUAGACGUGGUUCGCU-1 miR168a
Figure S6. D-plots of AGO1 for Arabidopsis, rice and Brachypodium.
D-plots of miR168 targets encoding AGO1 in Arabidopsis (a), rice (b), and Brachypodium (c). Ath-AGO1 (AT1G48410), Osa-AGO1c (LOC_Os02g58490), Bdi-AGO1a (Bradi3g51077). PARE data are from Arabidopsis WT inflorescences (GSM280226), rice panicles (GSM476257) and Brachypodium panicles (BDI05).
The abundance of all the PARE sequences matching miRNA targets are plotted with blank diamonds. The PARE sequence corresponding to the miRNA-mediated cleavage is represented by a black diamond and an arrow. (d) sequence alignments of miR168 and target sites in AGO genes of Arabidopsis, rice, and Brachypodium. Line indicate perfect match. The circle indicates G:U pairs. The black arrow indicates the cleavage site
